# Nanometer-accuracy distance measurements between fluorophores at the single-molecule level

**DOI:** 10.1073/pnas.1815826116

**Published:** 2019-02-15

**Authors:** Stefan Niekamp, Jongmin Sung, Walter Huynh, Gira Bhabha, Ronald D. Vale, Nico Stuurman

**Affiliations:** ^a^Department of Cellular and Molecular Pharmacology, University of California, San Francisco, CA 94158;; ^b^Howard Hughes Medical Institute, University of California, San Francisco, CA 94158;; ^c^Skirball Institute of Biomolecular Medicine, New York University School of Medicine, New York, NY 10016;; ^d^Department of Cell Biology, New York University School of Medicine, New York, NY 10016

**Keywords:** superresolution, total internal reflection microscopy, fluorescence, single molecule, dynein

## Abstract

Light microscopy is a powerful tool for probing the conformations of molecular machines at the single-molecule level. Single-molecule Förster resonance energy transfer can measure intramolecular distance changes of single molecules in the range of 2 to 8 nm. However, current superresolution measurements become error-prone below 25 nm. Thus, new single-molecule methods are needed for measuring distances in the 8- to 25-nm range. Here, we describe methods that utilize information about localization and imaging errors to measure distances between two different color fluorophores with ∼1-nm accuracy at distances >2 nm. These techniques can be implemented in high throughput using a standard total internal reflection fluorescence microscope and open-source software. We applied our two-color localization method to uncover an unexpected ∼4-nm nucleotide-dependent conformational change in the coiled-coil “stalk” of the motor protein dynein. We anticipate that these methods will be useful for high-accuracy distance measurements of single molecules over a wide range of length scales.

## Significance

Measurements of macromolecular shapes provide insight into the mechanism of molecular machines. Distance measurements at the scale of biological macromolecules are often pursued by single-molecule fluorescence techniques. However, while single-molecule Förster resonance energy transfer can estimate distances of less than 8 nm, distances on the scale of 8 to 25 nm are difficult to determine. Here, we report two-color fluorescent distance measurement techniques capable of determining distances with ∼1-nm accuracy over a wide range of length scales. These methods can be implemented in high throughput on commonly available microscopes. As an example of their utility, we used our methods to uncover an unexpected conformational change in the antiparallel coiled-coil stalk of the dynein motor domain in different nucleotide states.

Understanding the spatial arrangement of biological macromolecules is crucial for elucidating molecular mechanisms. While 3D structures provide insight into the mechanism of a protein, the static state alone is often insufficient to understand how macromolecular machines perform action. By labeling single molecules or complexes at defined sites with fluorescent dyes, it is possible to obtain static or dynamic distance measurements that provide information about conformational changes or molecular interactions.

**Fig. 1.**
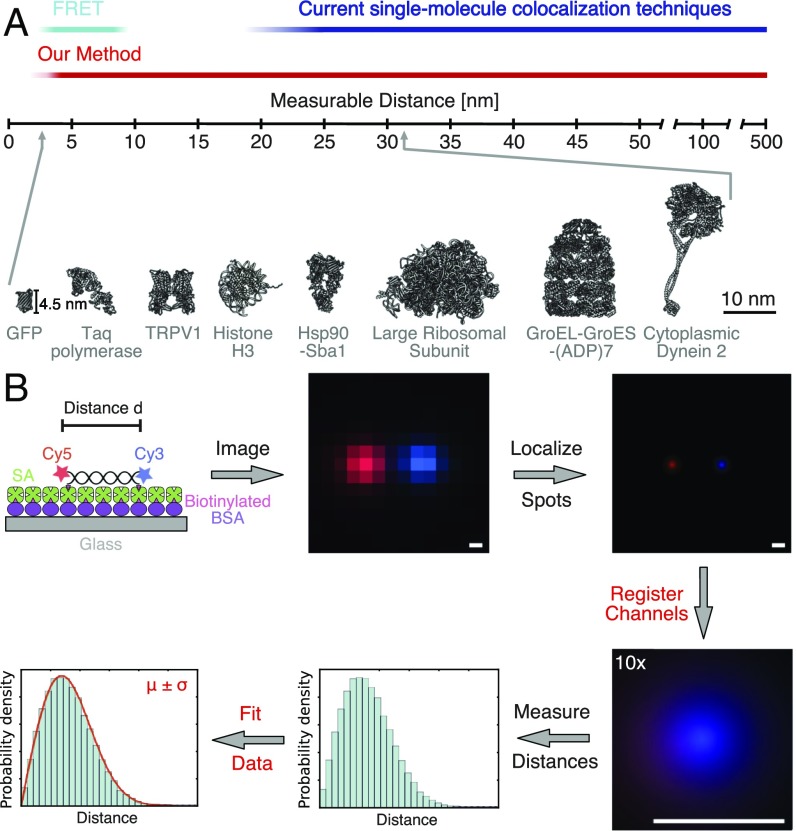
Relevance and workflow of fluorescent single-molecule distance measurements. (*A*) Comparison of resolution of various methods for fluorescent single-molecule distance measurements (*Top*). Size distribution of protein structures (*Bottom*). Protein Data Bank (PDB) ID codes from left to right: 1gfl (45), 1taq (46), 5irz (47), 1aoi (48), 2cg9 (49), 1jj2 (50), 1aon (51), 4rh7 (30). (*B*) Workflow for two-color distance measurements. First, the sample of interest is labeled at specific sites with two fluorescent dyes (Cy3 and Cy5), immobilized via biotin-streptavidin (SA) onto a glass coverslip, and imaged with a TIRF microscope. Then, the exact positions of the fluorophores are determined and the positions of both dyes are registered (aligned) utilizing a registration map that was previously determined. Subsequently, the distances of all spot pairs are measured and the average distance between fluorophores is determined using a fit of a probability distribution function to the data. (Scale bar: 100 nm.)

A widely used method for obtaining such distance information is single-molecule Förster resonance energy transfer (smFRET) (1) between two differently colored fluorophores. However, smFRET is limited to a narrow distance range, typically 2 to 8 nm. The calculation of absolute distances is influenced by the orientation and chemical environment of the fluorophores (2), which are difficult to measure, and hence smFRET is most widely used to detect relative distance changes. Direct fluorescent-based measurements of longer distances can be achieved by single-molecule colocalization microscopy (3–5), but distances below ∼25 nm have proven to be very difficult to measure correctly. Thus, there is an existing gap in resolution (Fig. 1*A*) that is important to fill since it corresponds to the size distribution of many proteins and protein complexes.

Previous studies have made considerable progress in tackling distance measurements between 8 and 25 nm. Single-molecule high-resolution colocalization (SHREC) (3, 6) resolves nanometer distances by accounting for localization errors when measuring the separation between two differently colored fluorophores. Pertsinidis et al. (7) developed a feedback-controlled system that enabled distance measurements with subnanometer precision, and Mortensen et al. (8) reported ∼1-nm resolution by imaging the same single molecules multiple times. However, distance measurements with nanometer accuracy and precision have not been more broadly adopted, either because these available methods suffer from inaccuracy and/or low throughput or they involve highly specialized optical setups (7).

Here, we report methods capable of reliably measuring two-color fluorophore distances at ∼1-nm accuracy over a wide range of distances (from ∼2 nm to hundreds of nanometers) using readily available microscope hardware. To achieve this level of accuracy, we first correct for chromatic aberrations and distortions using a piecewise affine transformation (9), yielding registration errors (image alignment of different fluorophores) of less than 1 nm over the entire field of view of a standard total internal reflection fluorescence (TIRF) microscope. We show that existing distance analysis methods, like those of Churchman et al. (6), become error-prone when the true distance and localization errors of the individual probes are similar, which is common for distances of ∼2 to 30 nm. To overcome these limitations, we developed two related methods: Sigma-P2D, which incorporates information about localization and imaging errors, and Vector-P2D, which makes use of averaging multiple observations of the same molecule. We applied our methods to investigate nucleotide-dependent conformational changes of the molecular motor dynein (10–12) and found that the stalk of dynein likely undergoes a large conformational change during its hydrolysis cycle (13). These results could not have been obtained by smFRET or other direct two-color imaging methods, since the distances measured changed from ∼16 nm to ∼20 nm in different nucleotide states. Thus, the two methods presented here, together with our improved image registration procedure, should have broad applications for inter- and intramolecular distance measurements, particularly in the range of 8 to 25 nm, where current techniques for two-color imaging are suboptimal. Our methods are also easily implemented using commercially available microscopes and open-source μManager (14) software.

## Results

### Registration Error in Subnanometer Range.

To achieve highly accurate distance measurements between two fluorophores that emit at different wavelengths, multiple obstacles have to be overcome. First, the sample of interest needs to be fluorescently labeled at specific sites and immobilized to the coverslip surface at a defined orientation (Fig. 1*B*). Then, one needs to image two channels, localize the individual probes, align the two channels (image registration), and calculate the distance between centroids from multiple observations of the same or multiple particles (Fig. 1*B*). While localization of individual fluorophores by fitting a point-spread function or 2D Gaussian to the fluorophore’s intensity distribution has been well established and delivers precision close to the theoretical limit (15), current image registration methods correct poorly for commonly observed chromatic aberrations over the entire field of view at the nanometer scale (7) or have problems in throughput since they are limited to imaging one pixel at time (8). Thus, to enable high-throughput and accurate two-color distance measurements, we first set out to improve two-channel image registration over the entire field of view.

**Fig. 2.**
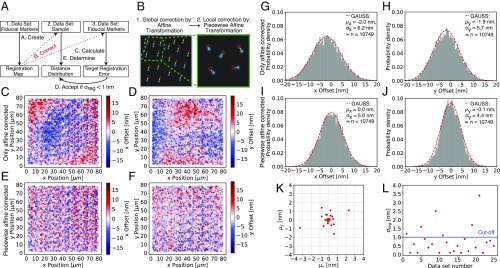
Image registration workflow, accuracy, and reproducibility. (*A*) Workflow of image acquisition and registration process. (*B*) Procedure for image registration with affine (global) and piecewise affine (local) correction. Comparing results for the affine correction (*C*, *D*, *G*, and *H*) and for the affine correction followed by piecewise affine correction (*E*, *F*, *I*, and *J*) shows that an additional piecewise affine correction reduces local distortions and results in better image registration overall. (*C*) TRE after affine correction along the *x* axis. Each dot shows a single fiducial marker for which the distance offset between the two colors of the same fiducial marker is color-coded. Negative values (blue dots) mean that channel 1 has a smaller number for its *x* position whereas positive values (red dots) represent fiducials where channel 2 has a smaller number for its *x* position. (*D*) Same dataset as in *C* but the offset is along the *y* axis. (*E*) TRE after piecewise affine correction along the *x* axis for the same beads as in *C*. (*F*) Same dataset as in *E* but the offset is along the *y* axis. (*G*) Histogram of *x* axis offset (after affine correction) with Gaussian fit (dashed red line) of data shown in *C*. (*H*) Histogram of *y* axis offset (after affine correction) with Gaussian fit (dashed red line) of data shown in *D*. (*I*) Histogram of *x* axis offset (after piecewise affine correction) with Gaussian fit (dashed red line) of data shown in *E*. (*J*) Histogram of *y* axis offset (after piecewise affine correction) with Gaussian fit (dashed red line) of data shown in *F*. (*G*–*J*) Comparison of the width and the mean of the offset distributions along the *x* and *y* axes for affine and piecewise affine corrected data shows that the additional piecewise correction reduces the width and more importantly results in a mean close to 0.0 nm and thus a very accurate registration. (*K*) *x* axis *μ*_*x*_ and *y* axis *μ*_*y*_ component of registration error for 25 independent image registrations. (*L*) Same data as in *K*, but registration accuracy *σ*_*reg*_ (TRE) is shown for each of the 25 datasets. We accepted datasets for distance determination if *σ*_*reg*_ < 1 nm (blue line cutoff). One frame per TetraSpeck bead was acquired. Details of fitting parameters are provided in *SI Appendix*, Table S4.

As multicolor fiducial markers, we imaged TetraSpeck beads and used a registration function to correct for the offset between color positions (Fig. 2*A*). While previously described registration methods either use a second-degree polynomial fit (16) or linear mapping functions (7) to calculate a registration map, we used a two-step affine-based registration procedure (9) commonly employed in other fields (9) but to our knowledge not previously used to align multicolor single-molecule images. To this end, we first performed a global affine transformation to bring single spots (imaged on two different cameras) in proximity for automated pair assignment (Fig. 2*B*). Next, we applied a piecewise affine transformation, correcting spot positions locally (as detailed below) only using nearby fiducial points (Fig. 2*B* and *SI Appendix*, Fig. S1). In practice, we always acquired three datasets; the first was TetraSpeck beads, the second was the sample of interest, and the third was another acquisition of the TetraSpeck beads (*SI Appendix*, Fig. S1). With the corrected second fiducial marker dataset, we then calculated the target registration error (TRE) (*Materials and Methods* and *SI Appendix*, Fig. S2), determining the deviation between the markers’ *x* and *y* positions in the two channels after alignment (Fig. 2 *C*–*J*). Their mean *μ*_*x*_ and *μ*_*y*_ are the registration error along the *x* axis and *y* axis, respectively. The registration error *σ*_*reg*_ is given by

$$\sigma_{\mathrm{reg}}=\sqrt{{\mu_{x}}^{2}+{\mu_{y}}^{2}.}$$ [1]

Only those samples for which *σ*_*reg*_ was <1 nm were analyzed.

To find the optimal parameter space for image registration, we varied settings for the local piecewise affine transformation as described in more detail in *Materials and Methods*. A minimum of 10 and maximum of 100 fiducial points and a maximum distance of 2 μm resulted in optimal channel registration (*SI Appendix*, Figs. S3 and S4) when a sufficient number of fiducial markers was acquired. This is ∼10,000 fiducial markers for an 80- × 80-μm image (*SI Appendix*, Fig. S1). To obtain this number, ∼400 images with ∼25 beads per field of view were collected.

Using this approach (*Materials and Methods* and *SI Appendix*, *Supplementary Information Protocol*), we routinely (76%) achieved registration accuracy *σ*_*reg*_ of <1 nm (Fig. 2 *K* and *L*). When registration failed (24% of the time), the cause was almost always a slight change in focus during acquisition of the datasets of fiducial markers. Thus, successful execution requires stable optical alignment of the two channels for the duration of the experiment (i.e., <1 nm change in ∼5 to 20 min), a high-quality autofocus system (since registration changes with focus), a motorized xy stage, minimal sample movement during image exposure (i.e., <1 nm sample movement for ∼1 s), and imaging of fiducial markers for image registration and sample of interest on the same slide (*SI Appendix*, Fig. S1). To minimize drift effects we waited 3 s after every stage movement before acquiring data at a new position (*Materials and Methods*). We noticed that the precision (σ_x_, σ_y_) for registering TetraSpeck beads (Fig. 2 *G*–*J*) is lower than expected based on their localization errors. We found this to be caused by displacement of the color centers of TetraSpeck beads by a few nanometers, as reported by others (8) (*SI Appendix*, *Supplementary Information Note 1* and Figs. S5–S7). Taken together, piecewise affine alignment enables image registration at subnanometer accuracy over the entire field of view.

### Measuring Distances of Uniform Samples.

**Fig. 3.**
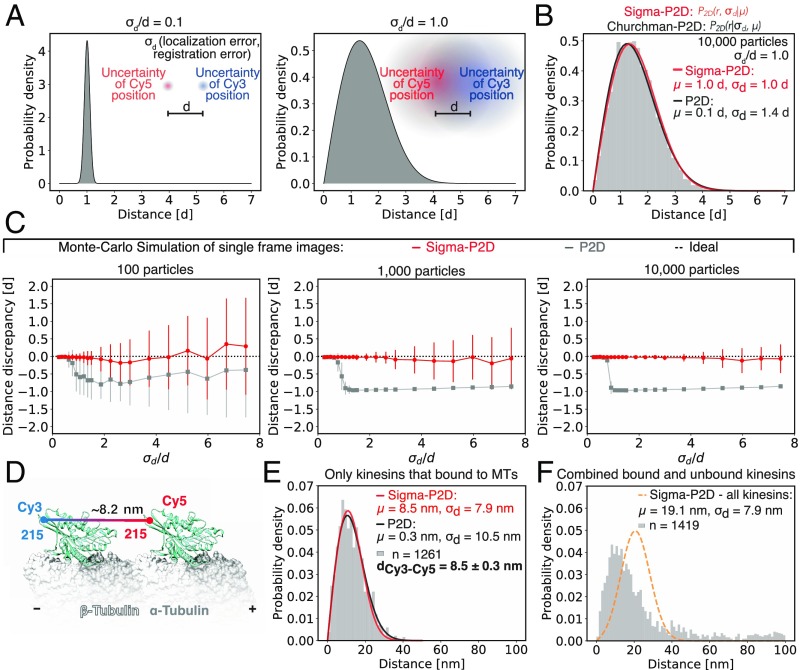
Sigma-P2D: Measuring distances of uniform samples with nanometer accuracy. (*A*) Probability distributions of measured distances between two differently colored fluorophores separated by a true distance *d* for different ratios of uncertainty *σ*_*d*_ over distance *d*. For example, a distance uncertainty of 1 nm and a true distance of 10 nm would generate data as shown on the left while a distance uncertainty of 10 nm and a true distance of 10 nm would generate data as shown on the right. (*B*) Histogram of Monte Carlo-simulated data with a true distance *d* of 1 and distance uncertainty *σ*_*d*_ of 1 fitted with Sigma-P2D (red) and P2D (black). (*C*) Performance of distance prediction by Sigma-P2D (red) and P2D (gray) evaluated using the distance discrepancy (calculated by subtracting the expected distance from the measured distance and normalizing with the expected distance) of Monte Carlo-simulated data. Here, the average distance discrepancy from the true distance was calculated using 100 simulations for different ratios of uncertainty *σ*_*d*_ over distance *d* for 100, 1,000, and 10,000 particles. Error bars show the SD of 100 independent simulations. Distance discrepancies around −1.0 represent cases where the measured distance was 0 nm and the small error bars show that this was very reproducible. This is an example of a precise yet highly inaccurate measurement. Large error bars typically indicate bimodal cases for which we measured both distances that are much larger and distances that are much smaller than the expected distance. (*D*) Diagram of two-head-bound kinesin on a microtubule based on crystal structure (PDB ID code 4LNU) (52) created with UCSF Chimera (53). Positions of Cy3 and Cy5 dye are shown as blue and red dots, respectively. (*E*) Histogram of head-to-head distance measurements of rigor-bound kinesin fitted with Sigma-P2D (red) and P2D (black). The SD of the head-to-head distance with Sigma-P2D fit (bold font, d_Cy3-Cy5_) was calculated by evaluating the Fisher Information matrix. (*F*) Histogram of head-to-head distance measurements of all kinesins (microtubule bound and unbound) fitted with Sigma-P2D (orange dashed line). Single-molecule distances in *E* and *F* were obtained by selecting time-lapse series of individual molecules (*SI Appendix*, Table S6). Details about fitting parameters are listed in *SI Appendix*, Table S4.

Next, we set out to optimize the accuracy and throughput of direct distance determination. Previously, Churchman et al. (3, 6) showed that distances on the scale of the localization error are non-Gaussian distributed (Fig. 3*A* and *SI Appendix*, Fig. S8) and described by the following 2D probability distribution (P2D) (6):

$$p_{2D}\left(r|\mu ,\sigma_{d}\right)=\left(\frac{r}{{\sigma_{d}}^{2}}\right)\exp\left(-\frac{\mu^{2}+r^{2}}{2{\sigma_{d}}^{2}}\right)I_{0}\left(\frac{r\mu }{{\sigma_{d}}^{2}}\right),$$ [2]

in which *r* is the measured Euclidean distance of individual particles, *μ* the estimated average distance, *σ*_*d*_ the distance uncertainty, and *I*_0_ the modified Bessel function of integer order zero. We refer to the true sample distance as “*d*.” Churchman et al. (3, 6) fit this distribution (P2D, Eq. **2**) to Euclidean distance data by means of a maximum likelihood estimation (MLE) with two parameters (*μ* and *σ*_*d*_). We refer to this method simply as 2D probability distribution P2D. However, using both experimental data and Monte Carlo simulations, we found that in case of small changes in distance uncertainty *σ*_*d*_, P2D yields large changes in the estimated distance μ (*SI Appendix*, Fig. S8*B*). An approximation for *σ*_*d*_ ≥ *μ* shows that the probability distribution (P2D, Eq. **2**) becomes independent of distance *μ* (*SI Appendix*, *Supplementary Information Note 2*), resulting in a fit that is driven by the distance uncertainty *σ*_*d*_. Thus, distance estimations of P2D are error-prone for cases where the distance is smaller or of similar size as the distance uncertainty, which is very common for distance measurements in the range of 2 to 30 nm.

To overcome this inaccuracy of the P2D method, we decided to fit the distance distribution with only one parameter, the distance *μ*, and to determine the distance uncertainty *σ*_*d*_ experimentally [$p_{2D}\left(r,\sigma_{d}|\mu \right)$]. This is possible because all parameters of the distance uncertainty *σ*_*d*_ can be measured as it is given by

$$\sigma_{d}=\sqrt{{\sigma_{\mathrm{reg}}}^{2}+{\sigma_{\mathrm{loc}1}}^{2}+{\sigma_{\mathrm{loc}2}}^{2}},$$ [3]

in which *σ*_*loc*1_ and *σ*_*loc*2_ are the localization errors of single particles of fluorophores 1 and 2, respectively, and *σ*_*reg*_ the registration error (*SI Appendix*, *Supplementary Information Note 3*). Thus, by using additional information from the images, we can fit the data only with the important parameter, the distance *μ*, and avoid overfitting. We named this method “Sigma-P2D” (*Materials and Methods* and *SI Appendix*, *Supplementary Information Protocol*). Applying Sigma-P2D to Monte Carlo-simulated data, for which P2D predicted an incorrect distance, we now recovered the true distance with subnanometer accuracy (Fig. 3*B*).

Given that our method can refine measurements made over all distances for which a distance uncertainty can be determined (e.g., fewer than two to hundreds of nanometers), we compared Sigma-P2D and P2D first using Monte Carlo simulations (*Materials and Methods*). We generated model datasets for different ratios of distance uncertainty to distance (*σ*_*d*_/*d*) and evaluated the performance of Sigma-P2D and P2D by calculating the difference between true and estimated distance, normalized by the true distance (distance discrepancy) (Fig. 3*C* and *Materials and Methods*). We found that Sigma-P2D outperforms P2D, especially if *σ*_*d*_ ≥ *μ*, and that even if only 100 particles were used Sigma-P2D estimates the true distance with an offset of less than 20% for almost all ratios of distance uncertainty to distance (Fig. 3*C*). We note that even though the average distance discrepancy might appear small (as for the case with 100 particles) the performance on a single dataset can be poor because large error bars indicate bimodal cases for which we measured both distances that are much larger and distances that are much smaller than the expected distance. However, the accuracy and reproducibility of Sigma-P2D can further be improved by quantifying more particles (Fig. 3*C*) to accuracies of better than 1% of the true distance, while P2D reproducibly (small error bars) underestimates the distance for most conditions by almost 100%. This is an example of a precise and reproducible yet highly inaccurate measurement. Taken together, by incorporating available knowledge of localization and registration errors we greatly improved the fitting routine and can determine distances with subnanometer accuracy and precision.

To evaluate Sigma-P2D experimentally (*SI Appendix*, *Supplementary Information Note 3* and Figs. S9–S11), we imaged a kinesin-1 homodimer for which both heads were rigor-bound with the nonhydrolyzable nucleotide analog AMPPNP to adjacent tubulin dimers along a microtubule protofilament (17) (Fig. 3*D*). Based on electron microscopy (EM) data (18), the distance between the two motor domains is 8.2 nm (the tubulin dimer spacing). A kinesin motor domain construct (17, 19) with a single cysteine residue (E215C) was reacted with an equimolar mixture of maleimide-Cy3 and maleimide-Cy5. Motors that contained both Cy3 and Cy5 and that bound to a biotin–streptavidin-immobilized and Alexa 488-labeled microtubule were selected for two-color distance measurements.

When fitting the data for the apparent head-to-head distance of the rigor-bound kinesins with Sigma-P2D, we measured 8.5 ± 0.3 nm (Fig. 3*E*), which is very close to the expected distance of 8.2 nm. Fitting the same data with the P2D method shows that P2D dramatically underestimated the distance and finds 0.3 ± 1.0 nm (Fig. 3*E*). Unbound kinesins had variable distances, causing the probability distribution fits to yield incorrect results (Fig. 3*F*) since Sigma-P2D does not consider conformational heterogeneity. Hence, Sigma-P2D can only fit samples that are uniform in distance unless prior knowledge about the conformational heterogeneity σ_con_ is available. Nevertheless, utilizing Sigma-P2D we measured the head-to-head distance of a kinesin dimer with subnanometer accuracy and precision.

### Measuring Average Distances of Heterogeneous Samples.

**Fig. 4.**
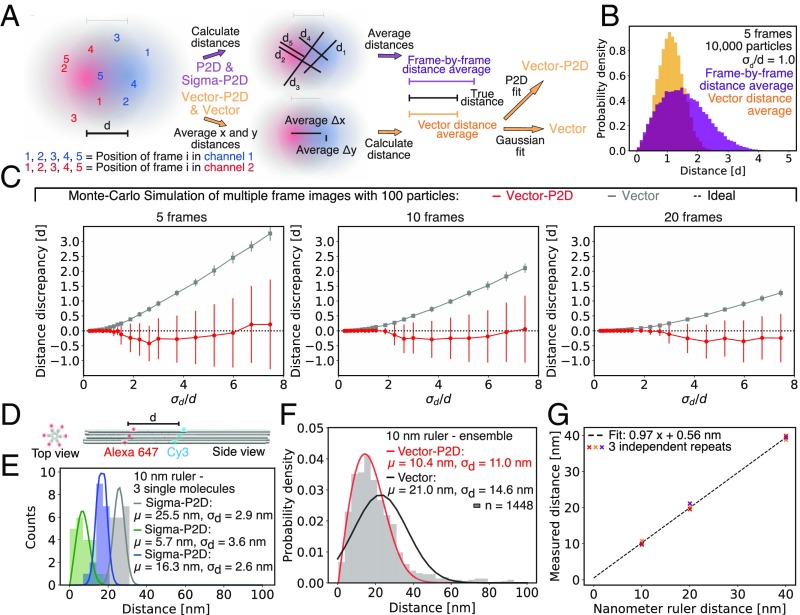
Vector-P2: Measuring distances of variable samples with nanometer accuracy. (*A*) Determining vector averaged distances from data with multiple observations per particle. Intensity distributions for two fluorescent molecules in a red and a blue channel at a true distance *d* of 1. Five independent observations of both molecules were obtained by Monte Carlo simulations (red and blue colored numbers 1–5). Now, either the individual distances of spot pairs can be calculated first and then averaged (frame-by-frame distance average) or average distances along the *x* axis and *y* axis can be determined first and then used to calculate the absolute distance (vector-averaged distances). The vector-averaged distance distribution can then be fit with a Gaussian distribution or the 2D probability distribution “P2D” as shown in Eq. **2**, which use the calculated distance *μ* and the distance uncertainty *σ*_*d*_ as parameters, to yield Vector or Vector-P2D, respectively. (*B*) Histograms for distances generated by means of Monte Carlo simulation with five frames (observations) per particle. Purple histogram shows the distance distribution for a frame-by-frame distance average and orange histogram shows distribution for vector-averaged distances. (*C*) Performance of distance prediction by Vector-P2D (red) and Vector (gray) evaluated using the distance discrepancy (calculated by subtracting the expected distance from the measured distance and normalizing with the expected distance) of Monte Carlo-simulated data. Here, the average discrepancy from the true distance was calculated using 100 simulations for different ratios of uncertainty *σ*_*d*_ over distance *d* for 5, 10, and 20 frames. Error bars show SDs of 100 independent simulations. Large error bars typically indicate bimodal cases for which we measured both distances that are similar to the expected distance and distances that are much smaller than the expected distance. Hence, the increasing size of error bars with increasing *σ*_*d*_/*d* ratios shows that the fitting outcome is becoming more bimodal until it collapses to one side (measuring distances of around 0 nm). Additional data in *SI Appendix*, Fig. S12. (*D*) Design of DNA-origami-based nanorulers for which the “center of mass” between 6 to 10 dyes for each of the two colors determines the distance. (*E*) Histogram of distance distribution of three different single molecules of the 10-nm DNA-origami nanoruler (green, blue, and gray). Solid line is a Sigma-P2D fit. (*F*) Histogram of vector-averaged distance measurements of multiple 10-nm DNA-origami nanorulers analyzed with Vector-P2D (red) and Vector (black). (*G*) Correlation between measured and expected average distance for 10-, 20-, and 40-nm ruler from three technical repeats. Example fits for 20- and 40-nm rulers are shown in *SI Appendix*, Fig. S14. Fitting parameter details are given in *SI Appendix*, Table S4.

Since distance measurements for heterogeneous samples with Sigma-P2D are inadequate and many proteins and protein complexes are heterogeneous in distance, we needed an additional method. To obtain meaningful population statistics for samples which are heterogeneous in distance, it is important to improve the precision with which the two-color distances of individual molecules can be measured. To do so, we collected multiple observations (frames) of the same molecule, by time-lapse imaging (Fig. 4*A*). Rather than directly averaging the distance in each frame, observations of the same fluorescent pair in multiple frames are combined by first averaging distances in *x* and *y* separately, and then using these to calculate the Euclidean distance of individual particles (vector distance average). As previously shown (8), this leads to more accurate distance predictions than direct frame-by-frame Euclidean distance averaging (Fig. 4*A* and *SI Appendix*, Fig. S12*A*), because vector averaging helps to reduce the width of the distance distribution significantly. If, for example, 10,000 particles are imaged and five observations per particle are recorded, either all 50,000 distance measurements (frame-by-frame Euclidean distance) or all 10,000 vector-averaged distances can be combined. For the vector-averaged distances, the distribution is narrower (Fig. 4*B*) but still not perfectly Gaussian-distributed. Instead of fitting with a Gaussian probability distribution as done in a previously developed method (8) (here named “Vector”), we noticed that the fit can further be improved using the 2D probability distribution (P2D, Eq. **2**) and two parameters (*μ* and *σ*_*d*_). Moreover, we noticed that MLE fitting often resulted in inaccurate distance determination for experimental data since it is fairly sensitive to outliers (background noise). Therefore, we fit the P2D function by means of nonlinear least squares (NLLSQ), which is more robust to background noise than MLE (*Materials and Methods*). We called this method “Vector-P2D” and found that Vector-P2D outperforms Vector for all conditions tested using Monte Carlo simulations, which was evaluated as described for the comparison of Sigma-P2D and P2D (*Materials and Methods*). Using Vector-P2D (*SI Appendix*, *Supplementary Information Protocol*), 100 particles with 20 observations each are enough to resolve distances within 20% of the true distance (Fig. 4*C*) for ratios of distance uncertainty to distance (*σ*_*d*_/*d*) of less than 3.5. Increasing the number of particles to 1,000 with 20 observations results in fitted distances that diverge less than 5% from the true distance for ratios of distance uncertainty to distance (*σ*_*d*_/*d*) of less than 5 (*SI Appendix*, Fig. S12 *B*–*G*). Since we only used a true distance of 10 nm in our simulations, we further tested if Vector-P2D can also resolve distances between 2 and 500 nm and found an almost perfect agreement between the true and measured distance (*SI Appendix*, Fig. S12 *H* and *I*). To test whether Vector-P2D can determine the average distance of samples that are variable in distance, we ran Monte Carlo simulations at varying degrees of sample heterogeneity σ_con_ (SD of true distances in the population). If, for instance, 20 frames per particle are recorded, we still recovered the correct population average even for cases where σ_con_ is twice as large as the true distance *d*, (*SI Appendix*, Fig. S13). However, the more heterogeneous the sample, the more frames have to be recorded to achieve accurate results (accuracy being defined as a 20% difference between the measured and predicted distance).

To test the performance of Vector-P2D experimentally, we used DNA-origami-based nanorulers (20, 21). The average “center-of-mass” distance between Cy3 and Alexa 647 fluorophore binding sites on these nanorulers is either 10 nm, 20 nm, or 40 nm. Each color has up to 10 binding sites with an expected labeling efficiency of 50 to 80% (Fig. 4*D*). Together with bleaching effects, this results in variable distances of the color centers of the individual rulers (Fig. 4*E* and *SI Appendix*, Fig. S14 *A* and *C*). However, when we analyzed these rulers using Vector-P2D, we found average distances that were within a nanometer of the expected values (Fig. 4*F*), whereas the Vector method predicted distances up to 100% larger (Fig. 4*F* and *SI Appendix*, Fig. S14 *A*–*D*). Plotting the Vector-P2D–measured population distances for all three nanorulers of three repeats over the expected distances and calculating the slope, we found a slope of 0.97, very close to the ideal value of 1.0 (Fig. 4*G* and *SI Appendix*, Fig. S14 *A*–*D*). Summarizing, using multiple observations of the same molecule and by performing a vector distance average we can recover distances of variable samples with nanometer precision and accuracy.

### Measurements of the Dynein Stalk Length in Multiple Nucleotide States.

**Fig. 5.**
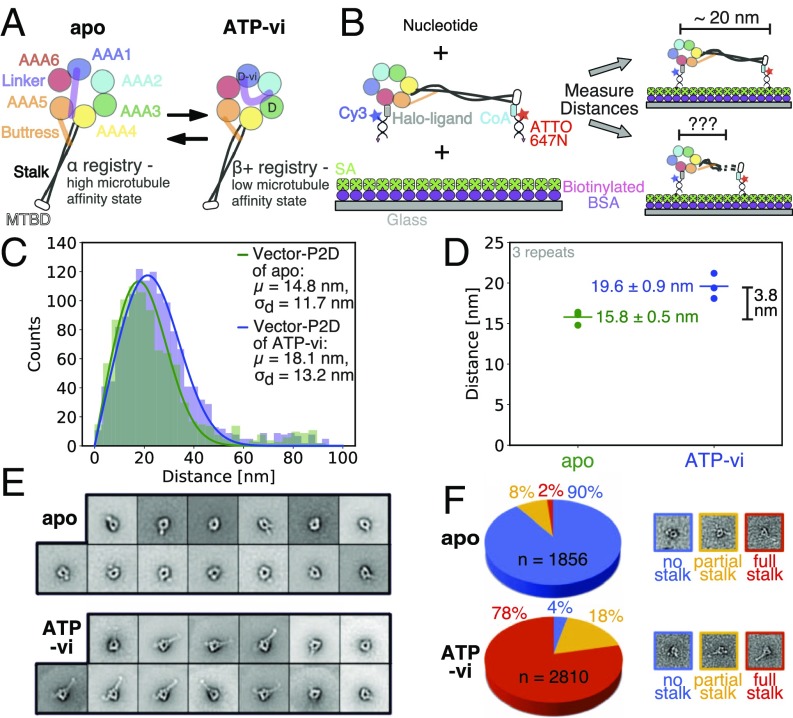
Dynein stalk conformation in two different nucleotide-bound states measured by Vector-P2D and negative-stain EM. (*A*) Schematic of the monomeric dynein motor domain without nucleotide (apo/*Left*) and bound to ATP-vanadate (ATP-vi/*Right*) resulting in a high and low microtubule affinity state, respectively. Transition between both microtubule affinity states happens twice during the hydrolysis cycle: first detachment from microtubule by ATP binding and transition to a low-affinity state and then rebinding to microtubule after ATP hydrolysis and change to a high-affinity state. D indicates ADP in the AAA binding pocket whereas D-vi indicates ADP-vanadate. (*B*) Design for two-color fluorescent distance measurement between AAA ring and MTBD of a dynein monomer. Fluorescent dye, Halo-tag (28), or YBBR-tag (29) ligands and biotin for surface immobilization are attached to a dsDNA oligomer of 16 bp where Cy3 labels the Halo-tag on the C terminus of the AAA ring and ATTO647N is attached to the YBBR-tagged MTBD via the small molecule CoA. The biotin of the dsDNA binds to streptavidin (SA), which is bound to biotinylated BSA. If the stalk is fully extended we expect a distance of about 20 nm (30) between the two colors. (*C*) Histogram of vector-averaged distance measurements of dynein monomer as shown in *B* with apo in green and ATP-vi in blue fitted with Vector-P2D. (*D*) Results of distance measurements of three technical repeats of dynein monomer as shown in *B* with apo in green and ATP-vi in blue. Fitting was done as shown in *C*. (*E*) Negative-stain EM class averages of a dynein monomer in the apo (*Top*) and ATP-vanadate (*Bottom*) state. (*F*) Count and classification of individual particles from negative-stain EM micrographs (as shown in *SI Appendix*, Fig. S15 and Table S1) into three categories (no, partial, and full stalk) for the apo state and the ATP-vi state. Single-molecule distances in *C* and *D* were obtained by selecting time-lapse series of individual molecules (*SI Appendix*, Table S6). Error in *D* is the SEM of three technical repeats. Details about fitting parameters are provided in *SI Appendix*, Table S4.

We next applied our two-color colocalization methods to measure conformational changes in the minus-end-directed, microtubule-based motor dynein (10–12). An intriguing problem for the function of this molecular motor is the two-way communication between the catalytically active AAA ring and the microtubule binding domain (MTBD) through conformational changes in an intervening ∼13-nm antiparallel coiled-coil stalk (13, 22–24) (Fig. 5*A*). Earlier studies have suggested that local melting of the coiled-coil stalk in different states of the nucleotide hydrolysis cycle plays a major role in this communication (25–27), while others have shown that a 4-aa sliding between different registries is critical (13, 25). However, no direct measurements of the distances between the AAA ring and MTBD have been reported, which could help to distinguish between these models.

To tackle this problem, we prepared a yeast cytoplasmic dynein monomer with a C-terminal Halo-tag (28) and a YBBR-tag (29) that was inserted into the MTBD (Fig. 5*B*). Based on crystallographic data, the predicted distance between Halo- and YBBR-tag is ∼20 nm (30) (Fig. 5*B*). To simultaneously immobilize and fluorescently label dynein, both tags were labeled with a 16-bp-long dsDNA that was biotinylated at one end and dye-labeled at the other. We then imaged dynein in the apo and ATP-vanadate (ATP-vi) state and measured the distance between the fluorescent labels using Vector-P2D, since we expected a heterogeneous distance distribution. Using three technical repeats, we measured a distance of 19.6 ± 0.9 nm for the ATP-vanadate state (Fig. 5 *C* and *D*). This is consistent with the X-ray crystallographic studies (30). However, in the apo state (no ATP), we measured a distance of 15.8 ± 0.5 nm between the Halo-tag on the ring and the YBBR-tag in the MTBD. This shorter distance cannot be explained by the “simple helical sliding” model (13, 25), which predicts essentially no distance change.

To further understand the structural basis of our two-color fluorescence measurement, we turned to negative-stain EM. Two-dimensional class averages for the ATP-vanadate bound state show a clear density for the stalk and MTBD in most classes (“full stalk”). In contrast, the stalk density in the apo state was rarely observed (“no stalk”) (Fig. 5*E*). This suggests two possibilities: (*i*) The angle of the stalk differs significantly in the individual molecules in the apo state, leading to these angles’ being averaged out in 2D classes, or (*ii*) the coiled-coil stalk of individual particles in the apo state cannot be identified in the micrographs, suggesting a large-scale conformational change in the stalk. To address these two possibilities, we analyzed the negative-stain data on a single-particle level. Individual particles for multiple nucleotide states were manually scored as belonging to one of three categories: no stalk, partial stalk, and full stalk. Consistent with the results of the class averages, we saw full stalk density for 78% of all particles in the presence of ATP-vanadate and only for 2% in the apo state (Fig. 5*F* and *SI Appendix*, Fig. S15 and Table S1). Moreover, almost all particles (90%) in the apo state do not have any visible density of the stalk, whereas the number of particles for the ATP-vanadate state is a little more distributed among all three categories. This agrees well with our two-color fluorescent distance measurements as the distance distribution in the apo state is narrower than in the ATP-vanadate state. The negative-stain EM data also suggest local melting or conformational changes of the stalk in the apo state. This result is consistent with our two-color fluorescent distance measurements, since disorder (apo state) is expected to reduce the stalk length in comparison with the ordered state (ATP-vanadate state). Together, these single-molecule distance measurements and EM findings suggest that a disorder-to-order transition occurs in the stalk during dynein’s mechanochemical cycle.

## Discussion

Here, we described single-molecule two-color fluorescent microscopy methods that provide nanometer-accuracy distance measurements on the length scale of most macromolecules (2 to 30 nm). Using Monte Carlo simulations and experiments, we show that our techniques enable distance measurements from ∼2 nm to hundreds of nanometers (*SI Appendix*, Fig. S12 *H* and *I*) and can operate with heterogeneous samples. Thus, our methods fill a resolution gap from 8 nm (upper distance of smFRET) to 25 nm (lower bound of current single-molecule colocalization methods). Applying our methods to the molecular motor dynein, we found that the dynein stalk likely undergoes large conformational changes in different nucleotide states.

### Distance Calculations with Nanometer Accuracy.

While smFRET can accurately determine distances in a high-throughput fashion, it is limited to distances that are <8 nm (1, 2). Furthermore, absolute distance measurements are difficult because smFRET is sensitive to fluorophore orientation, which is often assumed to be randomly oriented but nontrivial to measure. There are some existing single-molecule colocalization methods that can be used at the 8- to 25-nm range but all of these methods face certain limitations. For instance, SHREC (3, 6) inaccurately determines distances for cases where distance uncertainty and distance are of similar size. We overcame this limitation by using additional experimental information from the images (Sigma-P2D). A method developed by Pertsinidis et al. (7) also achieves nanometer resolution but is limited to single-pixel measurements and requires highly specialized optical setups, whereas our methods work on the entire field of view of a standard TIRF microscope. Finally, a method by Mortensen et al. (8) resolves nanometer distances with lower resolution (Vector method) and only measures tens of molecules, whereas our methods can measure up to 10,000 molecules in a single experiment.

In general, we significantly improved and extended existing methods by using additional experimental information (Sigma-P2D) and by improving analysis techniques of multiple observations of the same particle (8) (Vector-P2D). Whether Sigma-P2D or Vector-P2D performs better depends on the experimental conditions, such as distance uniformity of the molecules, whether or not multiple frames can be acquired, and whether distances of an individual single molecule or populations of single molecules are desired. Our Sigma-P2D approach only recovers the distance from a collection of uniform particles and is useful to determine whether or not a sample is uniform in distance (*SI Appendix*, Fig. S16). The Vector-P2D method can measure the average distance of both, samples that are uniform and variable in distance. However, Sigma-P2D works better for samples that are uniform in distance because it can recover distances even for extremely high ratios of distance uncertainty to distance (*σ*_*d*_/*d*). In addition, Vector-P2D requires more than one frame per particle to determine the vector average distance while Sigma-P2D also works for single-frame data. In *SI Appendix*, Fig. S16 we provide detailed guidelines to help choose between Sigma-P2D and Vector-P2D.

If only a single molecule and not a population is of interest, applicable methods are Sigma-P2D and Vector (Vector and Vector-P2D are equivalent under this condition since only one data point can be fitted with the P2D function after vector averaging). Comparing both using Monte Carlo-simulated data, we found that Sigma-P2D performs better than Vector for almost all conditions when distance distributions of single particles are analyzed (*SI Appendix*, Fig. S17). Thus, for distance analysis of an individual single molecule, Sigma-P2D is the method of choice.

Like other existing colocalization-based two-color distance measurement methods (7, 8), our methods require surface immobilization of the sample and are limited to projections in two dimensions. Nevertheless, using versatile labeling techniques (such as the DNA-based surface coupling combined with labeling as we used for the dynein experiment), we believe that there are many ways to obtain useful information—difficult or impossible to acquire otherwise—while being aware of this limitation. A high-quality autofocus system is essential for these two-color distance measurements, since the image registration changes with focus. Thus, imaging of fiducial markers for image registration and sample of interest on the same slide (*SI Appendix*, Fig. S1) is necessary. Restricted dye mobility causes changes in the point-spread function, leading to systematic localization errors (7, 31) and incorrect distance measurements. We observed a “normal” point-spread function shape in all our samples and also used intensity comparisons between linearly and circularly polarized light to ascertain full dye mobility. In summary, our methods, Sigma-P2D and Vector-P2D, together with the piecewise affine image registration and the μManager plugin (14), allow distance measurements in less than 2 h on a standard TIRF microscope, enabling high-throughput distance measurements with nanometer accuracy.

### Stalk of Dynein Likely Undergoes Large Conformational Changes.

In order for dynein to step along microtubules, the hydrolysis state of the nucleotide-binding AAA ring is coupled to microtubule affinity of the MTBD through the stalk (13, 22–24). Several studies suggest that local melting of the coiled-coil in different states of the nucleotide hydrolysis cycle plays a major role in this communication (25–27), while others have shown that sliding between different registries is essential (13, 25). However, no direct measurements of the distances between the AAA ring and MTBD have been reported. Using the Vector-P2D method, we measured this distance directly in different nucleotide states and found evidence for a large conformational change in the dynein stalk. These measurements would not have been possible with other methods such as smFRET, since we could not have placed any fluorescent labels in the working range of smFRET (2 to 8 nm) as the stalk of dynein is 13 nm long. Moreover, the negative-stain EM approach also did not allow direct distance measurements, since one of the conformational states was not visible, presumably due to disorder.

Our observations do not rule out registry sliding of the stalk (13, 25); however, the changes in distance cannot be explained by simple sliding and small conformational rearrangements alone. Rather, our evidence is consistent with a local “melting” of the stalk (25–27). Based on the distance measured in the apo state, we speculate that some part of the stalk between the MTBD and the buttress/stalk interaction is involved in these conformational changes. This is in good agreement with the model in which a highly conserved tryptophan in the stalk, located close to the buttress contact, melts coiled-coil 1 (32). Such melting could underlie the reduction in the distance between the ring and the MTBD.

### Concluding Remarks.

In summary, we have developed nanometer-accuracy distance measurements for two differently colored fluorophores bound to static proteins. In the future, it will be worthwhile to extend our techniques to perform dynamic measurements of individual molecules. If, for instance, one wants to map the stepping of an individual molecular motor onto the lattice of its track, Sigma-P2D will be particularly useful.

The theoretical concepts and their application to nanometer-distance measurements presented in this work are not limited to two-color fluorescent single-molecule colocalization microscopy but apply to all distance measurements where the distance is similar to the error and thus also to other superresolution imaging techniques (33). As these methods venture into the regime of nanometer resolution (34), we anticipate that our methodology and open-source software will be useful for a broad range of superresolution fluorescence microscopy technologies.

## Materials and Methods

### Flow-Cell Preparation.

We custom-made three-cell flow chambers using laser-cut double-sided adhesive sheets (9474-08x12 - 3M 9474LE 300LSE; Soles2dance), glass slides (12-550-123; Thermo Fisher Scientific), and 170-μm-thick coverslips (474030-9000-000; Zeiss). The coverslips were cleaned in a 5% vol/vol solution of Hellmanex III (Z805939-1EA; Sigma) at 50 °C overnight and washed extensively with Milli-Q water afterward. Flow cells were assembled so that each chamber holds ∼10 µL (*SI Appendix*, Fig. S1).

### Fluorescent Beads for Image Registration.

We used TetraSpeck beads (T7279; Thermo Fisher Scientific) with a diameter of ∼100 nm for image registration. To prepare the beads for imaging we added 10 µL of 1 mg/mL poly-D-lysine (P6407; Sigma) in Milli-Q water to the flow cell and incubated for 3 min, washed with 20 µL of BRB80 [80 mM Pipes (pH 6.8), 1 mM EGTA, and 1 mM MgCl_2_], and then added 10 µL of 1:1,000 diluted TetraSpeck beads in BRB80 and incubated for 5 min. Finally, we washed the flow cell with 40 µL of BRB80.

### Preparation of dsDNA Samples.

For the 30-bp single biotin dsDNA construct we used the following: strand A: /5Cy3/GGGTATGGAGATTTTTAGCGGAGTGACAGC/3Cy5Sp/ and strand B: /5BiosG/AAAAAAAAAAAAGCTGTCACTCCGCTAAAAATCTCCATACCC, both purchased from Integrated DNA Technologies (IDT). The dsDNA constructs were assembled by mixing 10 µM of strands A and B with assembly buffer [20 mM Tris (pH 8.0), 1 mM EDTA, and 2.5 mM MgCl_2_] and heating the mixture to 95 °C for 5 min, followed by cooling down to 20 °C at a rate of 1 °C per minute. For imaging, we diluted the constructs in assembly buffer to 3 pM. Samples for imaging are prepared by adding 10 µL of 5 mg/mL Biotin-BSA (29130; Thermo Fisher Scientific) in BRB80 to the flow cell, incubating for 2 min, adding 10 µL of 5 mg/mL Biotin-BSA in BRB80, incubating for 2 min, washing with 20 µL of BRB80, adding 10 µL of 0.5 mg/mL streptavidin (S888; Thermo Fisher Scientific) in PBS pH 7.4, and incubating for 2 min. We then washed with 20 µL of PBS (pH 7.4), added 10 µL of 3 pM dsDNA construct in PBS (pH 7.4), and incubated for 5 min. Next, we washed with 30 µL of PBS (pH 7.4) and finally added the PCA/PCD/Trolox oxygen scavenging system (35) in PBS (pH 7.4).

### DNA-Origami Standards.

Custom DNA-origami nanorulers (21) were purchased from GATTAquant GmbH. The nanoruler design is based on the 12HB and is externally labeled with fluorescent dye molecules (Cy3 and Alexa 647). The “center of mass” between the Cy3 binding sites and the Alexa 647 binding sites is either 10 nm, 20 nm, or 40 nm. Each color has up to 10 binding sites with an expected labeling efficiency of 50 to 80%. In addition, each nanoruler has multiple biotins attached for immobilization on a coverslip. Samples for imaging are prepared by twice adding 10 µL of 5 mg/mL Biotin-BSA in BRB80 to the flow cell and incubation for 2 min, washing with 20 µL of BRB80, addition of 10 µL of 0.5 mg/mL streptavidin in PBS (pH 7.4), and a 2-min incubation. We then washed with 20 µL of PBS (pH 7.4) supplemented with 10 mM MgCl_2_. In a next step 10 µL of DNA-origami ruler was added and incubated for 5 min. Next, we washed with 30 µL of PBS (pH 7.4) supplemented with 10 mM MgCl_2_ and finally added the PCA/PCD/Trolox oxygen scavenging system (35) in PBS (pH 7.4) supplemented with 10 mM MgCl_2_.

### Kinesin Cloning, Purification, and Labeling.

The kinesin construct was cloned and purified as previously described (17, 19). Briefly, cysteine residues were introduced into a “cysteine-light” human kinesin-1 dimer that is 490 aa long (K490). The homodimeric E215C K490 contains a carboxyl-terminal His_6_ tag.

The plasmid was transfected and expressed in Agilent BL21(DE3). Cells were grown in LB at 37 °C until they reached 0.6 OD_280_, expression was induced by addition of 1 mM isopropyl β-d-1-thiogalactopyranoside, and cells were incubated overnight at 18 °C. Cells were pelleted and harvested in lysis buffer [25 mM Pipes (pH 6.8), 2 mM MgCl_2_, 250 mM NaCl, 20 mM imidazole, 2 mM TCEP, and 5% sucrose] and lysed in the Avestin Emulsiflex homogenizer (ATA Scientific) in the presence of protease inhibitors. After a spin in a Sorvall SS-34 rotor for 30 min at 30,000 × *g*, the supernatant was loaded onto a Ni-NTA resin (30210; Qiagen) and washed with additional lysis buffer. Then, the protein was eluted by adding 300 mM of imidazole to the lysis buffer. The elutions were dialyzed overnight against dialysis buffer [25 mM Pipes (pH 6.8), 2 mM MgCl_2_, 200 mM NaCl, 1 mM EGTA, 2 mM TCEP, and 10% sucrose].

Afterward, the E215C K490 was reacted for 4 h at 4 °C with Cy3-maleimide (PA13131; GE Healthcare) and Cy5-maleimide (PA15131; GE Healthcare) at a motor/Cy3 dye/Cy5 dye ratio of 1:10:10. Unreacted maleimide dyes were quenched by the addition of 1 mM DTT. Subsequently the sample was purified by gel filtration over a S200 10/300GL column from GE Healthcare. Gel filtration buffer was composed of 25 mM Pipes (pH 6.8), 2 mM MgCl_2_, 200 mM NaCl, 1 mM EGTA, 1 mM DTT, and 10% sucrose. Finally, the sample was flash-frozen and stored at −80 °C.

### Dynein Cloning, Purification, and Labeling.

Dynein was expressed and purified as previously described (36). Monomeric constructs for negative-stain imaging were further purified by gel filtration on a GE Healthcare Superdex 200 10/300GL in dynein gel filtration buffer [20 mM Tris (pH 8.0), 50 mM K-Ac, 2 mM Mg(Ac)_2_, 1 mM EGTA, 1 mM TCEP, and 10% glycerol] and flash-frozen afterward. For the negative-stain images we used the VY137 construct with the following genotype: PGal:ZZ:Tev:GFP:HA:D6 MATa; his3-11,15; ura3-1; leu2-3,112; ade2-1; trp1-1; PEP4::HIS5; PRB1D. For the in-solution distance measurements we added a c-terminal Halo-tag (28) and inserted a YBBR-tag (29) into the MTBD - VY1067. Before gel filtration, the monomer was labeled on ice overnight with two 16-bp-long dsDNA constructs (D–E and F–G) that were dimerized a priori with assembly buffer [20 mM Tris (pH 8.0), 1 mM EDTA, and 2.5 mM MgCl_2_] and heating the mixture to 95 °C for 5 min, followed by a cooling of 1 °C per minute down to 20 °C. The Halo-tag and YBBR-tag labeling was carried out as previously described (37). Briefly, we mixed 10 mM MgCl_2_, 2.5 μM Sfp phosphopantetheinyl transferase, 10 μM DNA–Halo, 20 μM DNA–CoA, and 500 nM ybbR-tagged dynein (all final concentrations). Afterward, we removed excess DNA strands by gel filtration on a GE Healthcare Superdex 200 10/300GL in dynein gel filtration buffer and then flash-froze the sample. The oligos were ordered from Biomers GmbH and IDT with the following sequences and modifications: strand D: /CoA/AGGATGAGTGAGAGTG (Biomers); strand E: /5BiosG/CACTCTCACTCATCCTT/3Cy3Sp/ (IDT); strand F: /HALO/AGGATGAGTGAGAGTG (Biomers); and strand G: /5BiosG/CACTCTCACTCATCCTT/3ATTO647NN/ (IDT).

### Preparation of Microtubules.

Tubulin was purified and polymerized as previously described (38). Unlabeled tubulin, biotinylated tubulin, and fluorescent tubulin were mixed at a ratio of 50:2:1 in BRB80 and 1 mM GTP was added. Then, the mixture was incubated in a 37 °C water bath for 15 min. Afterward, 20 µM of Taxol (T1912; Sigma) was added and the mixture was incubated for an additional 2 h at 37 °C. Before use, microtubules were spun over a 25% sucrose cushion in BRB80 at ∼160,000 × *g* for 10 min in a tabletop centrifuge. Afterward, the pellet was resuspended in BRB80 with 10 µM of Taxol.

### Preparation of Flow Cells with Kinesin.

Flow cells with immobilized kinesin were prepared as previously described (17). First, we added 10 µL of 5 mg/mL Biotin-BSA in BRB80 to the flow cell and incubated for 2 min. Then, we again added 10 µL of 5 mg/mL Biotin-BSA in BRB80 and incubated for 2 min. Afterward, we washed with 20 µL of BRB80 with 2 mg/mL β-casein (C6905; Sigma) and 0.4 mg/mL κ-casein (C0406; Sigma). This was followed by addition of 10 µL of 0.5 mg/mL streptavidin in PBS (pH 7.4) and a 2-min incubation. We then washed with 20 µL of BRB80 with 2 mg/mL β-casein and 0.4 mg/mL κ-casein. In a next step 10 µL of polymerized, biotinylated, Alexa 488-labeled microtubules were added and incubated for 5 min. Next, we washed with 30 µL of BRB80 with 2 mg/mL β-casein, 0.4 mg/mL κ-casein, and 10 µM Taxol. Then, we added 10 µL of K490 in BRB80 with 2 mg/mL β-casein, 0.4 mg/mL κ-casein, 10 µM Taxol, and 1 mM AMPPNP (10102547001; Sigma) and incubated for 5 min. Afterward, we washed with 30 µL of BRB80 with 1 mg/mL β-casein, 0.2 mg/mL κ-casein, 10 µM Taxol, and 1 mM AMPPNP. Finally, we added the PCA/PCD/Trolox oxygen scavenging system (35) in BRB80 with 1 mg/mL β-casein, 0.2 mg/mL κ-casein, 10 µM Taxol, and 1 mM AMPPNP.

### Preparation of Flow Cells with Dynein.

The flow cells for the distance measurements between the AAA ring and the MTBD of dynein were prepared as follows. First, we mixed DNA-labeled, monomeric dynein in DAB [30 mM Hepes (pH 7.4), 50 mM K-Ac, 2 mM Mg(Ac)_2_, 1 mM EGTA, and 1 mM TCEP] with 1 mM Mg-ATP and 1 mM vanadate (450243; Sigma) and incubated at room temperature for 15 min. We also prepared a dynein dilution in DAB for the apo state and also incubated it at room temperature for 15 min. In the meantime, we prepared two identical flow cells for the apo and ATP vanadate state on the same microscopy slide. Therefore, we added 10 µL of 5 mg/mL Biotin-BSA in BRB80 twice and incubated for 2 min each time. Afterward the flow cell was washed with 20 µL of BRB80 with 2 mg/mL β-casein (C6905; Sigma) and 0.4 mg/mL κ-casein (C0406; Sigma). We then added 10 µL of 0.5 mg/mL streptavidin in PBS (pH 7.4) and incubated for another 2 min. This was followed by a wash with 20 µL DAB with 2 mg/mL β-casein (C6905; Sigma) and 0.4 mg/mL κ-casein (C0406; Sigma). Next, we incubated with 10 µL of either dynein solution, apo and ATP vanadate, for 5 min. Afterward, we washed with 30 µL of DAB with 1 mg/mL β-casein and 0.2 mg/mL κ-casein. For the ATP vanadate state we added 1 mM of Mg-ATP and 1 mM of vanadate. Finally, we added 10 µL of the PCA/PCD/Trolox oxygen scavenging system (35) in DAB with 1 mg/mL β-casein and 0.2 mg/mL κ-casein. For the ATP vanadate state the buffer was supplemented with 1 mM Mg-ATP and 1 mM vanadate.

### Microscope Setup.

Data collection was performed at room temperature (∼23 °C) using through-the-objective TIRF inverted microscopy on a Nikon Eclipse Ti microscope equipped with a 100× (1.45 N.A.) oil objective (Plan Apo ƛ; Nikon). We used two stepping motor actuators (SGSP-25ACTR-B0; Sigma Koki) mounted on a KS stage (model KS-N) and a custom-built cover to reduce noise from air and temperature fluctuations. A reflection-based autofocus unit (Focustat4) was custom-adapted to our microscope (Focal Point Inc.). We applied Nikon Type NF2 immersion oil (MXA22126; Nikon) to all slides. Three laser lines at 488 nm (Coherent Sapphire 488 LP, 150 mW), 561 nm (Coherent Sapphire 561 LP, 150 mW), and 640 nm (Coherent CUBE 640-100C, 100 mW) were guided through an AOTF (48062-XX-0.55; Neos), enlarged sixfold, passed through a quarter-wave plate (AQWP05M-600; ThorLabs), and focused using an achromatic doublet f = 100 mm on a conjugate back focal plane of the objective outside of the microscope. The TIRF angle was adjusted by moving a mirror and focusing lens simultaneously. A TIRF cube containing excitation filter (zet405/491/561/638x; Chroma), dichroic mirror (zt405/488/561/638rpc), and emission filter ( zet405/491/561/647m; Chroma) was mounted in the upper turret of the microscope. The lower turret contained a filter cube (TE/Ti2000_Mounted, ET605/70m, T660lpxr, ET700/75m; Chroma) that directs Cy3 emission toward the back camera and the Cy5 emission toward the left camera. We used two Andor iXon 512 × 512 EM cameras, DU-897E. The acquisition software was μManager (14) 2.0. All acquisitions were carried out with alternating excitation between the 561 and 640 laser lines (to avoid considerable background fluorescence in the Cy5 channel caused by 561-nm laser excitation). Image pixel size was 159 nm.

### Single-Molecule TIRF Data Collection.

For TetraSpeck bead acquisitions an exposure time of 100 ms and for all other samples 400 ms was used, if not otherwise specified. After every stage movement for data acquisition at a new position we waited 3 s before collecting data to minimize drift effects, because we noticed large stage drift right after every stage movement, which was significantly lower a couple of seconds after stage movement. We used the cameras in conventional CCD mode (i.e., no EM gain). All datasets were acquired with a “16 bit, conventional, 3 MHz” setting and a preamp gain of 5×. More details of image acquisition settings and laser power settings for each individual dataset are shown in *SI Appendix*, Tables S4–S6. A step-by-step protocol for data acquisition is given in *SI Appendix*, *Supplementary Information Protocol*.

### Negative-Stain EM Data Collection and Processing.

Nucleotide-bound samples were prepared with 5 mM ATP + sodium vanadate in addition to equimolar magnesium acetate. For negative-stain EM, samples were applied to freshly glow-discharged carbon-coated 400-mesh copper grids and blotted off. Immediately after blotting, a 0.75% uranyl formate solution was applied for staining and blotted off. The stain was applied five times per sample. Samples were allowed to air-dry before imaging. Data were acquired at University of California, San Francisco on a Tecnai T12 microscope operating at 120 kV, using a 4k × 4k CCD camera (UltraScan 4000; Gatan) and a pixel size of 2.1 Å per pixel. Particles were picked and boxed using scripts from SAMUEL and SamViewer (https://liao.hms.harvard.edu/samuel). Two-dimensional classification was used to clean our stack and obtain a set of good particles. Only top views (views in which the AAA ring could be clearly identified) were used. Particles were manually scored as having a full stalk (MTBD visible), partial stalk (stalk is visible but MTBD is not) or no stalk (stalk cannot be identified in the micrograph) (*SI Appendix*, Table S1). For an unbiased sorting, we randomly assigned unique identifiers (10-digit number) to each particle in the apo and ATP-vanadate state, pooled all particles from both nucleotide states, sorted them manually into the three different classes (stalk, partial stalk, or no stalk), and then decoded particles based on the unique identifier to sort the particle back into the apo or ATP-vanadate states.

### Single-Molecule Localization.

All emitters were fitted and localized using μManager’s (14) “localization microscopy” plug-in. For emitter fitting we implemented a Gaussian-based maximum-likelihood estimation (15) in μManager (14) and used the following starting conditions. The *x* and *y* position were determined by centroid calculation, the width was set to 0.9 pixels, background was calculated by summing the intensities of all outermost pixels of a region of interest, and intensity was determined by summing up all intensities within the region of interest minus the background value. After fitting, intensities and backgrounds were converted to photon count by applying the photon conversion factor and correcting for camera offset and read noise. Width and *x*, *y* coordinates were then converted from pixel to nanometer space (1 pixel = 159 nm). When fitting emitters with μManager’s (14) localization microscopy plug-in a noise tolerance and box size can be set. Parameters for analysis are shown in *SI Appendix*, Table S4.

We then calculated the variance in fluorophore localization using the MLEwG method (15). Note that we used intensity and background values determined by the aperture method (39) and not values determined by the MLE emitter fitting because the aperture method values agreed better with the experimentally measured variance (*SI Appendix*, Fig. S11). A step-by-step protocol for single-molecule localization is given in *SI Appendix*, *Supplementary Information Protocol*.

### Image Registration.

For image registration two datasets were always acquired: fiducial markers (TetraSpeck beads) to determine the registration map before imaging the sample of interest and a second set of beads to test the stability of the registration (TRE) after the sample of interest. To ensure high quality of the registration map during the experiment, we determined the TRE, which reports the distance (ideally 0.0 nm) for fiducials other than the points used to create the registration map (40) and which is more critical than the fiducial registration error (*SI Appendix*, Fig. S2). Registrations were carried out by first applying a global affine transformation (determined from the bead images) to bring the coordinates in the two channels in close-enough proximity for automated pair assignment (*SI Appendix*, Fig. S1). Final registration was accomplished by applying a second affine transform constructed from beads in the immediate vicinity of each pair (i.e., each pair has its own piecewise affine transform). This piecewise affine transformation (9) was also used to calculate the TRE from the second set of bead images by determining the difference in *x* and *y* position of each bead after registration (Eq. **1**). Since piecewise affine alignment is based on a nearest-neighbor search (9), three parameters can influence registration outcome: minimum and maximum number of fiducial points and the maximum distance to the control point. Higher maximum distance and higher maximum number of points caused distortions, indicating that the registration was not executed properly (*SI Appendix*, Fig. S3). However, when the maximum distance is too small an area in the micrograph may not contain the minimum number of fiducials, and thus will not be corrected (white areas in *SI Appendix*, Fig. S3*C*). Based on the analysis of many different parameter combinations (*SI Appendix*, Fig. S4), we used the following settings for piecewise affine maps: a minimum of 10 and a maximum of 100 fiducial points as well as a maximum distance of 2 μm (except for *SI Appendix*, Fig. S7, where a maximum distance of 3 μm was used, and *SI Appendix*, Fig. S3, where values are provided in the figure caption). A step-by-step protocol for image registration is given in *SI Appendix*, *Supplementary Information Protocol*.

### Single-Molecule Data Analysis and Distance Determination.

All datasets were analyzed using μManager’s (14) localization microscopy plug-in. The fitting method (P2D, Sigma-P2D, Vector-P2D, and Vector) to calculate the distance between two fluorophores is either indicated in the figure and/or figure legend. To avoid erroneous results caused by floating point under- or overflows during the calculation of P2D (3), intermediate results were tested for such conditions and set to minimum or maximum floating-point number when appropriate. Furthermore, an approximation [appendix B of Churchman et al. (6)] of the P2D function was used,

$$p_{2D}\approx \frac{1}{\sqrt{2\pi }\sigma_{d}}\sqrt{\frac{r}{\mu }}\text{exp}\left(-\frac{{\left(r-\mu \right)}^{2}}{2{\sigma_{d}}^{2}}\right),$$ [4]

when the estimate of *σ*_*d*_ was smaller than half the estimate of the distance.

For P2D and Sigma-P2D the data were fit by means of MLE as described in *Results*. For Vector and Vector-P2D we used a more outlier-robust fitting method (NLLSQ fitting) since experimental data usually contain some background noise causing incorrect fitting results when using MLE for Vector-P2D. We could have also removed outliers from the data but it is not always possible to distinguish “real” data points from outliers and small changes in threshold value (cutoff for measured distances) dramatically influence the outcome of the maximum likelihood fit of distance *μ*. Setting the cutoff for the measured distances too low or too high can dramatically change the value of the estimated distance for MLE fitting. When fitting with NLLSQ, setting the distance cutoff too low might influence the outcome. However, since NLLSQ is less sensitive to outliers, the cutoff can always be set to high values (e.g., four to five times of the expected distances) and therewith erroneous fitting results are less likely.

To overcome problems with bin-size settings for histograms when fitting with NLLSQ we converted the experimental data into an empirical cumulative distribution function and fit this with numeric integration of the P2D. We show by means of Monte Carlo simulation that NLLSQ fitting is as good as MLE for data lacking background noise and that NLLSQ fitting is as good as or better than MLE fitting in all conditions where random background noise up to 5% was added (for ratios of distance uncertainty to distance of up to 2). At higher levels of background noise, both methods fail to recover the true distance (*SI Appendix*, Fig. S18). Overall, we observe that with increasing background noise the NLLSQ fitting becomes more sensitive to higher values of distance uncertainty *σ*_*d*_ (*SI Appendix*, Fig. S18).

An SEM for distance calculations using Sigma-P2D and P2D (Fig. 3) was determined by means of Fisher information matrix whereas bootstrapping was used for Vector-P2D and Vector (Figs. 4 and 5). Parameters for analysis are shown in *SI Appendix*, Tables S4 and S6. A step-by-step protocol for data analysis is given in *SI Appendix*, *Supplementary Information Protocol*.

### Monte Carlo Simulations.

In silico two-color distance measurements by means of Monte Carlo simulation were carried out with a custom Python script. In brief, the true distance *µ*, the two localization errors *σ*_*loc*_1_ and *σ*_*loc*_2_, their underlying distributions [*σ*_*σ*(*loc*_1)_, *σ*_*σ*(*loc*_2)_], sample conformational heterogeneity σ_con_, the number of pairs observed, and the number of frames (observations) per pair can be varied in parallel. The simulation for each parameter combination can be repeated multiple times if desired. For the variance in the fluorophores localization a Gaussian distribution is applied to true positions of channels 1 and 2 and a Gamma distribution is applied as the underlying distribution of the variance in the fluorophores localization for channels 1 and 2. We analyzed model datasets based on different ratios of distance uncertainty to distance (*σ*_*d*_/*μ*). For each ratio we evaluated 100 datasets with Sigma-P2D and P2D or Vector and Vector-P2D and calculated the average distance discrepancy. Therefore, we subtracted the expected distance from the measured distance and normalized by the expected distance. Thus, values around −1.0 represent cases for which we measured 0 nm and for which we find very small error bars showing that this is very reproducible. This is an example of a precise yet highly inaccurate measurement. Large error bars typically indicate bimodal cases for which we measured both distances that are much larger or similar to the expected distances and distances that are much smaller than the expected distance. We defined measurements as reliable when they resulted in an average distance discrepancy of less than 20% from the true distance with a SD of less than 30% of the true distance. Based on common localization errors for single-molecule studies (*SI Appendix*, Fig. S10) and distances on the nanometer scale (∼2 to 30 nm), we expect ratios (*σ*_*d*_/*μ*) of up to 4 to be of experimental relevance. However, we included even higher ratios to probe the upper limits of Sigma-P2D and Vector-P2D. For more details see ref. 41.

### Statistics.

For each result the inherent uncertainty due to random or systematic errors and their validation are discussed in the relevant sections of the paper. Details about the sample size, number of independent calculations, and the determination of error bars in plots are included in the figures and figure legends.

### Code Availability.

The custom-written Python code for Monte Carlo simulations is available under the Berkeley Software Distribution (BSD) license on Zenodo (41). μManager acquisition and analysis software is available partly under the BSD license, partly under the GNU Lesser General Public License on Zenodo (42). Installers for Mac and Windows for Micro-Manager version 2.0-gamma created on February 10, 2019 can be downloaded from Zenodo (43).

### Data Availability.

Raw datasets used in this study and to be used to test the software, to create and test registration maps, and to measure distances are provided on Zenodo (44). In addition, we provide a step-by-step *SI Appendix*, *Supplementary Information Protocol* that describes how this raw data can be analyzed with μManager’s (14) localization microscopy plug-in. The raw negative-stain EM micrographs can be found at Zenodo (44).

## Supplementary Material

Supplementary File
