# Supplementary material for: Nanometer-accuracy distance measurements between fluorophores at the single-molecule level
Source: Proc Natl Acad Sci U S A. 2019 Feb 15;116(10):4275–84. doi: 10.1073/pnas.1815826116 (PMC6410877; doi:10.1073/pnas.1815826116)
Supplement: Supplementary File [file pnas.1815826116.sapp.pdf]

Supplementary Information

for

Nanometer-accuracy distance measurements between fluorophores at the single-molecule level

Stefan Niekamp[1], Jongmin Sung[1], Walter Huynh[1], Gira Bhabha[2], Ronald D. Vale[1]*, and Nico Stuurman[1]

1 Department of Cellular and Molecular Pharmacology and the Howard Hughes Medical Institute, University of California, San Francisco, 600 16th Street, San Francisco, CA 94158.
2 Skirball Institute of Biomolecular Medicine and Department of Cell Biology, New York University School of Medicine, New York, NY 10016

* Corresponding author: ron.vale@ucsf.edu

<u>This document includes:</u>

- Supplementary Information Figures (Fig. S1 - S18)
- Supplementary Information Tables (Table S1 - S6)
- Supplementary Information Notes (1-3)
- Supplementary Information Protocol
- References for the Supplementary Information
- Supplementary Information - Reviewers Comments

www.pnas.org/cgi/doi/10.1073/pnas.1815826116

Supplementary Information Figures

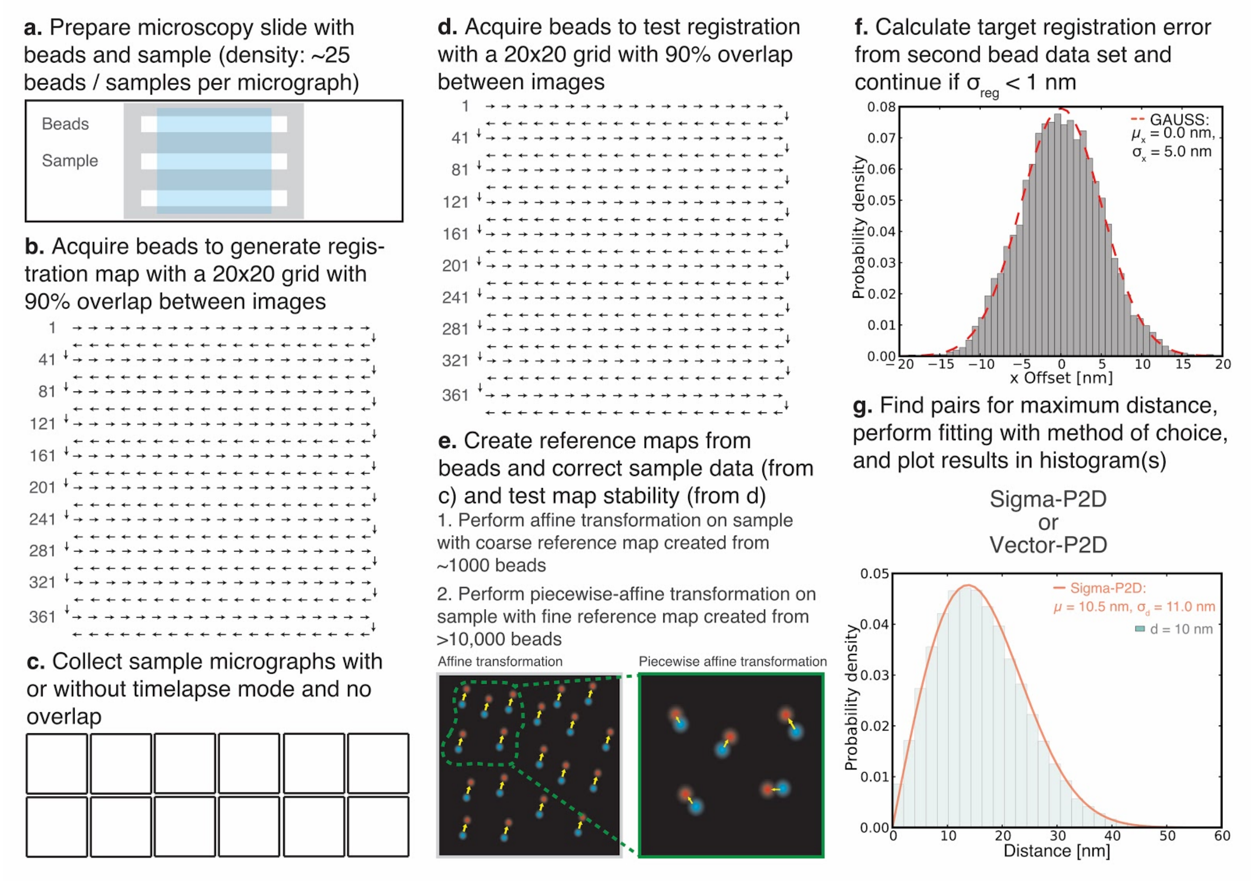

Fig. S1 | Workflow for image registration and distance measurements. (a) Set-up of microscope slide with fiducial markers in one and sample of interest in the another chamber. (b) Image acquisition pattern for fiducial markers to create registration map. (c) Image acquisition pattern for sample of interest. (d) Same as in b but this time to test the registration map after sample data collection. (e) µManager (1) analysis procedure to create affine and piecewise affine registration maps. For more details see SI Protocol. (f) Calculation of image registration accuracy. (g) µManager (1) analysis procedure to determine distance distribution with Sigma-P2D (sample uniform in distance) or Vector-2D (sample heterogeneous / variable in distance). For more details see SI Protocol.

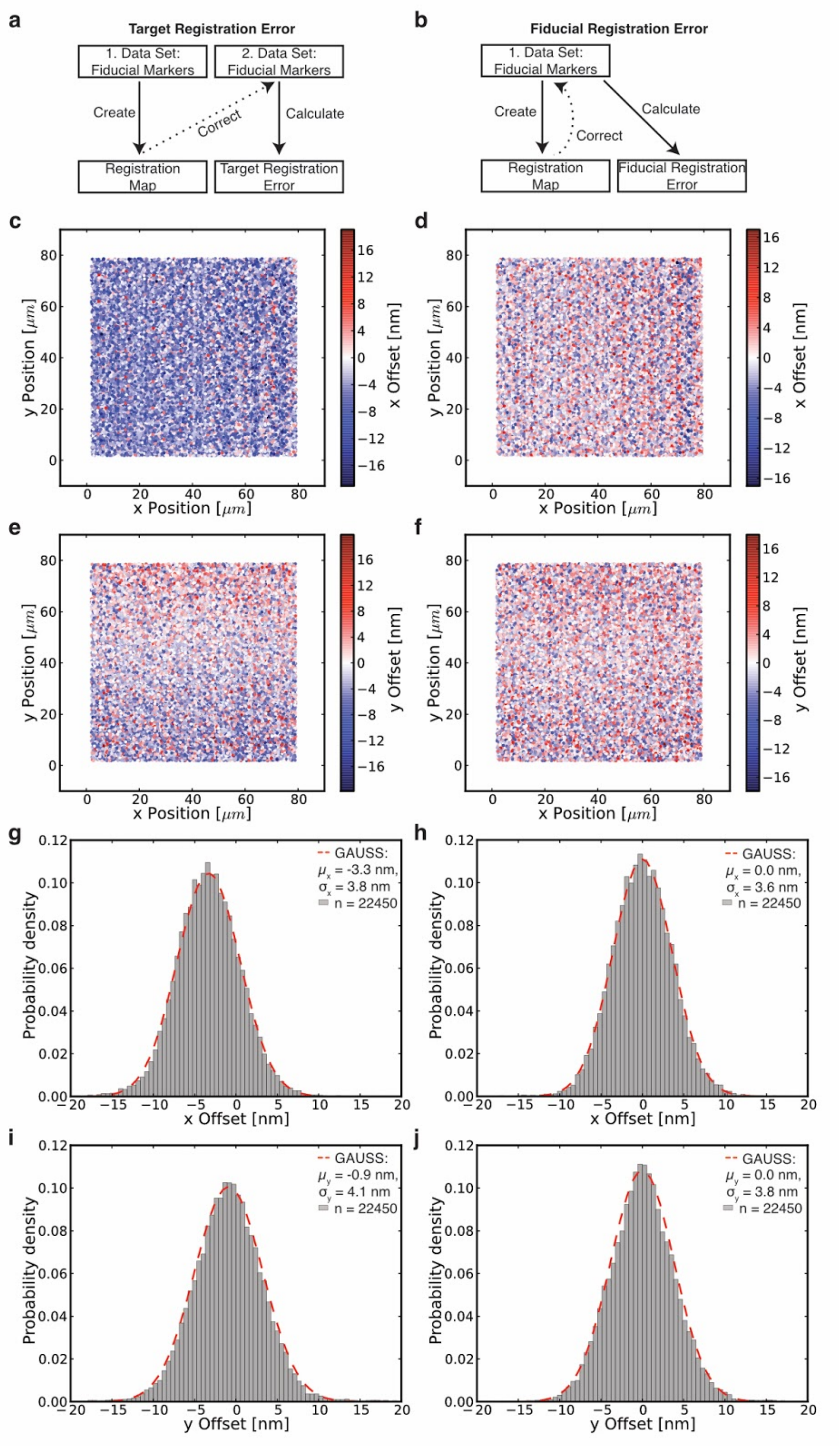

a
Target Registration Error
1. Data Set: Fiducial Markers
2. Data Set: Fiducial Markers
Create
Correct
Calculate
Registration Map
Target Registration Error
b
Fiducial Registration Error
1. Data Set: Fiducial Markers
Create
Correct
Calculate
Registration Map
Fiducial Registration Error
c
d
e
f
y Position [μm]
x Position [μm]
x Offset [nm]
y Offset [nm]
g
h
i
j
Probability density
x Offset [nm]
y Offset [nm]
GAUSS:
μx = -3.3 nm,
σx = 3.8 nm
n = 22450
μx = 0.0 nm,
σx = 3.6 nm
μy = -0.9 nm,
σy = 4.1 nm
μy = 0.0 nm,
σy = 3.8 nm

Fig. S2 | Comparison of target registration error (TRE) and fiducial registration error (FRE) shows that FRE is unreliable and that TRE should always be reported as registration error. The target registration error (TRE) reports the distance (ideally 0) for fiducials other than the points used to create the registration map (2) and is more critical than the fiducial registration error (FRE) which uses the same fiducials to create and test the map. The TRE reports inaccurate image registrations while FRE does not always do so (as shown in this figure). Failure of image registration (as detected by the TRE calculation) is usually caused by a slight change in focus between or during the acquisition of the first and second fiducial marker dataset. Thus, TRE should always be used to evaluate the performance of an image registration process. (a) Workflow for target registration error (TRE) calculation. (b) Workflow for fiducial registration error (FRE) calculation. (c-j) The same registration map is used for both, TRE and FRE, but different datasets are used to test the map. For TRE we use an additional fiducial marker dataset to evaluated the map while for FRE we use the same dataset (fiducial markers) to create and test the registration map. (c) Distance offset along the x-axis for TRE. Each dot shows a single fiducial marker for which the distance offset between the two colors of the same fiducial marker is color-coded. Negative values (blue dots) mean that channel 1 has a smaller number for its position whereas positive values (red dots) represent fiducials where channel 2 has a smaller number for its position. (d) Same as in c but for FRE. (e) Distance offset between the two colors of the same fiducial marker along the y-axis for TRE. (f) Same as in e but for FRE. (g) Histogram of x-axis offsets with Gaussian fit (dashed red line) for TRE. (h) Same as in g but for FRE. (i) Histogram of y-axis offset with Gaussian fit (dashed red line) for TRE. (j) Same as in i but for FRE. One frame per TetraSpeck™ bead was acquired. Details about fitting parameters are in Table S4.

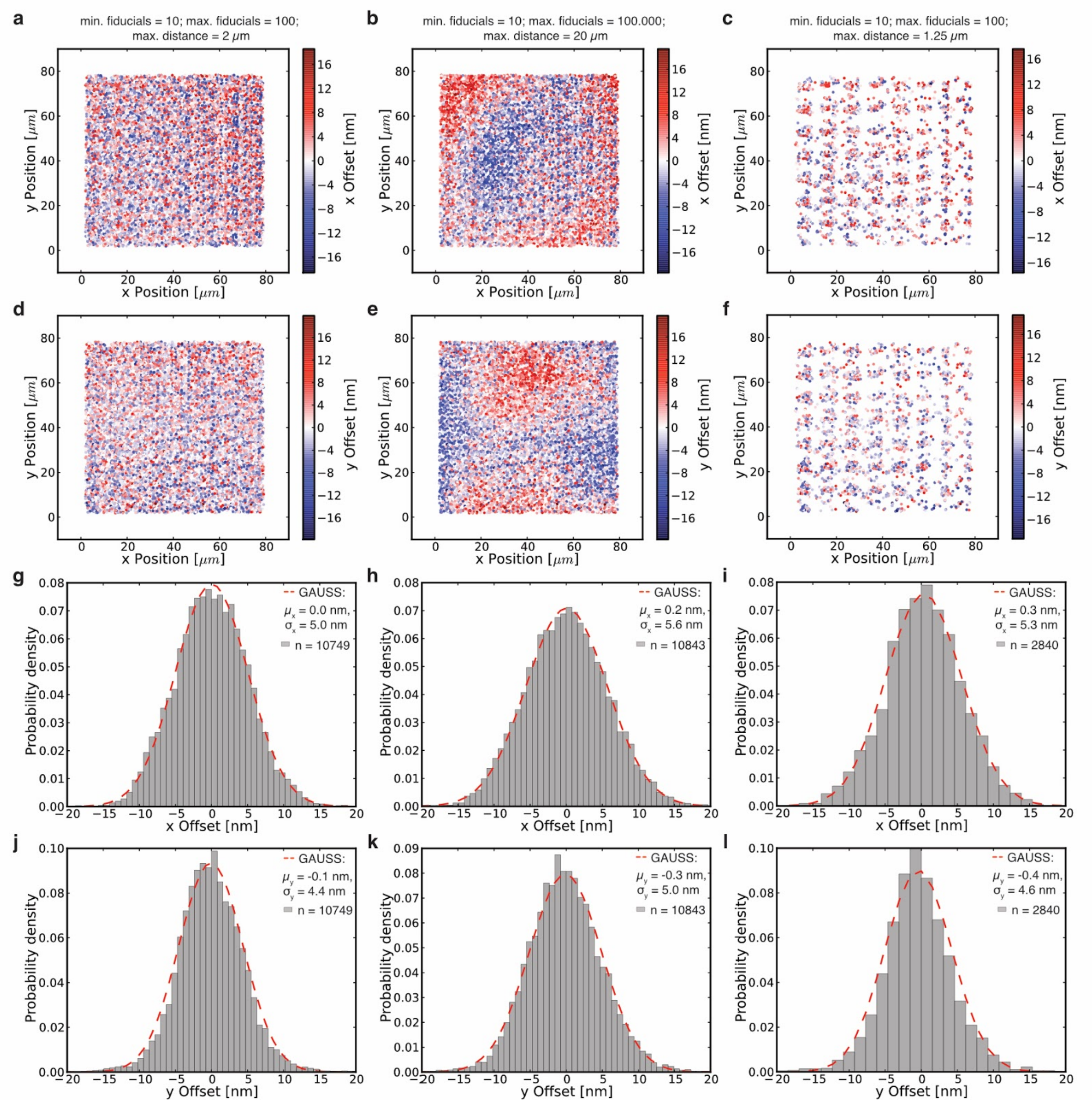

Fig. S3 | Comparison of different parameters for piecewise affine image registration. TetraSpeck™ beads were imaged, localized and registered using a previously determined registration map with different parameter settings for the piecewise affine transformation. Since piecewise affine alignment is based on a nearest neighbor search (3), three parameters can influence registration outcome: minimum and maximum number of fiducial points and the maximum distance to the control point. Higher maximum distance and higher maximum number of points cause distortions indicating that the registration was not executed properly because local effects are not being corrected (as shown in this figure - panels in the middle

column). Thus, setting a maximum distance is important to ensure correction of local distortions. On the other hand, when the maximum distance is too small, an area in the micrograph may not contain the minimum number of fiducials, and thus will not be corrected (as shown in this figure - panels in the right column). This could be overcome by acquiring more fiducials but that is not necessary as we show in this figure and Fig. S4.

The first parameter setting has a minimum of 10, a maximum of 100 fiducial points and a maximum distance of 2 µm. The second parameter setting has a minimum of 10, maximum of 100,000 fiducial points and maximum distance of 20 µm. The third parameter setting uses a minimum of 10, maximum of 100 fiducial points and maximum distance of 1.25 µm. The data presented in this figure clearly shows that there are significant differences between settings for the piecewise affine correction and that a more detailed analysis is needed (see Fig. S4). (a) Distance offset along the x-axis for first setting. Each dot shows a single fiducial marker for which the distance offset between the two colors of the same fiducial marker is color-coded. Negative values (blue dots) mean that channel 1 has a smaller number for its x position whereas positive values (red dots) represent fiducials where channel 2 has a smaller number for its x position. (b) Same as in a but for second setting. (c) Same as in a but for third setting. Registration of many beads fails (hence the sparse number of points) because often less than 10 fiducial points are present within 1.25 µm. (d) Distance offset along the y-axis for first setting. Negative values (blue dots) mean that channel 1 has a smaller number for its y position whereas positive values (red dots) represent fiducials where channel 2 has a smaller number for its y position. (e) Same as in d but for second setting. (f) Same as in d but for third setting. (g) Histogram of x-axis offset with Gaussian fit (dashed red line) of data in a. (h) Same as in g but for second setting and of data in b. (i) Same as in g but for third setting and of data in c. (j) Histogram of y-axis offset with Gaussian fit (dashed red line) of data in d. (k) Same as in j but for second setting and of data in e. (l) Same as in j but for third setting and of data in f. Details about fitting parameters are in Table S4.

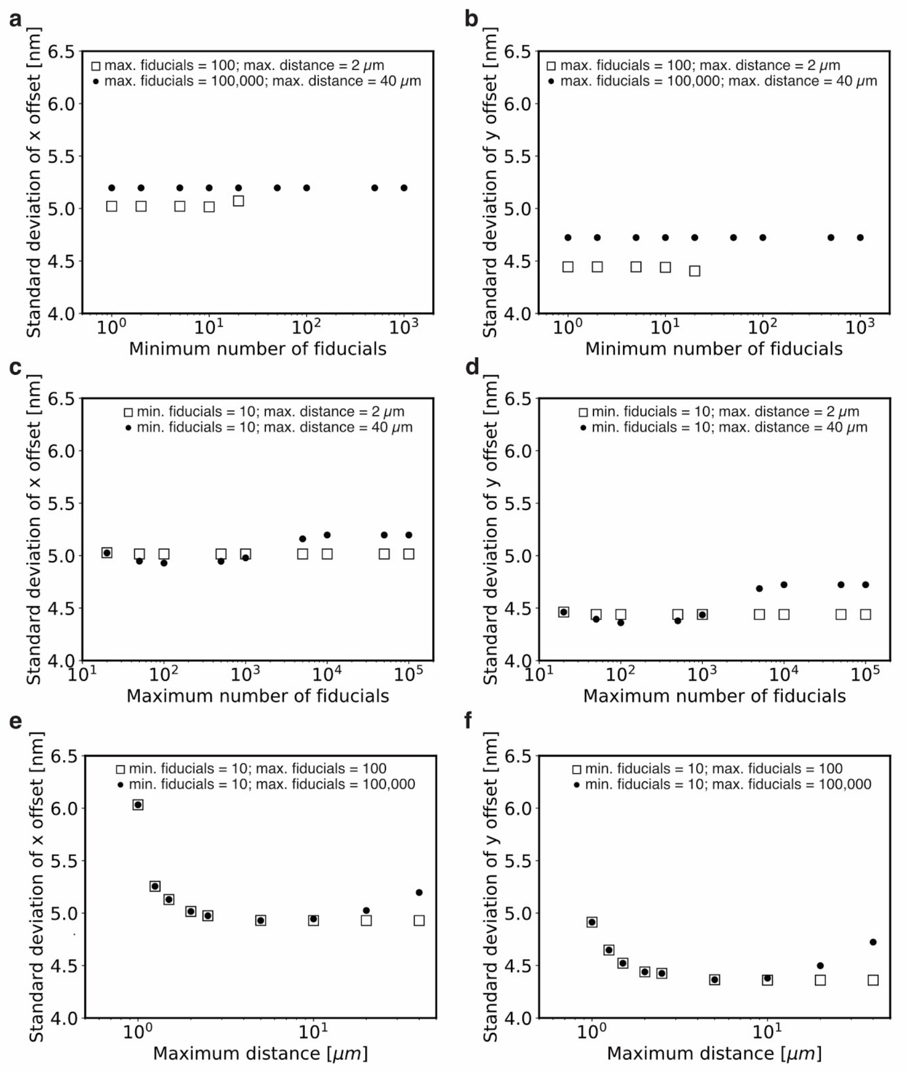

Fig. S4 | Optimization of parameters for piecewise affine image registration. As shown in Fig. S3, different parameter settings for the piecewise affine transformation have a significant effect on the goodness of the registration. Thus, we performed an in-depth analysis of the influence of the following parameter on image registration: minimum and maximum number of fiducial points and maximum distance between points (for a more detailed description of what these parameters mean in the context of image registration see Fig. S3). We are using the standard deviation of registration errors as a measure of goodness of registration over the entire field of view. Hence, the lower the standard deviation of the offset, the better the parameter setting for image registration. (a) Standard deviation of average offset along x-axis as a function of minimum number of fiducials. Results are shown for two different settings of maximum number of fiducials and maximum distance (filled circle and empty square). (b) Same as in a but along y-axis. (c) Standard deviation from average offset along x-axis as a function of maximum

number of fiducials. Results are shown for two different settings of minimum number of fiducials and maximum distance (filled circle and empty square). (d) Same as in c but along y-axis. (e) Standard deviation from average offset along x-axis as a function of maximum distance. Results are shown for two different settings of minimum and maximum number of fiducials (filled circle and empty square). (f) Same as in e but along y-axis. Details about fitting parameters are in Table S4. Overall, the optimal parameter settings for piecewise affine maps are a minimum of 10 and a maximum of 100 fiducial points at a maximum distance of 2 μm.

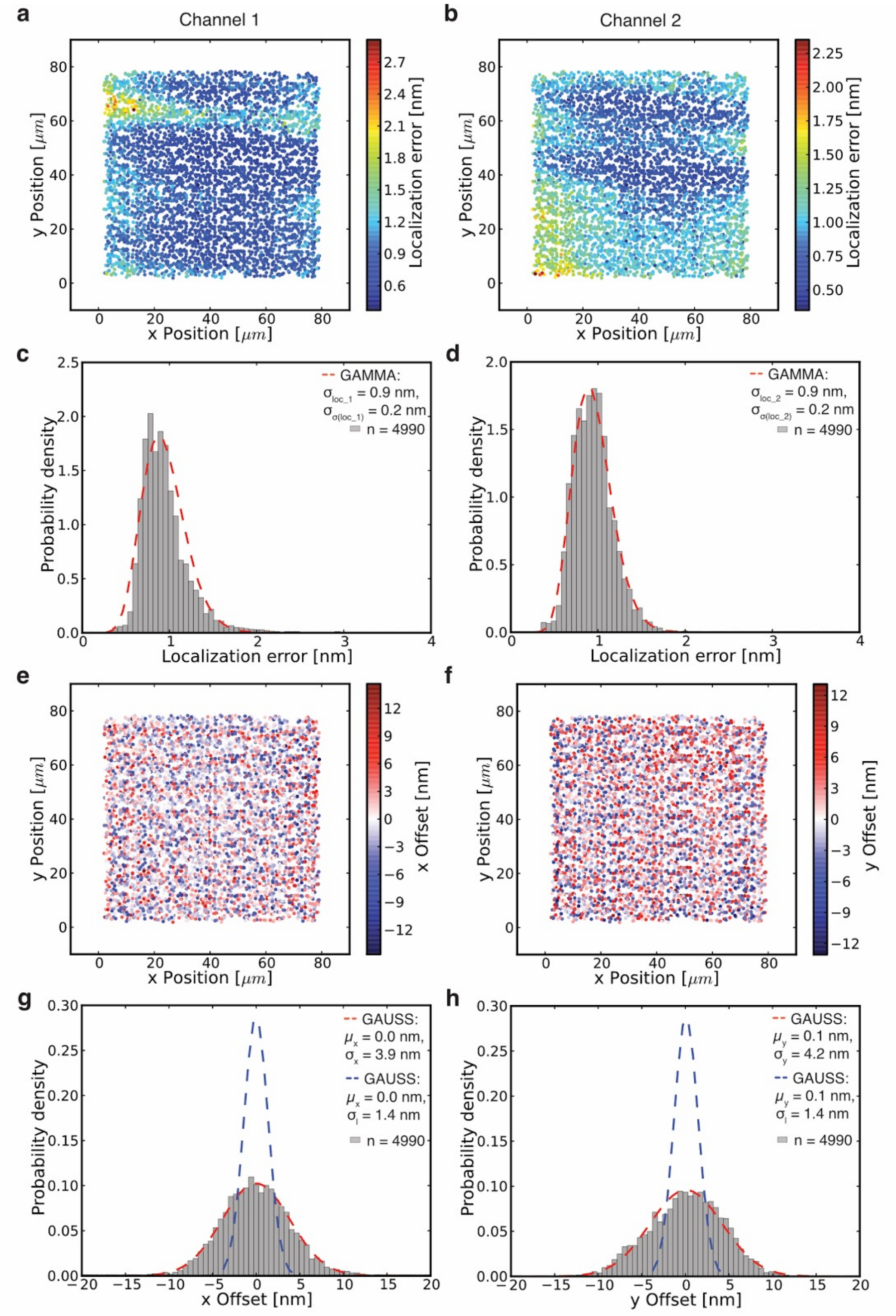

Fig. S5 | Registration precision of TetraSpeck™ beads can not solely be explained by localization errors. If the localization error is the only contributor to the registration imprecision, the uncertainty of localization and registration should be the same. As we see with data, this is not the case for TetraSpeck™ beads because they have non-overlapping color centers (Fig. S6). (a) Localization errors in channel 1 of individual TetraSpeck™ beads (each dot represents a single bead) over the entire field of view calculated with the maximum likelihood with Gaussian (MLEwG) equation from Mortensen et al. (4). Red dots indicate large and blue dots small

localization errors. (b) Same as in a but for channel 2. (c) Histogram of localization errors in channel 1 with fit of gamma distribution (dashed red line) of data shown in a. This clearly shows that the localization errors are different among different beads and that they follow a probability distribution (Fig. S9). (d) Same as in c but for channel 2 and of data in b. (e) Distance offset along the x-axis for same data as shown in a. Negative values (blue dots) mean that channel 1 has a smaller number for its x position whereas positive values (red dots) represent fiducials where channel 2 has a smaller number for its x position. (f) Same as in e but for distance offset along y-axis. (g) Histogram of x-axis offset with Gaussian fit (dashed red line) of data in e. Blue dashed line shows fit if σ_x was only comprised of the localization error σ_l. (h) Same as in g but for offset along y-axis and of data in f. One frame per TetraSpeck™ bead was acquired. Details about fitting parameters are in Table S4.

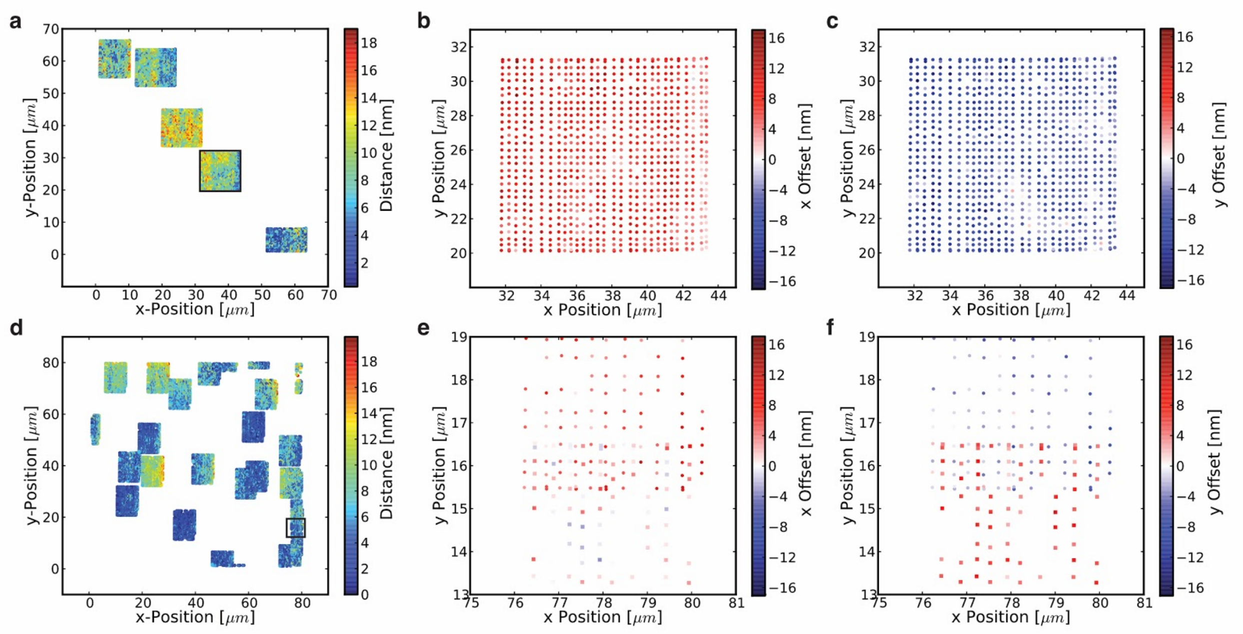

Fig. S6 | Color centers of TetraSpeck™ beads do not overlap perfectly. To investigate whether the distance discrepancies we found with TetraSpeck™ beads (Fig. S5) were due to problems with our registration procedure, or an intrinsic property of the beads, we acquired a registration map, and used it to register many images of the same set of beads, slightly displaced from each other. Two different datasets of fiducial markers (a-c and d-f) show that the distance of individual beads is very stable over time and position (a-c), but that the color centers of these fiducial markers do not overlap (i.e. the distance between the red and far-red channel is not zero nanometer (d-f)). (a) Euclidean distance of image registered, 30x30 grid translated, TetraSpeck™ beads. Each grid shows data for one and the same bead. Distances are color-coded as indicated (blue represents short distances, red large distances). Black box highlights area shown at higher magnification in b and c. (b) Distance offset along the x-axis of magnified part of micrograph in a. (c) Same as in b but distance offset along y-axis. (d) Euclidean distance of image registered, 20x30 grid translated TetraSpeck™ beads. Each grid shows data for one and the same bead. Red dots indicate large and blue dots short distances. Black box highlights area shown at higher magnification in e and f. (e) Distance offset along the x-axis of magnified part of micrograph in d. Here grids of two beads that have overlapping positions are shown where the positions of one bead are shown as circles and the positions of the other bead are shown as squares. (f) Same as in e but distance offset along y-axis. Details about fitting parameters are in Table S4.

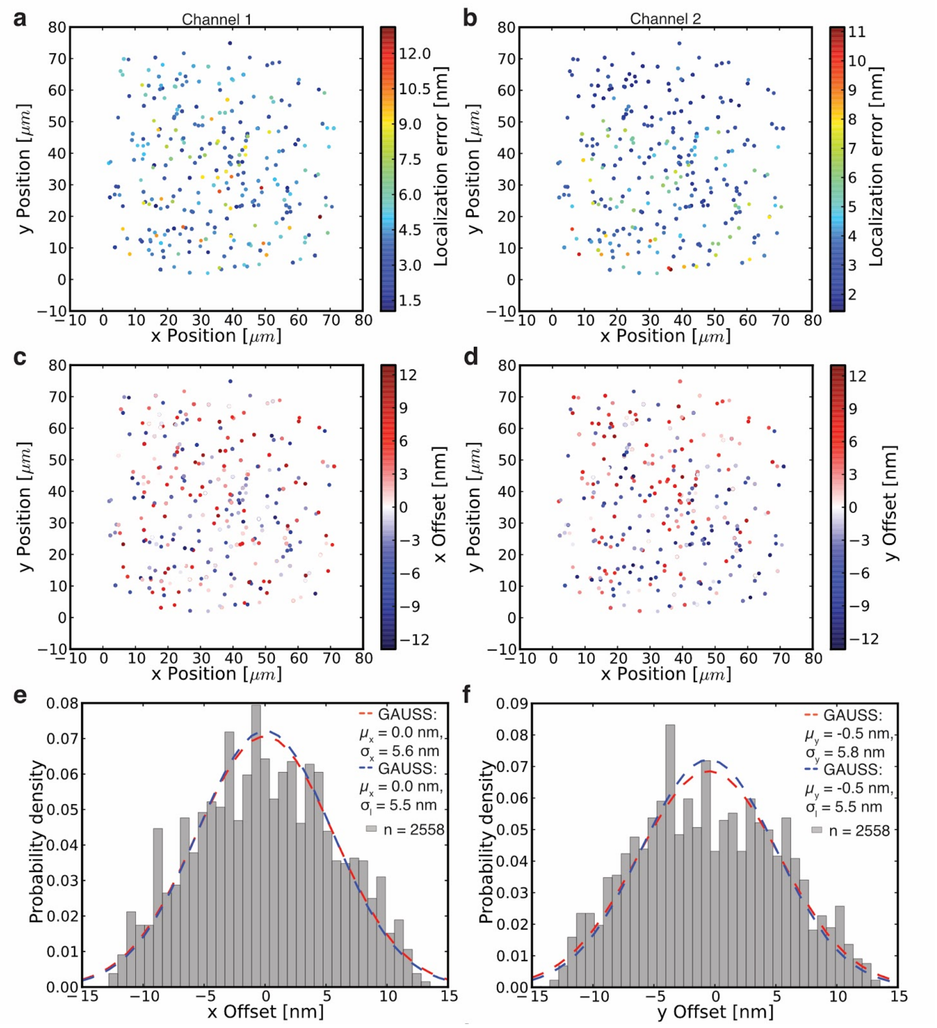

Fig. S7 | Errors in distance measurements are caused by localization errors for a single biotinylated Cy3/Cy5 double stranded DNA (dsDNA) construct. Since the registration imprecision for TetraSpeck™ beads could not fully be explained by localization errors (Fig. S6) but the wider distribution was caused by sample imperfections (non-overlapping color centers), we used a control with perfectly overlapping color centers (5). A 30 bp long dsDNA construct was biotinylated and Cy3 labeled on one end, and Cy5 labeled on the other end (5). While attached to the surface at one end through biotin, the other end is free to rotate. Since the tumbling time is much faster than the acquisition time, we expect an average distance between the color centers of zero nanometers. If the localization error is the only contributor to the registration imprecision, the uncertainty of localization and registration should be the same. As we see in these data, this is indeed the case for this Cy3/Cy5 dsDNA construct. (a) Localization errors in channel 1 of Cy3/Cy5 dsDNA construct over the entire field of view calculated with

MLEwG equation from Mortensen et al. (4). Red dots indicate large and blue dots small localization errors. (b) Same as in a but for channel 2. (c) Distance offset along the x-axis for same data as shown in a. Negative values (blue dots) mean that channel 1 has a smaller number for its x position whereas positive values (red dots) represent fiducials where channel 2 has a smaller number for its x position. (d) Same as in c but for distance offset along y-axis. (e) Histogram of x-axis offset with Gaussian fit (dashed red line) of data in c. Blue dashed line shows fit if σ_x was only comprised of the localization error σ_l. (f) Same as in e but for offset along y-axis and of data in d. 20 frames of each molecule were collected. Single-molecule distances were obtained by selecting time-lapse series of individual molecules (see Table S6). Details about fitting parameters are in Table S4.

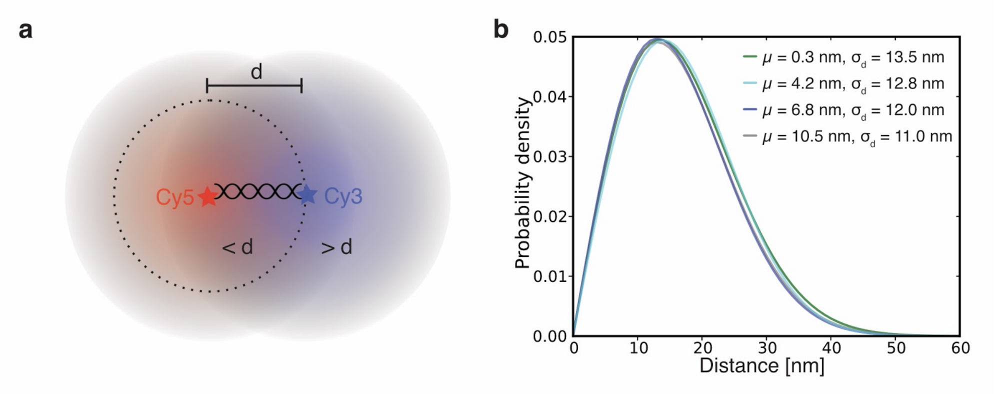

Fig. S8 | Determination of nanometer distances from skewed distributions. (a) This is a graphical explanation of why the distance distribution on the nanometer scale appear non-Gaussian: Top view of two fluorescence intensity distributions that are separated by distance d. Circle with dotted line has a radius d around the position of the Cy5 molecule. Assuming the true position of Cy5 is known, each measurement that finds Cy3 inside the circle will be less than d and measurements finding Cy3 outside the circle are larger than d. Integrating the intensities of the blue molecule inside and outside the circle shows that the total intensity outside the circle is higher than inside. Consequently, the probability for measuring distances larger than d is higher than measuring distances lower than d. Halos represent position / distance uncertainty. (b) Probability distribution (Eq. 2) plotted for various parameter combinations of calculated distance μ and distance uncertainty σ_d shows that small variations in σ_d lead to large changes in estimation of μ.

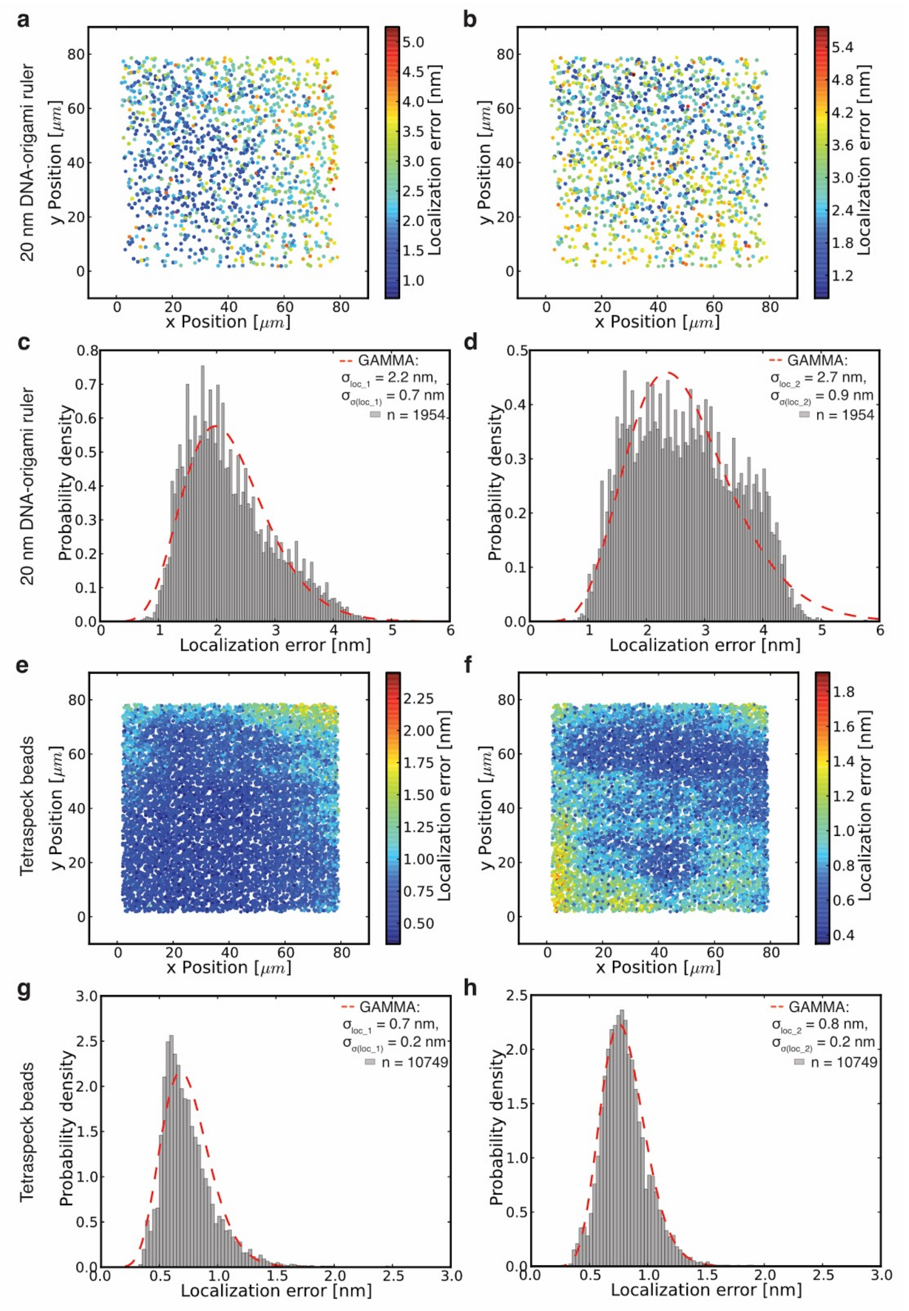

Fig. S9 | Localization errors of many particles have an underlying distribution. Here, we show localization errors over the entire field of view for two different probes in two channels. Quantification of 20 nm DNA-origami nanorulers with 5-10 dyes of Cy3 and 5-10 dyes of Alexa 647 whose center of mass is 20 nm apart (a-d), and of TetraSpeck™ beads (e-h). The variation in localization error among different particles is likely caused by the emission of different number of photons, which itself follows a distribution. (a) Localization errors calculated with MLEwG equation from Mortensen et al. (4) in channel 1 (Cy3 or Cy3 like dye(s)) for 20 nm

DNA-origami nanoruler. Red dots indicate large and blue dots small localization errors. (b) Same as in a but for channel 2 (Cy5 or Cy5 like dye(s)). (c) Histogram of localization error in channel 1 for 20 nm DNA-origami nanoruler with fit of gamma distribution (dashed red line) of data shown in a. (d) Same as in c but for channel 2. (e-h) Same as in a-d but for TetraSpeck™ beads. For 20 nm DNA-origami nanoruler 20 frames per molecule were recorded. For TetraSpeck™ beads one frame per molecule was acquired. Details about fitting parameters are in Table S4.

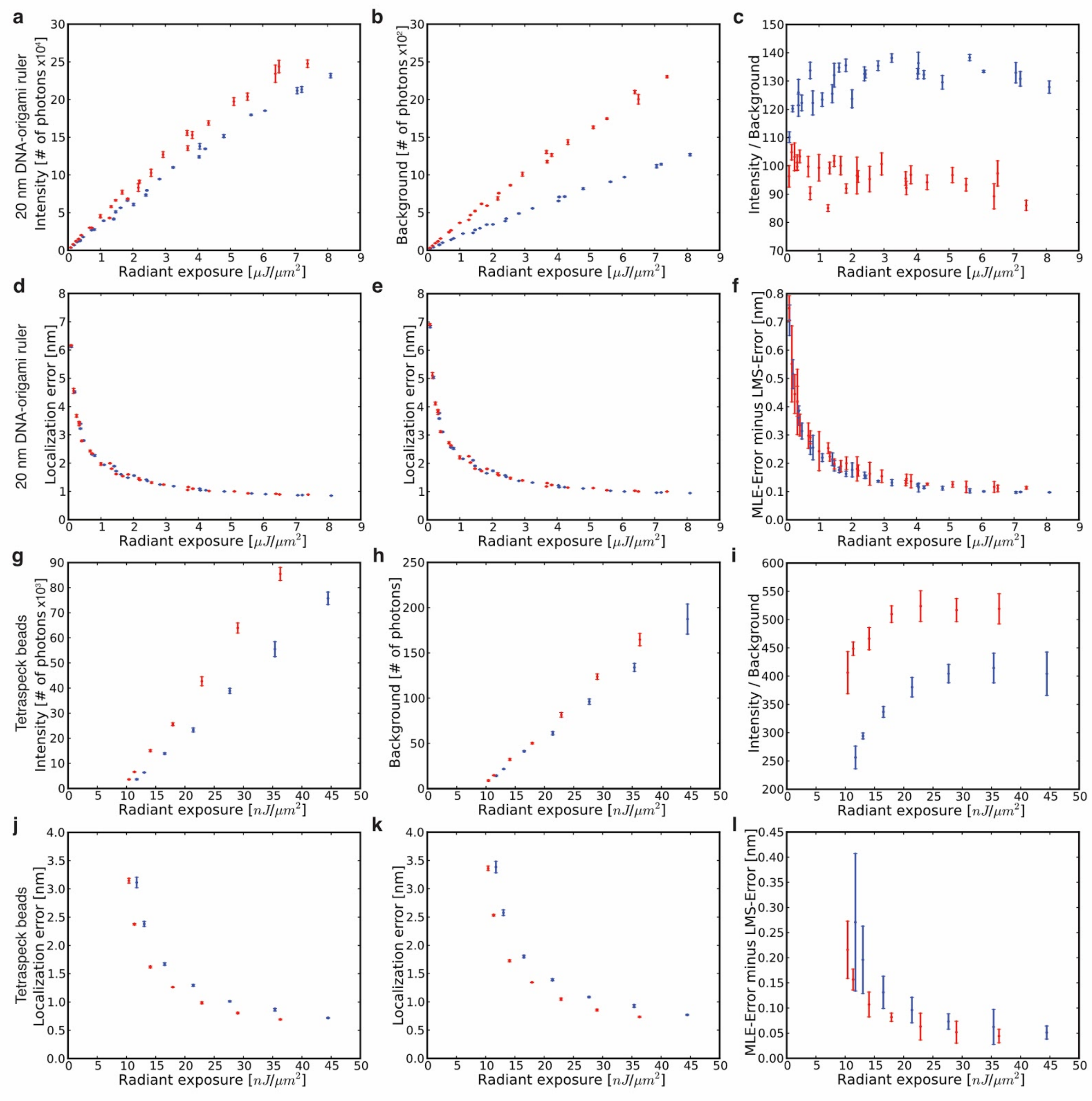

Fig. S10 | Evaluation of photophysical properties of two different probes in two channels. Quantification of 20 nm DNA-origami nanorulers with 5-10 dyes of Cy3 and 5-10 dyes of Alexa 647 whose center of mass is 20 nm apart (a-f), and of TetraSpeck™ beads (g-l). For the intensity as well as the background we expect a linear increase for increasing radiant exposure. Only if for instance photobleaching occurs faster than the acquisition time or if a pixel gets saturated, we expect divergence from the linear behavior. Blue dots show values for channel 1 (Cy3 or Cy3 like dye(s)) and red dots for channel 2 (Cy5 or Cy5 like dye(s)). (a, g) Intensity in number of photons as a function of radiant exposure (Table S2). (b, h) Background in number of

photons as a function of radiant exposure. (c, i) Intensity over background ratio as a function of radiant exposure. (d, j) Localization error calculated with the equation from Thompson et al. (6) (Least Mean Squared (LMS)-Error) as a function of radiant exposure. (e, k) Localization error calculated with the MLEwG equation from Mortensen et al. (4) (MLE-error) as a function of radiant exposure. (f, l) Difference between LMS- and MLE-error as a function of radiant exposure. Error bars in a, b, d, e, g, h, j, and k show standard deviation of five repeats (new microscopy slides with fresh sample). Each repeat consists of at least 100 molecules and one frame was taken per molecule. For c, f, i, and l the error bar is calculated based on error propagation (linear addition). Details about fitting parameters are in Table S4.

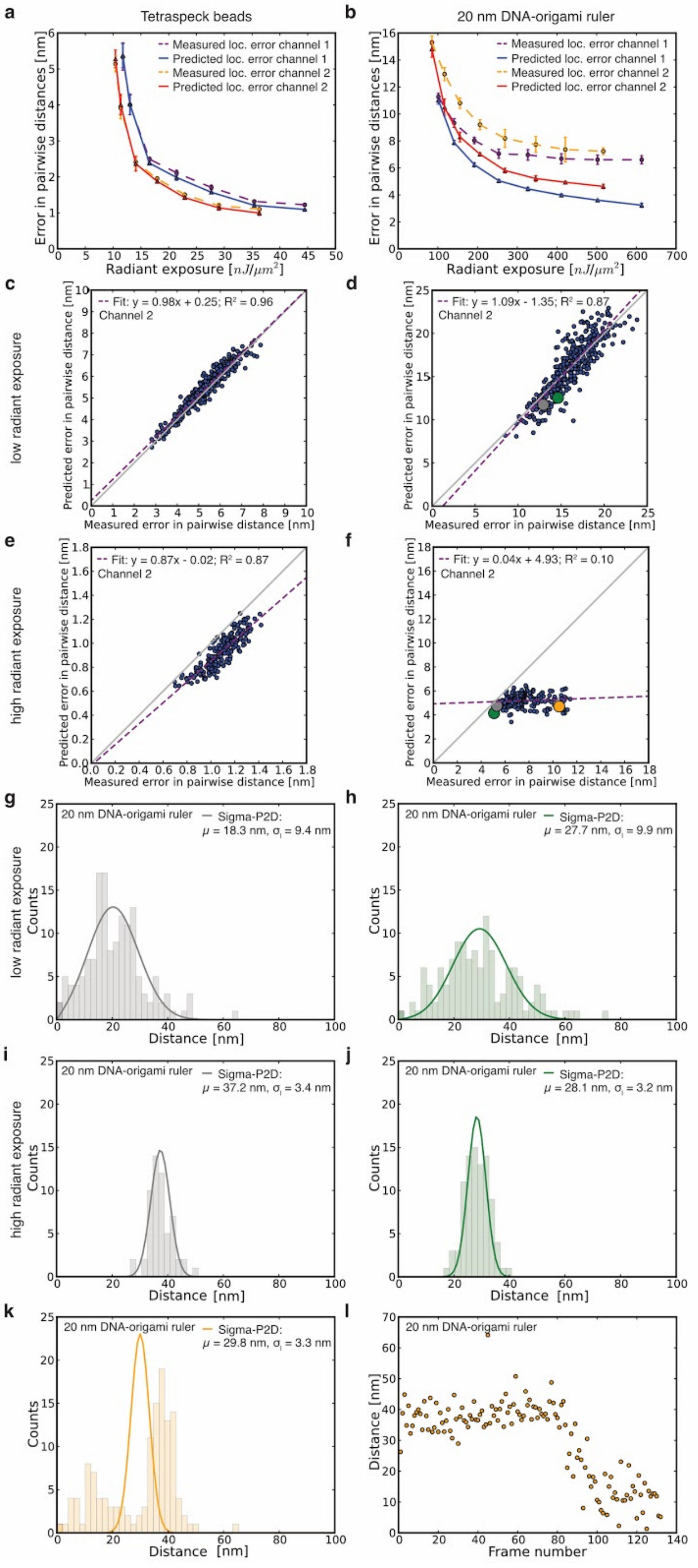

a
Tetraspeck beads
Error in pairwise distances [nm]
Measured loc. error channel 1
Predicted loc. error channel 1
Measured loc. error channel 2
Predicted loc. error channel 2
Radiant exposure [$nJ/\mu m^2$]
b
20 nm DNA-origami ruler
Error in pairwise distances [nm]
Measured loc. error channel 1
Predicted loc. error channel 1
Measured loc. error channel 2
Predicted loc. error channel 2
Radiant exposure [$nJ/\mu m^2$]
c
low radiant exposure
Fit: y = 0.98x + 0.25; R² = 0.96
Channel 2
Predicted error in pairwise distance [nm]
Measured error in pairwise distance [nm]
d
Fit: y = 1.09x - 1.35; R² = 0.87
Channel 2
Predicted error in pairwise distance [nm]
Measured error in pairwise distance [nm]
e
high radiant exposure
Fit: y = 0.87x - 0.02; R² = 0.87
Channel 2
Predicted error in pairwise distance [nm]
Measured error in pairwise distance [nm]
f
Fit: y = 0.04x + 4.93; R² = 0.10
Channel 2
Predicted error in pairwise distance [nm]
Measured error in pairwise distance [nm]
g
low radiant exposure
20 nm DNA-origami ruler
Sigma-P2D:
μ = 18.3 nm, σ = 9.4 nm
Counts
Distance [nm]
h
20 nm DNA-origami ruler
Sigma-P2D:
μ = 27.7 nm, σ = 9.9 nm
Counts
Distance [nm]
i
high radiant exposure
20 nm DNA-origami ruler
Sigma-P2D:
μ = 37.2 nm, σ = 3.4 nm
Counts
Distance [nm]
j
20 nm DNA-origami ruler
Sigma-P2D:
μ = 28.1 nm, σ = 3.2 nm
Counts
Distance [nm]
k
20 nm DNA-origami ruler
Sigma-P2D:
μ = 29.8 nm, σ = 3.3 nm
Counts
Distance [nm]
l
20 nm DNA-origami ruler
Distance [nm]
Frame number

Fig. S11 | Comparison of measured and predicted localization errors for two different probes in two channels. We evaluated how well measured and predicted localization errors correlate. This is important because Sigma-P2D depends on accurate determination of localization errors and small discrepancies will lead to incorrect distance determination. Quantification of TetraSpeck™ beads (a, c, e), and of 20 nm DNA-origami nanorulers with 5-10 dyes of Cy3 and 5-10 dyes of Alexa 647 whose center of mass is 20 nm apart (b, d, f). (a, b) Predicted errors in pairwise distances calculated with MLEwG equation from Mortensen et al. (4) for channel 1 (Cy3 or Cy3 like dye(s)) in blue and channel 2 (Cy5 or Cy5 like dye(s)) in red as function of radiant exposure (Table S3). Measured error (standard deviation) in pairwise distances for channel 1 in purple and channel 2 in orange as function of radiant exposure (for more details see SI Note 3). (c, d) Scatter plot of predicted over measure error in pairwise distances of individual molecules (blue dots) for lowest radiant exposure in channel 2 from data in a, b. Dashed purple line shows fit of linear regression and gray solid line shows theoretical limit (Cramér–Rao lower bound (7)) for localization errors. (e, f) Same as in c and d but for highest radiant exposure setting. For higher radiant exposures, correlations between predicted and measured localization errors were suboptimal. To understand why, we looked at individual molecules for which predicted and measured localization errors correlated poorly, in this case for nanorulers which were imaged at high intensity. For these molecules, we noticed that the distance between colors and therewith either the position of channel 1 or channel 2 changed. Bleaching of individual dyes is likely an important contributor to this position change. The position change leads to an increase in measured localization error since we determined it via pairwise distances (fluctuation in pairwise distance → increase in measured localization error). (d) Larger, colored dots refer to histograms shown in g and h. (f) Larger, colored dots refer to histograms shown in i-k. (g, h) Histogram of distance distribution of a single molecule of a 20 nm DNA-origami nanoruler at low radiant exposure. Solid line is fit with Sigma-P2D. (i, j, k) Same as g and h but at high radiant exposure. (l) Distance as a function of time (frame number) of a single molecule of a 20 nm DNA-origami nanoruler (orange dots). Same data as shown in k. Error bars in a and b show standard deviation of three repeats (new microscopy slides with fresh sample). Each repeat consists of at least 120 pairwise distance measurements, the minimum number of frames per molecule was set to 60 and the maximum number of frames to 200. Single-molecule distances in g-l were obtained by selecting time-lapse series of individual molecules (see Table S6). Details about fitting parameters are in Table S4.

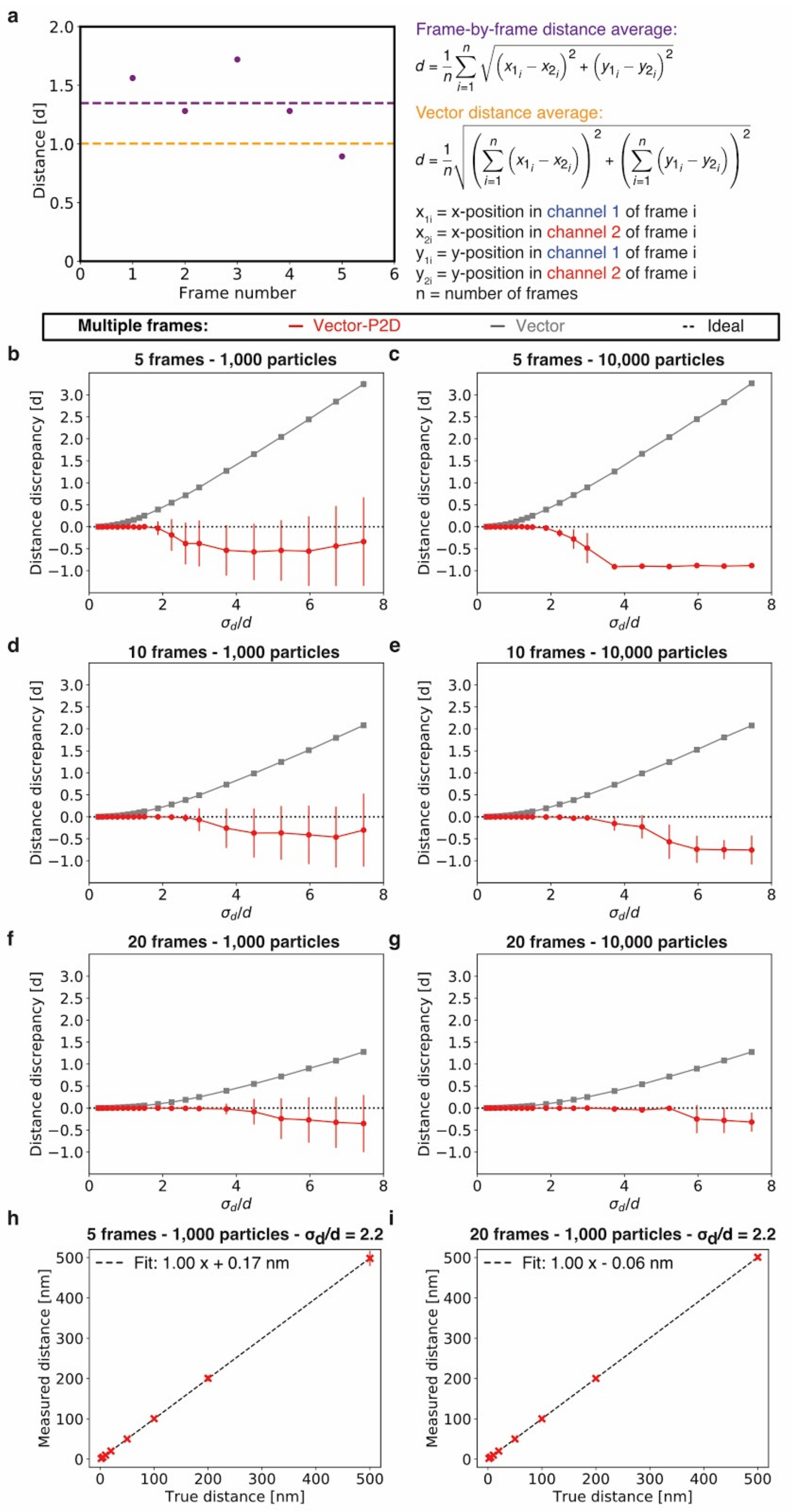
a
Frame-by-frame distance average:
$d = \frac{1}{n}\sum_{i=1}^{n}\sqrt{\left(x_{1_i}-x_{2_i}\right)^2+\left(y_{1_i}-y_{2_i}\right)^2}$
Vector distance average:
$d = \frac{1}{n}\sqrt{\left(\sum_{i=1}^{n}\left(x_{1_i}-x_{2_i}\right)\right)^2+\left(\sum_{i=1}^{n}\left(y_{1_i}-y_{2_i}\right)\right)^2}$
x_{1i} = x-position in channel 1 of frame i
x_{2i} = x-position in channel 2 of frame i
y_{1i} = y-position in channel 1 of frame i
y_{2i} = y-position in channel 2 of frame i
n = number of frames
Distance [d]
Frame number
Multiple frames: Vector-P2D Vector Ideal
b
5 frames - 1,000 particles
c
5 frames - 10,000 particles
d
10 frames - 1,000 particles
e
10 frames - 10,000 particles
f
20 frames - 1,000 particles
g
20 frames - 10,000 particles
Distance discrepancy [d]
σ_d/d
h
5 frames - 1,000 particles - σ_d/d = 2.2
Fit: 1.00 x + 0.17 nm
i
20 frames - 1,000 particles - σ_d/d = 2.2
Fit: 1.00 x - 0.06 nm
Measured distance [nm]
True distance [nm]

Fig. S12 | Performance of distance prediction by Vector-P2D and Vector methods evaluated with Monte Carlo simulated data. (a) Addition to Fig. 4a: Observed distances for each pair (purple dots) and their average (dashed purple line - d = 1.35) as obtained by a frame-by-frame distance average. To calculate the vector average distance (dashed orange line, resulting in d = 1.0), the average distances in x and y are calculated separately first, before combining them in the overall average distance. (b-g) Additional performance results to those shown in Fig. 4c. Here, not 100 particles but 1,000 (b, d, f) and 10,000 (c, e, g) particles were used. We evaluated the performance of Vector-P2D (red) and Vector (grey) by calculating the distance discrepancy (calculated by subtracting the expected distance from the measured distance and normalizing with the expected distance) of Monte Carlo simulated data. Here, the average distance discrepancy for Vector-P2D and Vector from the true distance was calculated using 100 simulations for different ratios of uncertainty σ_d over distance d for 5, 10, and 20 frames. Error bars show standard deviation of 100 independent simulations. The values around -1.0 represent cases for which we measured 0 nm and for which we find very small error bars showing that this is very reproducible. This is an example of a precise yet highly inaccurate measurement. Large error bars typically indicate bimodal cases for which we measured both, distances that are similar to the expected distance and distances that are much smaller than the expected distance. Hence, the increasing size of error bars with increasing σ_d/d ratios shows that the fitting outcome is becoming more and more bimodal until it collapses to one side (measuring distances of around 0 nm). (h, i) Since we always used a true distance of 10 nm in all our simulations, we also tested if Vector-P2D can resolve distances of 2 nm, 10 nm, 20 nm, 50 nm, 100 nm, 200 nm, and 500 nm. We therefore used Monte Carlo simulated data with 1,000 particles, a ratio of distance uncertainty over distance of 2.2, either 5 (h) or 20 frames (i) and the different distances listed above. For each condition we created 100 datasets. We then used Vector-P2D to determine the distance for all datasets and calculated the average distance for each condition. We used this data to calculate the correlation between true and measured distance by determining the slope and found 1.00 for the 5 and 20 frame data which indicates a perfect agreement between true and measured distance. Error bars show the standard deviation.

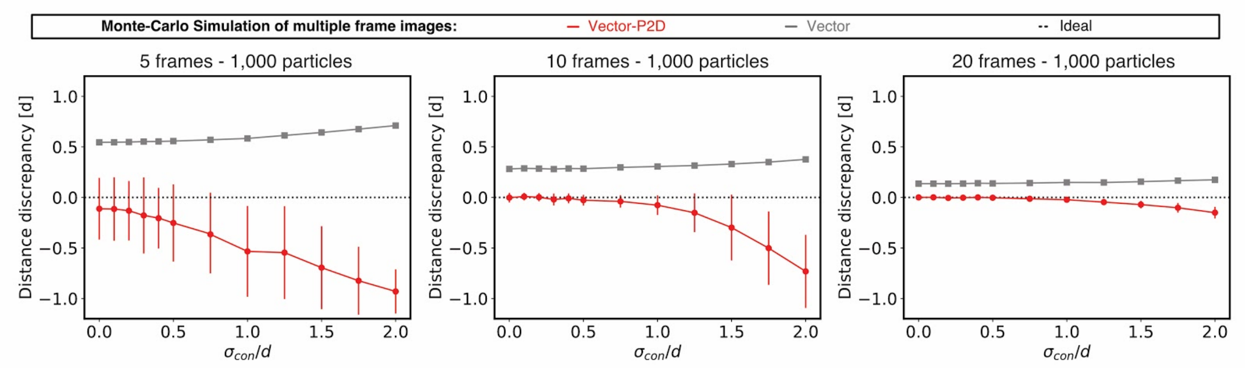

Fig. S13 | Performance of distance prediction as a function of sample heterogeneity of Vector-P2D and Vector using Monte Carlo simulated data by calculating the distance discrepancy (calculated by subtracting the expected distance from the measured distance and normalizing with the expected distance). Here, the average discrepancy from the true distance d for Vector-P2D (red) and Vector (grey) was calculated using 100 simulations for different ratios of sample heterogeneity σ_{con} over distance d for 5, 10, and 20 frames. In all cases we used 1,000 particles and a ratio of distance uncertainty σ_d over distance d of 2.2. Error bars show standard deviation of 100 independent simulations. Large error bars typically indicate bimodal cases for which we measured both, distances that are similar to the expected distance and distances that are much smaller than the expected distance.

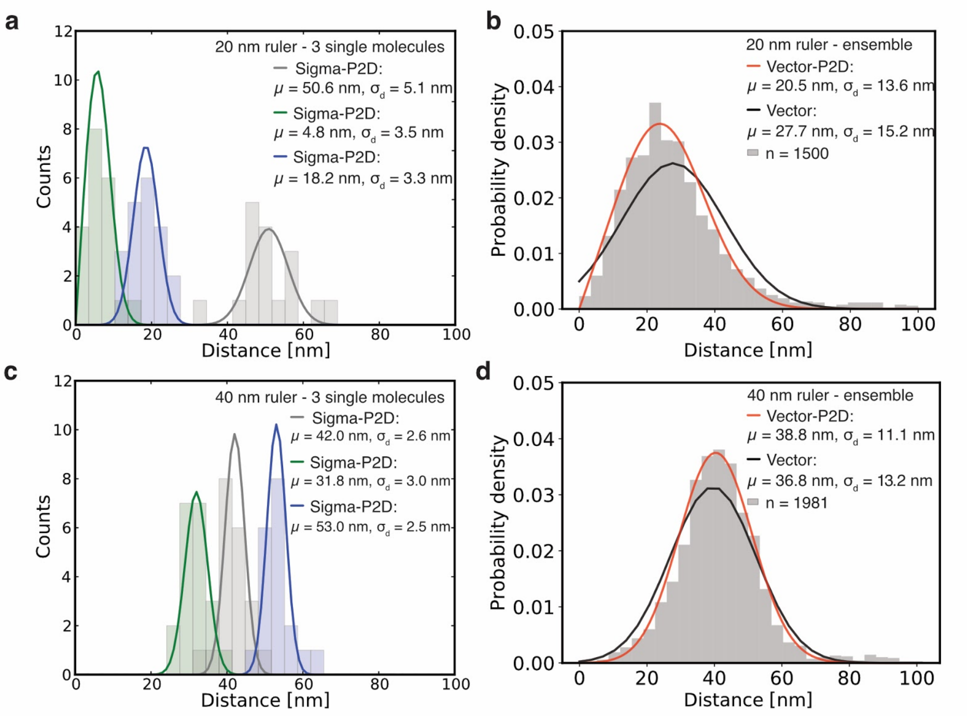

Fig. S14 | Distance measurements for DNA-origami nanorulers. Detailed depiction of results shown in Fig. 4g with distance distributions for single and multiple 20 and 40 nm rulers. (a) Histogram of distance distribution of three different single-molecule 20 nm DNA-origami nanoruler (green, blue, and gray). Solid line is fit with Sigma-P2D. (b) Histogram of vector averaged distance measurements of multiple 20 nm DNA-origami nanorulers analyzed with Vector-P2D (red) and Vector (black). (c) Same as a but for 40 nm DNA-origami nanoruler. (d) Same as b but for 40 nm DNA-origami nanoruler. 20 frames for each molecule in a-d were collected. Details about fitting parameters are in Table S4.

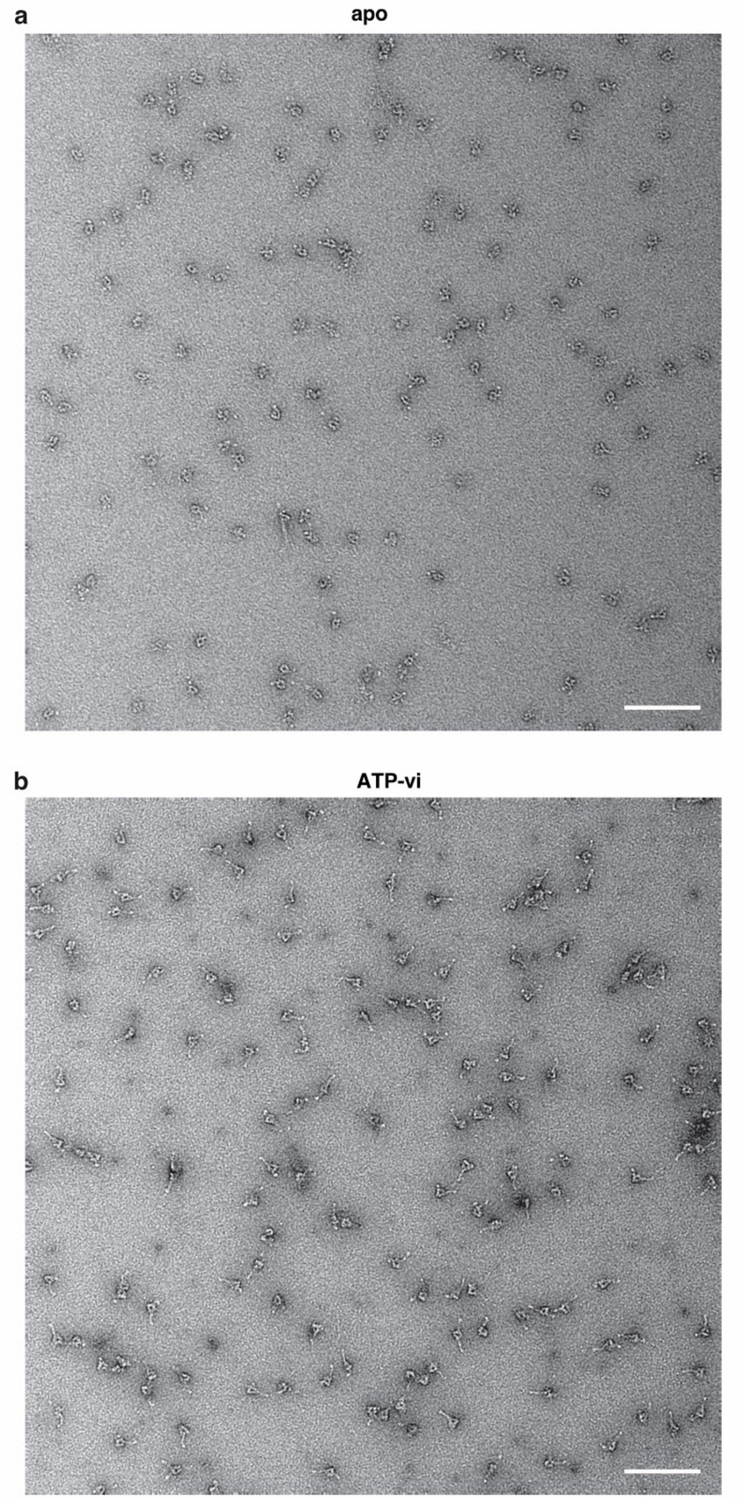

Fig. S15 | Example negative stain electron microscopy micrographs for (a) apo and (b) ATP-vanadate (ATP-vi). For the ATP-vi image, density of the stalk can be seen for many molecules while there is little stalk density for the apo state. Scale bar: 100 nm.

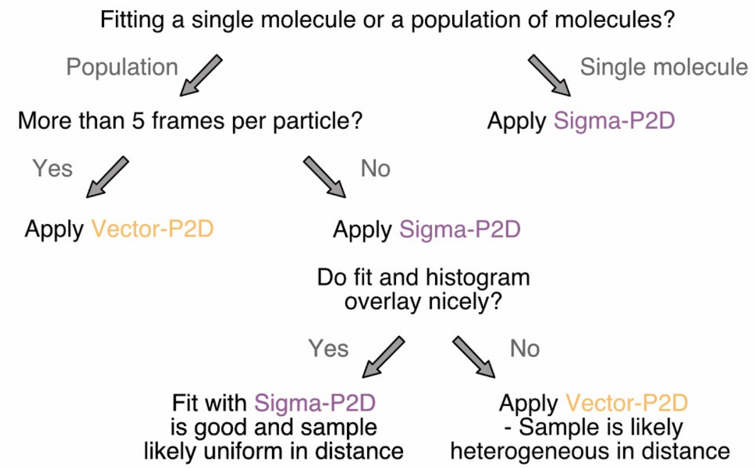

Population or single molecule	Method	Sample uniform or variable in distance	Number of frames	Number of particles	Distance uncertainty over distance cutoff
Population / ensemble	Sigma-P2D	Uniform	1	100	~ 2.0
				1,000	~ 3.5
				10,000	~ 6.0
	Vector-P2D	Uniform	5	100	~ 1.5
				1,000	~ 2.0
				10,000	~ 2.5
	Vector-P2D	Variable	5	100	~ 1.5
				1,000	~ 2.0
				10,000	~ 2.5
			10	100	~ 2.0
				1,000	~ 3.0
				10,000	~ 4.0
			20	100	~ 3.5
				1,000	~ 4.5
				10,000	~ 5.5
Single molecule / particle	Sigma-P2D	-	1	1	~ 0.2
			5		~ 0.5
			10		~ 0.8
			20		~ 1.0

Fig. S16 | Top: Workflow to decide whether to use Sigma-P2D or Vector-P2D. This workflow is based on the strength and limitations of each of the two methods as discussed in the Discussion section of the manuscript. Bottom: Upper bounds for distance uncertainty over distance under given conditions (sample variable or uniform in distance, number of particles, number of frames) until Sigma-P2D or Vector-P2D still deliver reliable results (this is an average distance discrepancy of less than 20% from the true distance with a standard deviation of less than 30% of the true distance).

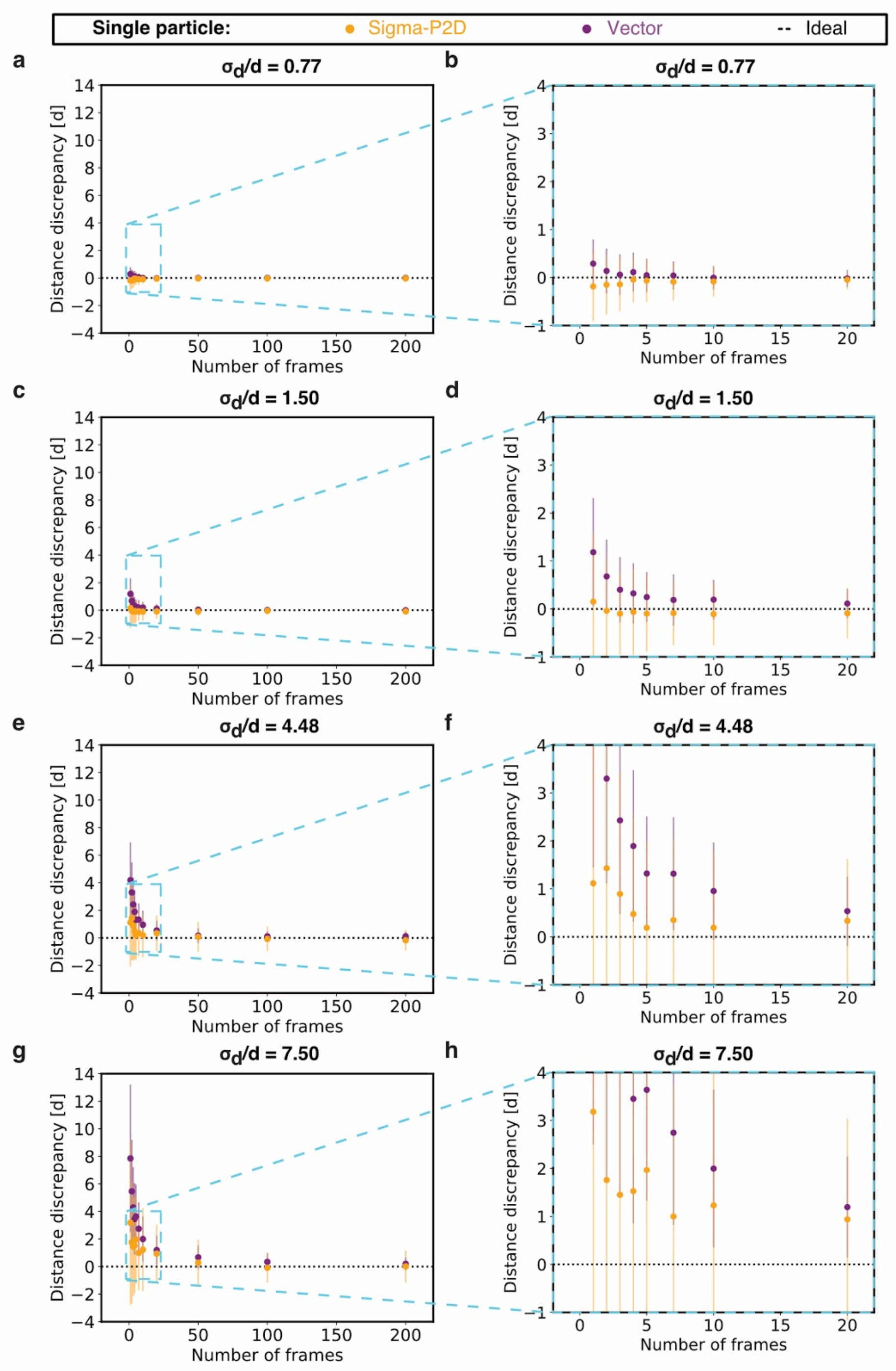

Fig. S17 | Performance of distance prediction as a function of number of frames for Vector and Sigma-P2D on single particles using Monte Carlo simulated data. Since only single particles are analyzed, Vector and Vector-P2D are equivalent because there is only one data point that can be fitted with the P2D function after vector averaging. Thus, we only used the Vector method in this case. We evaluated the performance of Vector and Sigma-P2D by calculating

the distance discrepancy. To do so, we subtracted the expected distance from the measured distance and normalized by the expected distance. Here, the average distance discrepancy for Vector (purple) and Sigma-P2D (orange) was calculated using 100 simulations of single particles for various number of frames (observations). We used different ratios of distance uncertainty σ_d over distance d of 0.77 (a, b), 1.50 (c, d), 4.48 (e, f), and 7.50 (g, h). Blue box in a, c, e, and g is magnified in b, d, f, and h, respectively. Error bars show standard deviation of 100 independent simulations. Large error bars typically indicate bimodal cases for which we measured both, distances that are much larger and distances that are much smaller than the expected distance. As can be seen in this data, Sigma-P2D performs better than or at least equally well as Vector (smaller distance discrepancy) for all conditions when distance distributions of single particles and not ensembles are analyzed.

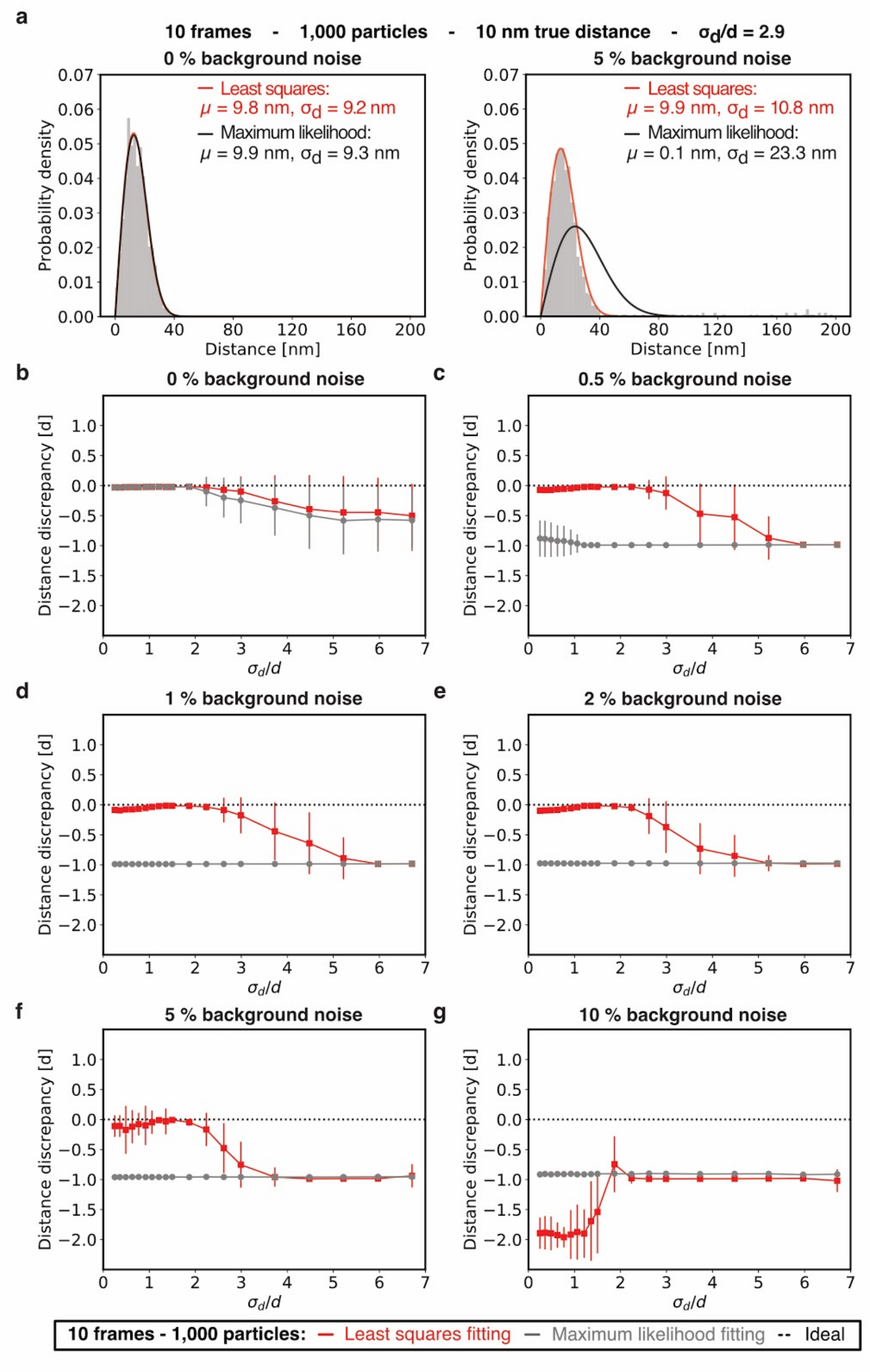

Fig. S18 | Performance of distance prediction by Vector-P2D when fitted by means of maximum likelihood estimation (MLE) or non-linear least squares (NLLSQ) fitting evaluated with Monte Carlo simulated data. (a) Histogram of Monte Carlo simulated data with a true distance d of 10 nm and distance uncertainty σ_d of 29 nm fitted with Vector-P2D by means of MLE (black) and NLLSQ (red). Left: 0% of the data points are background noise. Right: 5 % of all

data points are random background noise over a distance from 0 to 200 nm. (b-g) Average distance discrepancy for Vector-P2D fitted by means of maximum likelihood estimation (MLE) (grey) or non-linear least squares (NLLSQ) (red) based on 100 simulations with different amount of background noise (outlier) and for different ratios of distance uncertainty to distance (σ_d/d). In all cases we used 1,000 particles and 10 frames. We evaluated the performance of MLE and NLLSQ by calculating the distance discrepancy (calculated by subtracting the expected distance from the measured distance and normalizing with the expected distance) of Monte Carlo simulated data. Error bars show standard deviation of 100 independent simulations. The values around -1.0 represent cases for which we measured 0 nm and for which we find very small error bars showing that this is very reproducible. This is an example of a precise yet highly inaccurate measurement. Large error bars typically indicate bimodal cases for which we measured both, distances that are similar to the expected distance and distances that are much smaller than the expected distance. Hence, the increasing size of error bars with increasing σ_d/d ratios shows that the fitting outcome is becoming more and more bimodal until it collapses to one side (measuring distances of around 0 nm). Overall this data shows that NLLSQ fitting is as good as MLE for data lacking background noise and that NLLSQ fitting is as good as or better than MLE fitting in all conditions where a random background noise up to 5% was added (for ratios of distance uncertainty to distance of up to 2). At higher levels of background noise, both methods fail to recover the true distance. Together, we observe that with increasing background noise the NLLSQ fitting becomes more sensitive to higher values of distance uncertainty σ_d.

Supplementary Information Tables

Nucleotide state	full stalk	no stalk	partial stalk	not scored	total
apo	29	1670	157	606	2462
ATP vanadate	2206	104	500	478	3288

Table S1 | Counts of stalk morphology of individual dynein particles in different nucleotide states from negative stain electron microscopy. Number of particles scored as shown in Fig. 5f. Some of the particles could not be assigned to any of the three categories (full stalk, no stalk, partial stalk) because of ambiguity. Thus, these were assigned to a ‘not scored’ category and are not taken into account for calculation of the percentages for Fig. 5f. Procedure of scoring is described in Materials and Methods.

Laser power Ch1 [mW]	Laser power Ch2 [mW]	Exposure time [ms]	Radiant exposure Ch1 [nJ/um^2]	Radiant exposure Ch2 [nJ/um^2]	Noise tolerance - beads	Noise tolerance - 20 nm ruler
0.42	0.38	100	11.75	10.42	100	-
0.47	0.41	100	13.03	11.36	200	-
0.60	0.51	100	16.53	14.08	300	-
0.77	0.65	100	21.42	17.92	400	-
1.00	0.82	100	27.67	22.89	800	-
1.27	1.05	100	35.39	29.03	1,500	-
1.60	1.31	100	44.47	36.31	2,500	-
0.84	0.74	400	93.33	82.22	-	220
0.84	0.74	800	186.67	164.44	-	400
0.84	0.74	1,200	280.00	246.67	-	650
0.84	0.74	1,600	373.33	328.89	-	800
0.84	0.74	2,000	466.67	411.11	-	1,100
3.3	3.0	400	366.7	333.3	-	750
3.3	3.0	800	733.3	666.7	-	1,600
3.3	3.0	1,200	1,100.0	1,000.0	-	2,250
3.3	3.0	1,600	1,466.7	1,333.3	-	2,850
3.3	3.0	2,000	1,833.3	1,666.7	-	3,750
7.3	6.6	400	811.1	733.3	-	1,650
7.3	6.6	800	1,622.2	1,466.7	-	3,300
7.3	6.6	1,200	2,433.3	2,200.0	-	4,500
7.3	6.6	1,600	3,244.4	2,933.3	-	6,000
7.3	6.6	2,000	4,055.6	3,666.7	-	8,000
12.7	11.5	400	1,411.1	1,277.8	-	2,100
12.7	11.5	800	2,822.2	2,555.6	-	5,500

Laser power Ch1 [mW]	Laser power Ch2 [mW]	Exposure time [ms]	Radiant exposure Ch1 [nJ/um^2]	Radiant exposure Ch2 [nJ/um^2]	Noise tolerance - beads	Noise tolerance - 20 nm ruler
12.7	11.5	1,200	4,233.3	3,833.3	-	8,000
12.7	11.5	1,600	5,644.4	5,111.1	-	10,000
12.7	11.5	2,000	7,055.6	6,388.9	-	12,000
18.2	16.6	400	2,022.2	1,844.4	-	3,500
18.2	16.6	800	4,044.4	3,688.9	-	7,000
18.2	16.6	1,200	6,066.7	5,533.3	-	11,000
18.2	16.6	1,600	8,088.9	7,377.8	-	13,000
18.2	16.6	2,000	10,111.1	9,222.2	-	-
21.6	19.5	400	2,400.0	2,166.7	-	4,300
21.6	19.5	800	4,800.0	4,333.3	-	8,800
21.6	19.5	1,200	7,200.0	6,500.0	-	12,500
21.6	19.5	1,600	9,600.0	8,666.7	-	-
21.6	19.5	2,000	12,000.0	10,833.3	-	-

Table S2 | Calculation of radiant exposures as used in Fig. S10. Laser power was measured after the objective. The field of illumination has a 2D Gaussian shape (reflecting the Gaussian shape of the laser beam), causing the radiant exposure to vary over the field of view. Here, we calculated an average radiant exposure by assuming a field of illumination of 60 µm by 60 µm. Noise tolerance refers to settings in µManager's (1) 'Localization Microscopy' plug-in. It was chosen so that approximately the same number of molecules were fitted per micrograph and to avoid fitting background as true particles (especially for higher radiant exposures). More details about fitting parameters in Table S4.

Laser power Ch1 [mW]	Laser power Ch2 [mW]	Exposure time [ms]	Radiant exposure Ch1 [nJ/um^2]	Radiant exposure Ch2 [nJ/um^2]	Noise tolerance - beads	Noise tolerance - 20 nm ruler
0.42	0.38	100	11.75	10.42	100	-
0.47	0.41	100	13.03	11.36	200	-
0.60	0.51	100	16.53	14.08	300	-
0.77	0.65	100	21.42	17.92	400	-
1.00	0.82	100	27.67	22.89	800	-
1.27	1.05	100	35.39	29.03	1,500	-
1.60	1.31	100	44.47	36.31	2,500	-
0.91	0.77	400	101.33	85.11	-	50
1.27	1.05	400	141.00	116.78	-	80
1.73	1.40	400	192.00	155.78	-	95
2.28	1.86	400	253.33	206.11	-	135
2.94	2.42	400	326.78	269.11	-	175
3.7	3.1	400	411.1	346.7	-	230
4.5	3.8	400	502.2	421.1	-	270
5.5	4.7	400	613.3	516.7	-	350

Table S3 | Calculation of radiant exposures as used in Fig. S11. Laser power was measured at objective. The field of illumination has a 2D Gaussian shape (reflecting the Gaussian shape of the laser beam), causing the radiant exposure to vary over the field of view. Here, we calculated an average radiant exposure by assuming a field of illumination of 60 µm by 60 µm. Noise tolerance refers to settings in µManager's (1) 'Localization Microscopy' plug-in. It was chosen so that approximately the same number of molecules were fitted per micrograph and to avoid fitting background as true particles (especially for higher radiant exposures). More details about fitting parameters in Table S4.

**Imaging Parameters**	
Photon conversion factor	1.84
Linear (EM) gain	1.0
Pixel size [nm]	159.0
Time interval [ms]	0.0
Z-step [nm]	50.0
Camera offset [electron counts]	91.0
Read noise [electron counts]	9.84
**Find Maxima**	
Pre-Filter	None
Noise tolerance	See **Table S5**
**Fit Parameters**	
Dimensions	1
Filter	Simplex-MLE
Max Iterations	500
Box size [pixel]	12.0
Fix width	Not selected
**Filter Data**	Nothing selected
**Positions**	Always all, except for registration maps where data is split to create affine and piecewise affine maps so that ~1000 beads can be used for affine map. Remainder is used for piecewise affine map.
**Skip Channels**	Not selected

Table S4 | Fitting parameters used in µManager's (1) 'Localization Microscopy' plug-in.

**Dataset in Figure**	**Radiant exposure used for sample of interest** [nJ/um^2]		**Noise tolerance used for sample of interest**
	**Channel 1**	**Channel 2**	
Fiducials to create *all maps*	35.39	29.03	300
Figure 2	35.39	29.03	300
Figure 3	613.3	516.7	100
Figure 4	613.3	516.7	100
Figure 5	1,982.0	1,853.3	100
Supplementary Figure 2	35.39	29.03	300
Supplementary Figure 3	35.39	29.03	300
Supplementary Figure 4	35.39	29.03	300
Supplementary Figure 5	35.39	29.03	300
Supplementary Figure 6	35.39	29.03	300
Supplementary Figure 7	2,400.0	2,166.7	300
Supplementary Figure 9	20 nm ruler:<br>613.3<br>Beads:<br>35.39	20 nm ruler:<br>516.7<br>Beads:<br>29.03	All datasets:<br>300
Supplementary Figure 10	See **Table S2**		See **Table S2**
Supplementary Figure 11	See **Table S3**		See **Table S3**
Supplementary Figure 14	613.3	516.7	100

Table S5 | Radiant exposures used for the acquisition of different datasets if not specified elsewhere. Noise tolerance as used in the µManager's (1) 'Localization Microscopy' plug-in for different datasets if not specified elsewhere.

**Dataset in Figure**	**Minimum # of Frames**	**Maximum # of missing Frames**	**Maximum Distance [nm]**	**Minimum total distance [nm]**	**Combine tracks from all channels**	**Maximum pair distance [nm]**
Figure 3	e, f: 5	e, f: 15	e, f: 15	e, f: 0	e, f: Checked	e, f: 30
Figure 5	c, d: 15	c, d: 5	c, d: 25	c, d: 0	c, d: Checked	c, d: 100
Supplementary Figure 7	3	17	20	0	Checked	20
Supplementary Figure 11	g-l: 60	g-l: 10	g-l: 90	g-l: 0	g-l: Unchecked	g-l: -

Table S6 | Settings for the ‘Extract Tracks’ function in the µManager’s (1) ‘Localization Microscopy’ plug-in used to extract tracks of single molecules that were imaged for F number of frames. Here the maximum distance refers to the distance between the position of one molecule from channel n-1 to channel n. The minimum total distance indicates the distance of one molecules’ position in frame 1 to its position in frame F. Maximum pair distance selects molecules in which the distance between channel 1 and channel 2 position is below a defined threshold.

Supplementary Information Note 1

Color centers of TetraSpeck™ beads do not overlap

For TetraSpeck™ beads the registration precision (σ_x, σ_y) is worse than one would expect based on their localization errors σ_l (σ_{loc_1}, σ_{loc_2} and their variances $\sigma_{\sigma(loc\_1)}$, $\sigma_{\sigma(loc\_2)}$) (Fig. S5). To assess if this discrepancy is due to our image registration procedure or an intrinsic parameter of the beads, we imaged individual beads translated in a 30x30 grid pattern and noticed that most beads have a consistent x-y-distance offset, independent of the position in the image (Fig. S6a-c). In an area where grids of two different beads (the positions of one bead are shown as circles and the positions of the other bead are shown as squares) overlap we observed that the offset in x and y was a function of the individual bead, not the position in the image (Fig. S6d-f). As reported by others (5, 8), this strongly suggests that the offset is caused by beads for which the color centers of the two dyes do not exactly overlap but differ by a few nanometers. To test if the registration precision can solely be explained by localization errors σ_l of the test sample we turned to a single biotinylated Cy3/Cy5 dsDNA construct, which has been shown to have no distance variation across molecules and to be of zero distance (5). For this sample we found very good agreement between μ_x and σ_l as well as μ_y and σ_l (Fig. S7). Thus, these experiments suggest that the sample's localization error σ_l can almost solely account for the registration precision σ_{reg} as long as the sample is uniform in distance and the registration accuracy is high (< 1 nm).

Supplementary Information Note 2

The two-dimensional probability distribution (9) is given by

$$p_{2D}(r) = \left(\frac{r}{\sigma_d^2}\right) exp\left(-\frac{\mu^2+r^2}{2\sigma_d^2}\right) I_0\left(\frac{r\mu}{\sigma_d^2}\right) \quad (5)$$

in which r is the measured Euclidean distance, μ the calculated distance, σ_d the distance uncertainty, and I_0 the modified Bessel function of integer order zero. For $\sigma_d \gg \mu$, r similar order or less than σ_d, and with

$$I_0(z) = \sum_{k=0}^{\infty} \frac{\left(\frac{1}{4}z^2\right)^k}{(k!)^2} \quad (6)$$

we can find the following approximation:

$$p_{2D}(r) \approx \left(\frac{r}{\sigma_d^2}\right) exp\left(-\frac{\mu^2+r^2}{2\sigma_d^2}\right)\left(1+\frac{r^2\mu^2}{4\sigma_d^4}+...\right) \quad (7)$$

$$\approx \left(\frac{r}{\sigma_d^2}\right) exp\left(-\frac{r^2}{2\sigma_d^2}\right)\left(1-\frac{\mu^2}{2\sigma_d^2}+...\right)\left(1+\frac{r^2\mu^2}{4\sigma_d^4}\right) \quad (8)$$

$$\approx \left(\frac{r}{\sigma_d^2}\right) exp\left(-\frac{r^2}{2\sigma_d^2}\right)\left(1+\frac{\mu^2}{2\sigma_d^2}\left(\frac{r^2}{2\sigma_d^2}-1\right)\right) \quad (9)$$

$$\approx \left(\frac{r}{\sigma_d^2}\right) exp\left(-\frac{r^2}{2\sigma_d^2}\right) \quad (10)$$

Thus, the approximation for $\sigma_d \gg \mu$ of the probability distribution (Eq. 2 / 4) is independent of μ.

Supplementary Information Note 3

Predicted and measured localization errors correlate well

Sigma-P2D can only be used with experimental data if the fluorophores' localization errors can be determined with high accuracy, because imprecise predictions lead to incorrect distance estimates (Fig. S8b). To test the available theoretical predictions of localization errors, we first imaged fluorescent probes that varied in number of fluorophores and determined intensities, backgrounds and widths using the MLE Gaussian fit (4). Based on these measurements, we compared the localization errors given by the equations of Thompson et al. (6) and the MLEwG method described by Mortensen et al. (4) (Fig. S10). Both equations yielded fairly similar values, with the MLEwG resulting in slightly higher localization errors, as reported previously (4, 10).

We then investigated the standard deviation of localization errors ($\sigma_{\sigma(loc\_1)}$, $\sigma_{\sigma(loc\_2)}$) for fluorescent probes with a cluster of up to ten Cy3 and Alexa 647 dyes (20 nm nanorulers), and of TetraSpeck™ beads. We found that localization errors follow a probability distribution (Fig. S9) that depends amongst other things on the illumination pattern and the number of fluorophores per particle. Thus, in order to plot a fit for Sigma-P2D of many molecules the distance uncertainty $\sigma_{d\text{-}adj}$ has to be adjusted to

$$\sigma_{d-adj} = \sqrt{\sigma_{reg}{}^2 + \sigma_{loc_1}{}^2 + \sigma_{loc_2}{}^2 + \sigma_{\sigma_{loc_1}}{}^2 + \sigma_{\sigma_{loc_2}}{}^2}\ . \qquad (11)$$

Note, that the distance μ is still determined as described above by performing MLE for individual (single) particles using equations 2 and 3 and we only need this adjusted distance uncertainty $\sigma_{d\text{-}adj}$ if we plot a fit for a histogram with distances of many particles.

Next, we measured localization errors at various radiant exposures and compared these to those predicted by equations. We measured the variations in probe position in a time-lapse sequence to determine the experimental localization error. We determined the localization error of each probe by averaging the variance of its pairwise distance with each of the other probes in the image over more than 120 frames (see Materials and Methods). The error predicted by the MLEwG fit for a single probe was calculated from the average pairwise localization error of that probe with all other probes from the same dataset. Interestingly, the measured and

predicted localization errors correlate well ($R^2 \geq 0.8$) at low radiant exposures (Fig. S11) but poorly for one of the two samples at high radiant exposures (Fig. S11f). We discovered that the poor correlation was caused by intrasample distance changes during the measurements likely caused by photobleaching (Fig. S11). To test this further we performed Sigma-P2D on single molecules from the same dataset (Fig. S11g-l) and noticed that the discrepancy between measured and predicted localization errors for samples with few fluorophores at high radiant exposures is mainly due to intrasample distance changes (bleaching of dyes and change in 'center-of-mass' - distance between the two colors) during measurements (Fig. S11k, l). Hence, outliers for which the predicted and measured localization errors do not match are likely caused by sample imperfections. Taken together, the localization errors predicted by the MLEwG method described by Mortensen et al. (4) match the experimentally measured values well.

Supplementary Information Protocol

In the following sections we provide a detailed protocol for data acquisition, fitting of emitters, image registration, and data analysis (distance determination) in μManager (1) 2.0. In addition, we provide screenshots of the relevant plugins.

a) Data acquisition

1. Data is acquired in the order depicted in Fig. 2a and as described in the Materials and Methods. For all data collections we used the 'Multi-Dimensional Acquisition' plugin. A detailed manual for data acquisition can be found [here](). Here we only describe the grid acquisition used for fiducial markers.
2. For the fiducial markers to be used for the affine and piecewise affine maps as well as the fiducials used to test the map acquired after the sample of interest, select 'Multiple Positions', 'Channels' and 'Save Data' in the 'Multi-Dimensional Acquisition' plugin. Then, click on 'Edit Position List'. A new window 'Stage Position List' will appear.

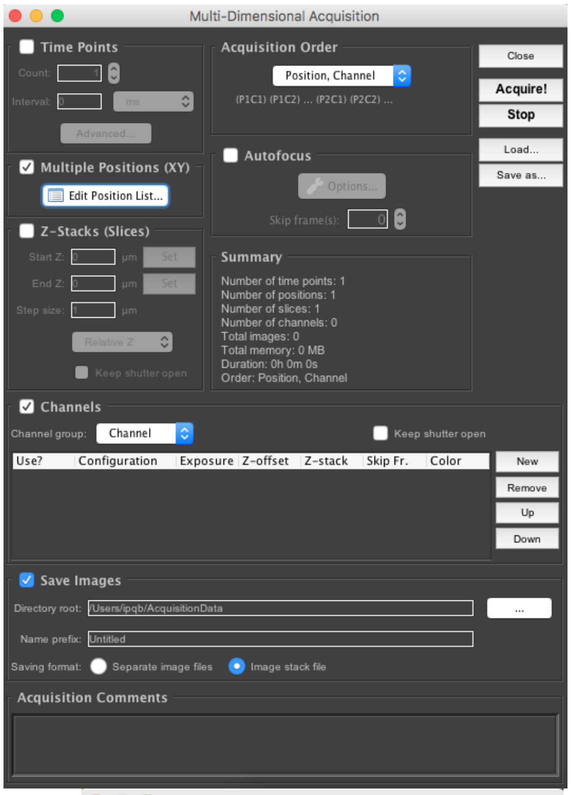

3. If there are entries in the table, click 'Clear All' first. Then click 'Create Grid'. An additional window 'Tile Creator' will open. By clicking '+' and '-' you can increase or decrease the grid size, respectively. Typically, you want to create a 20x20 grid. Afterwards type the overlap size and provide the pixel size. We used an overlap of 71 μm for a micrograph of 81x81 μm. Next, click 'Center Here' and then 'OK'.

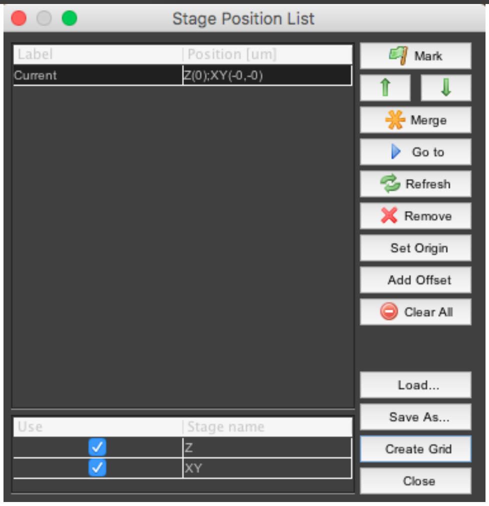

4. In the ‘Stage Position List’ window all generated positions for your grid will be listed in the table. If everything looks good, click ‘Close’.
5. For how to change ‘Channel’ and ‘Save Data’ settings see here. Once everything is set, click ‘Acquire!’.

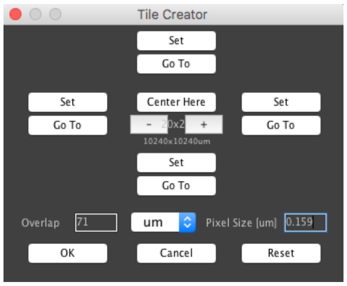

b) Fitting emitters

6. Open the data with the fiducial markers that were acquired **prior** to the sample of interest by either dragging and dropping the file into μManager or by ‘File’ → ‘Open (RAM)’.
7. Open the ‘Localization Microscopy’ plugin: “Plugins’ → ‘Acquisition Tools’ → ‘Localization Microscopy’ that looks like the one on the bottom right of this page.
8. Provide the ‘Imaging parameters’ that match your settings. For the ‘Find Maxima’ you can use the ‘show’ button to see which particles will get selected with the corresponding ‘Noise tolerance’. For the ‘Fit Parameters’ you want ‘Dimensions’ to be ‘1’, and use the ‘Simplex MLE’ ‘Fitter’. We always used ‘500’ as ‘Max Iterations’. The ‘Box Size’ should not be too small because this will overestimate the background. Typically, the box size is right when the localization error no longer changes with increasing box size. You can ‘Filter Data’ to remove outliers (optional). If you run any of the example datasets that we provide, use the values listed in Table S4.
9. In ‘Positions’ (Positions of your grid (e.g. you may have acquired a 20x20 grid and thus 400 images total)) start with ‘1-20’ and adjust so that you get approximately 1,000 spots to create your affine map. Then click ‘Fit’. A new window ‘Gaussian tracking data’ will open (see below). The number of spots will be listed in the third column.
10. Use the remaining positions (e.g. ‘21-400’) and click ‘Fit’ again. These fiducial markers will be used to create the piecewise affine map.

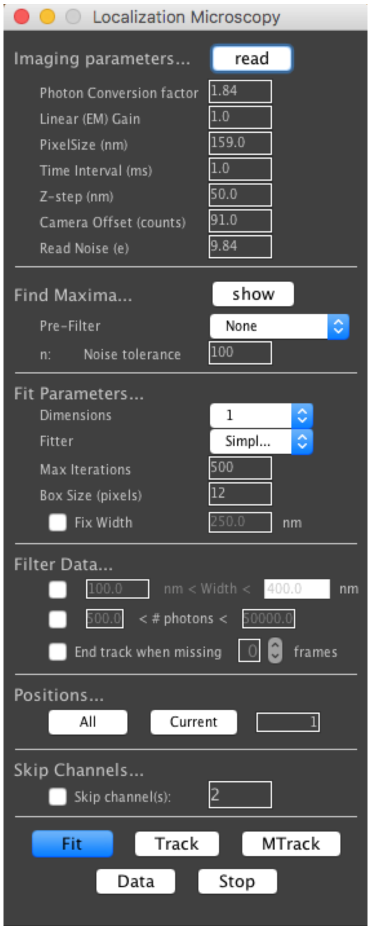

11. Open the data with fiducial markers that were acquired **after** to the sample of interest as described in step 6. This data can be used to test your map (determine the TRE as described in Materials and Methods).
12. Set ‘Positions’ to ‘1-400’ if you acquired a 20x20 grid and click ‘Fit’.
13. Now open a movie/image with the sample of interest as described in step 6. In the ‘Localization Microscopy’ plugin you may want to adjust the ‘Noise tolerance. Moreover, change ‘Positions’ to ‘1’ since you likely either acquired only a single image or a time-lapse movie and did not move to other positions. Then click ‘Fit’.
14. Repeat step 13 for all movies with your sample of interest.

b) Image registration

15. Select the data that you want to use for your affine map (typically the data of fiducials that you fitted in step 9) by clicking on the corresponding row.
16. In ‘2-Color’ select ‘Affine’ from the drop-down menu and use a radius of 2,000 nm. Then click ‘2C Reference’.
17. Select all other data including fiducials that will be used as your piecewise affine map, and to test your map as well as all datasets with your sample of interest. Then click ‘Correct’ in ‘2-Color’. This will create new rows with the affine corrected data.
18. Now, select the affine corrected version of your data that will be used as the piecewise affine map. Often the maps will have a few fiducials that have very different two-color distances than most others and might throw off the map. To remove those use ‘Pair Filters’ in the ‘Gaussian tracking data’ window with the following setting: Max dist(nm) = 50 nm, Max sigma = 2, # Quadrants = 36. Then click ‘Filter Now’. This will create a new row with data which is your affine corrected and outlier removed dataset to be used as the piecewise affine map.
19. Select the data created in step 18 and chose ‘Piecewise Affine’ from the ‘2-Color’ drop-down menu and a radius of 100 nm (unless you want to measure distances significantly larger than 100 nm, in which case you should set it approximately three-fold higher than the expected average distance). Then click ‘2C Reference’.
20. Now you want to set the parameters (Minimum and maximum number of fiducials, Maximum distance) for the piecewise affine map. Since we learnt that ‘10’ is the ideal number as minimum points, this value can not be changed. However, the maximum

number (default ‘100’) and maximum distance (default ‘2000’) can be adjusted. Therefore, create a custom script:

import edu.ucsf.valelab.gaussianfit.DataCollectionForm;

dcf = DataCollectionForm.getInstance();

dcf.setPieceWiseAffineParameters(100, 2000);

Click ‘Run’.

21. Next, select the affine corrected versions of the fiducial data that will be used to test the map and all affine corrected data of your sample of interest. Then click ‘2C Correct’. This command will carry out the piecewise affine transform to correct all your data.
22. At this point you may find it useful to save your data. Select all rows you want to save. Select ‘Text’ and click ‘Save’ in ‘General’ in the ‘Gaussian tracking data’ window. In case you want to load data, you can either click ‘Load’ or drag ‘n drop the ‘.txt’ files into the ‘Gaussian tracking data’ window. The binary format is described in detail here: https://micro-manager.org/wiki/Tagged_Spot_File_(tsf)_format.
23. To determine the registration error, select the piecewise affine corrected version of the fiducial data that will be used to test the map and click ‘List Pairs’ in the ‘Gaussian tracking data’ window and a new window (‘Pair display options’) will open. Then select ‘Show X-Y distance histogram (registration error)’. You may also want to adjust ‘Maximum distance’ to typically 30. Then click ‘OK’. This will open histograms with the registration error in x and y. These can be saved as a ‘.png’ by right click → ‘Save as …’. In addition, a window that shows the final calculated registration error will open and can be saved as a ‘.txt’ by right clicking → ‘Save As...’.
24. In case you want to filter any of your files by number of photons (Intensity) or localization error (Sigma), select the corresponding rows. Then type the desired values in ‘Filters’ in the ‘Gaussian tracking data’ window and click ‘Filter Now’. As before, this will create new data/rows with your filtered datasets.

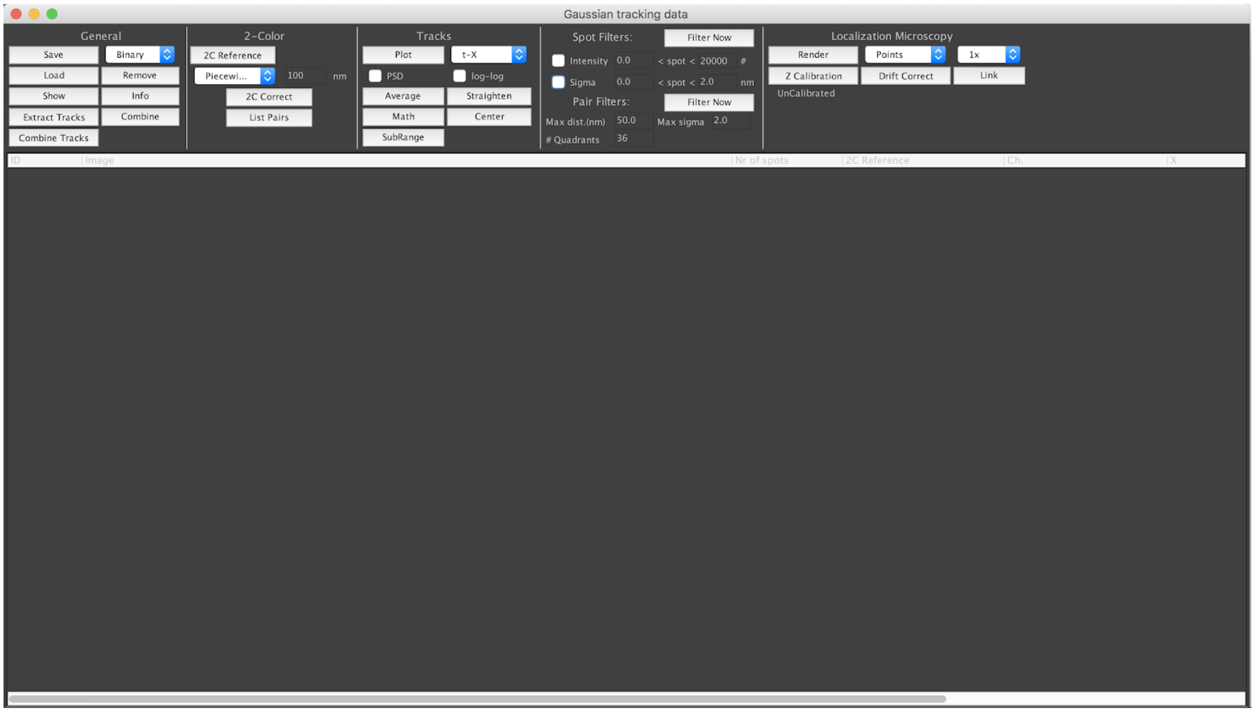

d) Data analysis and distance determination

25. If you have more than one movie/image of your sample of interest, you may want to combine all of them now. Select all desired files/rows and click ‘Combine’ in ‘General’ in the ‘Gaussian tracking data’ window. This will create a data set/row with all files combined. However, when files get combined they still keep unique identifiers. In this case different movies will be assigned different positions. You can see this by selecting the file with the combined data and by clicking ‘Show’. This will open a window with all relevant values like frame number, channel, position, intensity (# of photons), and sigma (localization error). Note, this is the exact same data, that gets saved, when you click ‘Save’.

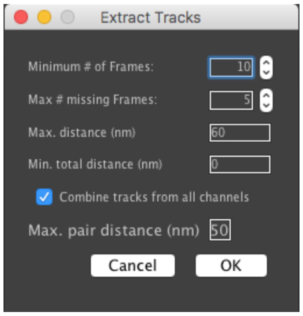

26. This step is optional and only required if you want to extract tracks with defined parameters (remove outliers). A ‘Track’ here refers to the data (localizations) of the same spot that was imaged over multiple times (time-lapse). Thus, if you acquired a single image only, you should skip this step.
Select either individual files of your data of interest or the combined version as created in step 25. Then click ‘Extract Tracks’. This will open a window as shown above. Here is what each of the settings mean:

a. ‘Minimum # of frames’: Say you acquired movies with 20 frames, then you may want to select only spots that have more than 15 frames total to remove spots that bleached earlier or that blinked a lot.

b. ‘Max # of missing Frames’: Define how many frames can be missing (due to blinking) between the first and last localization.

c. ‘Max distance (nm)’: This determines how far localization n can be away from localization n-1 (e.g. it should not jump by 1,000 nm).

d. ‘Min total distance (nm)’: Irrelevant here. Set to ‘0’.

e. ‘Combine tracks from channels’: If selected, it will combine tracks from channel 1 and 2 which have localizations in at least one and the same frame that are less than ‘Max. pair distances (nm)’ apart.

f. ‘Max. pair distances (nm)’: See ‘Combine tracks from channels’.

Select your values. If you run any of the example data that we provided, use the values as listed in Table S6. Then click ‘OK’. This will create new rows for each track. Typically, you will only care about tracks that have both, channel 1 and 2. These will be listed in the fifth column in the table of the ‘Gaussian tracking data’ window as ‘1,2’. Now, you can combine all tracks that have both channels by selecting these and by clicking ‘Combine’ (similar to step 25).

27. Now, you can determine the distance. Select either data as combined in step 25, or the ‘Extract Tracks’ purified data from step 26, or individual tracks of a single molecule that you created in step 26. Then click ‘List Pairs’ and a new window (‘Pair display options’) will open. Here you have various options discussed below (all can be executed at the same time):

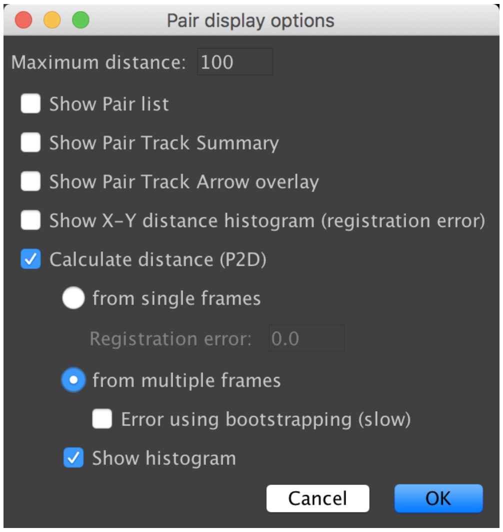

a. ‘Maximum distance’: Provide the cutoff for the maximum distance of a dye pair. This should not be larger than the value you used for the piecewise affine map in step 19.

b. ‘Show Pair list’: If selected, this will open a window with values like all pairwise distances, position, frame number, and localization errors of individual dye pairs. You can save this table by right click → ‘Save As...’.

c. ‘Show Pair Track Summary’: Can only be used if more than one frame per position was acquired (movie but no single image). If selected, this will open a window with values of vector distance averages and their standard deviations, number of frames, localization errors, and more. You can save this table by right click → ‘Save As...’.
d. ‘Show Pair Track Arrow Overlay’: Draws arrows into the micrograph to show orientation and magnitude of distance of individual pairwise distances. By orientations we mean an arrow pointing from channel 1 to channel 2. This can be useful to quickly evaluate if the registration map creates any larger, correlated patches when plotting the arrows for the corrected fiducial markers that were acquired after the sample of interest.
e. Show X-Y distance histogram (registration error): see step 23
f. ‘Calculate distance (P2D)’: This function has multiple subcategories and will calculate distances with the corresponding fit:
    i. ‘From single frames’: executes Sigma-P2D (you can provide the registration error as determined above)
    ii. ‘From multiple frames’: executes Vector-P2D (you can get an error from bootstrapping)
    iii. ‘Show histogram’: Will plot a histogram with the distance distribution and the selected fit(s). This can be saved as a ‘.png’ by right click → ‘Save as …’

Supplementary Information References

1. Edelstein A, Amodaj N, Hoover K, Vale R, Stuurman N (2010) Computer control of microscopes using µManager. *Curr Protoc Mol Biol* Chapter 14:Unit14.20.
2. Fitzpatrick JM, West JB (2001) The distribution of target registration error in rigid-body point-based registration. *IEEE Trans Med Imaging* 20(9):917–927.
3. Pitiot A, Malandain G, Bardinet E, Thompson PM (2003) Piecewise affine registration of biological images. *WBIR* (Springer), pp 91–101.
4. Mortensen KI, Churchman LS, Spudich JA, Flyvbjerg H (2010) Optimized localization analysis for single-molecule tracking and super-resolution microscopy. *Nat Methods* 7(5):377–381.
5. Pertsinidis A, Zhang Y, Chu S (2010) Subnanometre single-molecule localization, registration and distance measurements. *Nature* 466(7306):647–651.
6. Thompson RE, Larson DR, Webb WW (2002) Precise nanometer localization analysis for individual fluorescent probes. *Biophys J* 82(5):2775–2783.
7. Ober RJ, Ram S, Ward ES (2004) Localization accuracy in single-molecule microscopy. *Biophys J* 86(2):1185–1200.
8. Mortensen KI, Sung J, Flyvbjerg H, Spudich JA (2015) Optimized measurements of separations and angles between intra-molecular fluorescent markers. *Nat Commun* 6:8621.
9. Churchman LS, Flyvbjerg H, Spudich JA (2006) A non-Gaussian distribution quantifies distances measured with fluorescence localization techniques. *Biophys J* 90(2):668–671.
10. Stallinga S, Rieger B (2012) The effect of background on localization uncertainty in single emitter imaging. *2012 9th IEEE International Symposium on Biomedical Imaging (ISBI)*, pp 988–991.

Supplementary Information - Reviewers Comments

Reviewer #1's comments and author response:

Comments to Author :

In this manuscript, Niekamp et al. introduce analysis methods that advance the state of the art for measuring molecular distances by colocalizing two different fluorophores. The performance of the methods is analyzed using simulations, validated using single-molecule experiments, and applied to a novel measurement addressing an outstanding question in the dynein field. Building on prior work from the Spudich and Flyvbjerg groups, they show that the useful range of SHReC can be extended to shorter distances using two approaches - fits to distance distributions can be improved by incorporating independent information about localization error ("Sigma-P2D"), and vector-averaging over multiple frames can be productively combined with fits to rigorous distance distributions ("Vector-P2D"). Combined with optimized registration methods presented here, these approaches convincingly enable robust determination of mean fluorophore-to-fluorophore distances in the 8-20 nm range using a standard TIRF microscope. While the technical insights here might each be considered incremental in isolation, the overall outcome is an impressive and important advance that will have a strong impact on the single-molecule measurement field, enabled by the carefully detailed methodology documented by the authors and the software they will make available to the community. The kinesin measurement in particular provides a striking example of an accurate SHReC measurement at short distance scales. The dynein capstone is a good example of a problem that is not easily addressed using other available measurements, and the authors appropriately perform extensive complementary experiments to test their interpretation of the SHReC result.

This is a strong paper and the evidence largely supports the conclusions drawn (I would have entered "mostly" for question 6 if the option were available). I think it could be further improved by addressing the following questions.

1. Although the authors introduce Vector-P2D motivated by applications to heterogeneous samples, they also recommend using Vector-P2D for homogeneous samples in cases where enough frames are available for each molecule. For high enough values of sigma/d Vector-P2D will of course suffer from the same category of problem as P2D, yielding erroneous distance measurements (Fig. 4c), which the authors could discuss. In the special case of homogeneous samples, is there an opportunity for further improvement by combining the ideas in Sigma-P2D and Vector-P2D, making "Sigma-Vector-P2D"? That is, with independent determination of localization error and propagation through the vector averaging over frames, can the width of the distribution be removed as a fitting parameter in order to push the usability of Vector-P2D to higher values of sigma/d? Having introduced these two approaches it would seem natural to combine them; it would be great to see a discussion of the feasibility and/or utility of that.

We fully agree that Vector-P2D will encounter similar limitations as P2D with higher ratios of sigma/d. However, for the case of homogeneous samples, the vector averaging approach reduces the width of the distribution significantly (see Fig. 4b). Thus, Vector-P2D can tolerate higher ratios of sigma/d than P2D can (see Fig. 4 and Fig. S12). We now commented on this more extensively in the discussion section (see page 15) and when introducing Vector-P2D (see page 10). Also see point 4.

We implemented and tested the suggested "Vector-Sigma-P2D" method. We created 100 Monte Carlo simulated datasets for samples that are homogeneous in distance with either 5 or 20 frames and 1,000 particles. Using these, we performed the regular Sigma-P2D fitting and "Vector-Sigma-P2D". For the "Vector-Sigma-P2D" we first calculated the vector average distance for each particle (as in Vector-P2D) and propagated (standard error of the mean) the corresponding localization errors so that these could be used in a subsequent Sigma-P2D fit as a fixed parameter. As expected, the distance distributions of "Vector-Sigma-P2D" are narrower than the distance distributions of Sigma-P2D. Overall, the "Vector-Sigma-P2D" fit results in smaller error bars than the Sigma-P2D fit. However, the average distance discrepancy for "Vector-Sigma-P2D" was shifted to higher values for larger ratios of distance uncertainty over distance (sigma/d) and thus less accurate than Sigma-P2D (see figure below). Hence, we decided not to include "Vector-Sigma-P2D" as a method in our manuscript.

We note that performing the "Vector-Sigma-P2D" the other way round, that is first performing Sigma-P2D and then vector averaging ("Sigma-Vector-P2D"), does not work. Say one performs Sigma-P2D on individual particles that were imaged for 20 frames to determine the distances of each particle separately. Taking all of these distances of individual particles is then equivalent to single frame observations and thus can not be fit with the vector averaging method since it requires two frames of more. Nevertheless, one could perform another P2D fit on this data, in which the distance and the distance uncertainty are both used as fitting parameters. This could be called something like "Sigma-P2D-P2D". We also tested this method and it performed worse than Sigma-P2D alone and thus we did not include it in the manuscript.

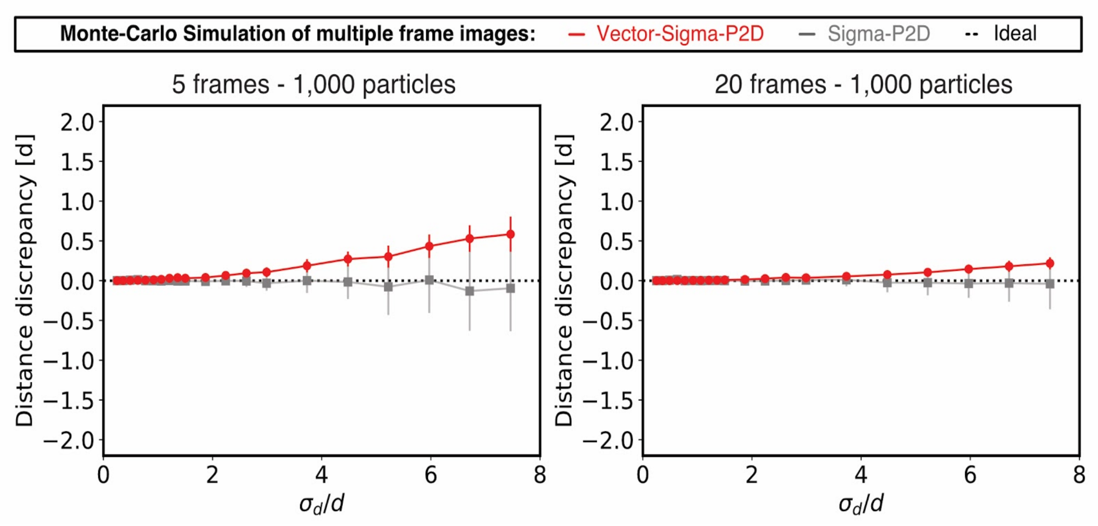

2. Throughout the paper the authors include plots of average discrepancy vs sigma/d, averaged over repeated simulations, to evaluate various analysis methods. I don't think it is explicitly stated what the errors bars in these plots represent - are these the standard deviations over repeated simulations? This should be labeled clearly as it is an important metric for readers considering the applicability of the methods to different situations. The authors could also consider providing inserts that focus on the most experimentally relevant regions of these plots, which cover a wide range of sigma/d and associated discrepancies.

This is a very good point as we forgot to list how the errors were calculated. Indeed, the error bars in Fig. 3 and 4 as well as Fig. S12, S13, S17 and S18 are the standard deviation over the repeated simulations. We added a sentence to each figure caption. We evaluated figures with insets, however, these became too busy and difficult to read.

3. In the plots described above, the authors consistently average results over just 10 simulations per data point. For many cases, over the wide parameter ranges presented, this is not enough sampling to determine the average discrepancies very well, so there is a lot of scatter in large regions of the plots. If the goal is really to precisely define the limits of these different approaches, why not use a much larger number of simulations and precisely determine the average (and standard deviation of) distance discrepancies for each data point?

We now generated more Monte Carlo simulated data (100 instead of 10 datasets). We then performed comparisons between different methods and updated the corresponding figures. We did this for the comparison between Sigma-P2D and P2D (Fig. 3c), Vector-P2D and Vector (Fig. 4c, Fig. S12 b-g, S13 (performance as function of sample heterogeneity)), the single-molecule analysis between Sigma-P2D and Vector (Fig. S17), and the comparison between MLE and NLLSQ fitting (Fig. S18).

4. The reader is left to gauge the domains of applicability by gazing at the plots described above, often as noted in plots with scatter due to undersampling. It would be valuable for the authors to summarize guidance about when different analyses are applicable, depending on sigma/d ratios and numbers of particles.

> We tried to provide some guidance with Fig. S16 and in the Discussion. We now extended this part in the Discussion (see pages 14-17) and defined some more stringent parameters (cut-offs) up to which each method is still good to use. Therefore we defined measurements as reliable when they resulted in an average distance discrepancy of less than 20% from the true distance with a standard deviation of less than 30% of the true distance. We also added a table in Fig. S16 that shows under which conditions (number of particles, number of frames and sigma/d ratio) Vector-P2D and Sigma-P2D can be used.

5. The authors discuss anticipated applications to dynamic measurements, but much of the paper focuses on methods for extracting average values from ensembles rather than measurements on individual molecules. Can the authors comment at more length in the main text on which aspects of their advances will be helpful for dynamic measurements?

> We now added a few more sentences in the Discussion section (page 16/17) where we discuss how our new methods could be advantageous for dynamic measurements of single molecules.

6. The issue above is briefly addressed in Fig. S17, where the authors compare Vector-P2D and Sigma-P2D for a single molecule. It should be clearly explained what "Vector-P2D" means for a single molecule here - is that just the single vector average over the frames recorded (in which case there is no "P2D" fit involved)? The specific claim of how many frames are required for Vector-P2D to do better than Sigma-P2D is not so convincingly presented here, again due to the very small number of simulations - certainly this plot could make good use of averaging over a larger number of simulations.

> This is an important point and we failed to describe clearly what Vector-P2D means in the case of a single particle. Since only single particles are analyzed, Vector and Vector-P2D are equivalent, because there is only one data point that can be fitted with the P2D function after vector averaging. Thus, we only used the Vector method for this case. We reran all Monte Carlo simulations and now used 100 instead of 10 datasets (see point 3). Moreover, we compared Sigma-P2D and Vector for different conditions of distance uncertainty over distance (see Fig. S17). With new data, we actually see that Sigma-P2D performs better than or at least equally well as Vector (smaller distance discrepancy) for all conditions when distance distributions of single particles and not ensembles are analyzed. Thus, our previous assessment that Vector can be better than Sigma-P2D for single particles was wrong and using a larger dataset helped to clarify this point. We used this data also to provide a better guideline for users (see Fig. S16

and response to point 4). We also updated this in the discussion section (see pages 15) and in the figure caption (Fig. S17).

7. The positions of fluorophores used for the authors' analysis are here determined using Gaussian fits to the image data. The authors should discuss the conditions (focus, dye mobility, etc.) under which this can be done without introducing important discrepancies in distances measured (and relate these conditions to the experiments performed). There is of course considerable literature (including work referenced here and work from e.g. the Moerner group) on how fixed dipoles or fluorophores with partially restricted mobility can lead to asymmetric PSFs and thus systematic localization errors when not appropriately fit.

We discussed the importance of focus, drift, and sample localization for the image registration process with Tetraspeck beads and now also added a similar discussion for the distance measurements (see page 15/16): As for the image registration, a high quality autofocus system is essential for two-color distance measurements since the image registration, and therewith the distance measurement, changes with focus. Thus, imaging of fiducial markers for image registration and sample of interest on the same slide (Fig. S1) is necessary. Restricted dye mobility causes changes in the point spread function leading to systematic localization errors and incorrect distance measurements. We observed a “normal” point spread function shape in all our samples, and also used intensity comparisons between linearly and circularly polarized light to ascertain full dye mobility.

8. Minor: I would advise caution in defining the domain of competing methods that the authors' analysis should be (favorably) compared to, given the large space of single-molecule methods that can be used for distance measurements. E.g. in Figure 1a could retitle "Current single-molecule localization techniques" with "Current single-fluorophore colocalization techniques".

We fully agree and should have been more careful with terminology. We now changed Fig. 1a as suggested and also updated the terms throughout the manuscript.

Reviewer #1's comments after revision:

Stuurman and coworkers have improved their manuscript through substantial revisions that were very responsive to questions raised by both referees. I am satisfied that they have addressed the issues raised in my original review.

Reviewer #2's comments and author response:

Comments to Author:
The authors devised a method to determine the registration error between two colors, then used these measurements to greatly improve on two different single-molecule analysis methods, creating a pipeline enabling distance measurements between fluorophores in the currently unresolved 8-25 nm range and beyond. Overall, this is an important advance that will facilitate a number of new studies that were previously not possible.
This paper will be of general interest, as commendably, an emphasis has been placed on enabling broad implementation of this technique by others.

No additional experiments are necessary for publication; however, guidance on the limits of this technique (suggested by the authors' simulations) is essential for the rigorous adoption by others. Below are some clarifications that might assist in the reader's understanding of this paper. Additionally, a number of changes to figure legends would help comprehension of the supplemental material.

General comments:

Many figures would be improved by captions motivating the experiments and a brief analysis of the results, especially in the supplement. Currently, experiments in the supplement are introduced in a combination of the main text, the methods section, and supplemental notes. Consequently, it is difficult to follow what motivates many of these figures and what conclusions to take away from the data. Ideally, the figure legends would summarize the key conclusions from each portion of the figure.
As discussed in more detail in the specific comments below, it should be explained why the distance discrepancy always trends to -1 with no error term with increasing noise in the author's simulations. (see comments regarding figure 4 and S12)

> We added a more detailed summary/discussion of the key conclusions to the figures and tried to make it easier to follow. For how we changed things in many figures, see comments below.

Specific comments:

Page 6:
"With the corrected second fiducial marker dataset, we then calculated the target registration error (TRE)"
The reference to figure S2 in the main text should refer to figure 2 instead- figure S2 deals with the comparison between TRE and FRE.

> This is a good point. We made the change accordingly.

Page 7:
"...we routinely (76%) achieved registration accuracy..."
Please comment on why registration failed in 24% of experiments- do the authors have a sense of what causes registration to fail?

> Yes, in almost all cases the registration failed because of a slight change in focus between or during the acquisition of the first and second dataset of fiducial markers. We added such a comment of page 7.

Page 7:
"(i.e. < 1 nm sample movement for approximately 1 sec)"
As "successful execution" of this technique relies on this criterium, it would be useful to provide advice for users on how to ensure that this condition is met. This seems particularly important because presumably, this is not only true of movement within the sample but also movement of the sample or stage relative to the objective (since the registration correction is non-uniform across the field).

> This is an excellent point and we should have elaborated further on this. Now, we added some information on how this stabilization can be achieved (see page 7 and Methods).

Page 8:
"Given that our new method can refine measurements made over all distances for which a distance uncertainty can be determined..."
Please give the range of distances (in nm) this corresponds to.

> We added the information on page 9. Also, see comments to “Page 13”.

Page 10:
"Even for cases where σcon is twice as large as the true distance d, we still recovered the correct population average (Fig. S13)."
What are the criteria for recovering the correct population average and how do the data in figure S13, specifically the 5 and 10 frame cases, meet these criteria? They appear to diverge significantly from the ideal result as σcon increases. Or this criteria only met in the 20 frame case? If so, a small clarification would help readers get this point.

> Similar to the comparison of for instance Sigma-P2D vs. P2D we judged the performance by the discrepancy of the measured distance from the predicted distance. However, we did not differentiate between different cases of number of frames. It is true that the 5 and 10 frame cases perform worse that the 20 frame cases for very heterogeneous data. Generally speaking, the more heterogeneous the sample the more frames have to be recorded to achieve accurate results which in this case is defined as

20% offset of the measured distance from the predicted distance. We now added a clarification on page 11.

Page 13:
"...we show that our techniques enable distance measurements from ~2 nm to hundreds of nanometers..."
Please explain where these bounds come from and how those who utilize this technique can determine the bounds for their own input images.

As we have shown in Fig. 4 c, the successful execution of Vector-P2D is insensitive to the actual distance but depends on the ratio of distance uncertainty over distance. In our simulations, we used a true distance of 10 nm. However, we tested if Vector-P2D can also resolve distances of 2 nm, 10 nm, 20 nm, 50 nm, 100 nm, 200 nm, and 500 nm by Monte-Carlo simulation but did not show the data. We now included this data in Fig. S12h, i. We also added a sentence about this in the manuscript (see page 11). Moreover, we may not have explained well enough why we used units of distance discrepancy [d] instead of an actual distance value in nanometer (as we could see from your comment for Fig. 4). We now added a clarification on page 9 and more detailed clarification in the corresponding figure captions (Fig. 3, 4, S12, S13, S17, and S18).

Figure 2:
Please provide the histogram of target registration error before the piecewise affine correction (i.e. from the data in panels c and d). This will allow the reader to evaluate the contribution of the piecewise affine registration step to the overall registration performance.

We now added the histogram of the target registration error for the affine registration in Fig. 2.

Figure 4:
Panel c: In the 20 frame data, why does error bar size increase with increasing sigma (as expected) and then suddenly decrease at $\sigma d/d = 6$ (see also comments on figure S12)? Can the authors comment if this reflects on some limit of the technique?

This is a good point and we should have clarified better. The reason that the error bar disappears is that we evaluated the performance of Vector-P2D and Vector by calculating the average distance discrepancy. Therefore we subtracted the expected distance from the measured distance and normalized by the expected distance. Thus, values around -1.0 represent cases for which we measured 0 nm and for which we find very small error bars showing that this is very reproducible. Large error bars typically indicate bimodal cases for which we measured both distances that are similar to the expected distance and distances that are much smaller than the expected distance (around 0 nm). Hence, the increasing size of error bars with increasing sigma shows that

the fitting outcome is becoming more and more bimodal until it collapses to one side (measuring distances of around 0 nm).

The meaning of units of distance discrepancy [d] is not clear and should be explained more thoroughly.

We now added a better definition for the distance discrepancy (pages 9 and 11). Also, see comment to Page 13 and Fig. 4.

Figure 5:
Panel a: This figure should be supplemented with additional details explaining the non-labeled components. For example, the moving protrusion from AAA1 - What does the purple line represent? Please also define abbreviations in the figure, and importantly, label which dynein is apo and vi.

We now provide a label for the purple line and also defined the abbreviations in the figure caption. As for the labeling which dynein is apo and ADP-vi, we already added labels on top of each of the two dynein cartoons in Fig. 5a.

Figure 5 and associated main text:
It took me a while to understand the same disorder/order connection the authors did from between the difference in distances measured, and lack of visible stalks in the cryo data. What I had to realize what that, while one can understand that disorder can make the stalk disappear in EM, in the SA-immobilized samples, both ends are constrained - hence disorder reduces length, order increases it. A simple sentence or two making this clarification explicitly would help the reader make the connection as well.

We now added additional clarification in the main text and fully agree that this is needed (see page 13).

S2:
This figure not substantially motivated in the text. Specifically, why is a different dataset used in this figure than in figure 2, and in particular one that yields a very different outcome than the successful registration in figure 2? Is the goal to show a condition in which target registration has failed, and in which this failure is detected by TRE and not FRE?

We agree that Fig. S2 is not extensively discussed in the text and that we did not motivate this figure well. Therefore we now added a few sentences in the figure caption as well as in the Materials and Methods section.
We indeed used a different dataset (compared to Fig. 2 with a successful image registration) for which the image registration failed (likely due to an instability in the focus between or during the acquisition of the first and second fiducial marker dataset)

to show that the TRE can detect the failure and that FRE would not. We now added this description to the figure caption as well.

S3:
A bit more explanation of the exact meaning of the maximum and minimum fiducial and the maximum distance in the context of the registration algorithm is unclear. Define please. Panels m-r are also appear highly redundant with panels a-f, and may not be necessary.

We now added a more detailed description (in the figure caption) of the meaning of the maximum and minimum fiducial number and the maximum distance in the context of the image registration process.
We used the 10 by 10 um binning for the evaluation of a very large parameter set for piecewise image registration as we described and have shown in Fig. S4. However, we agree that panels m-r convey very similar information to a-f. Thus, we now removed these panels. Moreover, we now evaluated the performance of various parameters for image registration (Fig. S4) without binning. The results, for which parameter setting are the best for image registration, are very similar.

S6:
The caption for panel d does not make sufficiently clear how panel d differs from panel a. Incorporating the information in supplementary note 1 that grids are overlapping would aid understanding. Additionally, if the data for multiple overlapping beads is shown in panels E and F, something should be done to distinguish the data for each bead (ex. different point shapes).

We now added a clarification in the figure caption. We also added more information in Supplementary Note 1, and changed the bead representation in e and f so that the positions of one bead are shown as a circles and the positions of the other bead are shown as a squares.

S12:
As in figure 4c, there is little to no error at high $\sigma d/d$ values- why? In addition, in many panels in this figure, as in the main text, the analysis always appears to end up at a distance discrepancy of -1.0 with no error. What causes this to happen? This is an unexpected behavior. Such systematic error, seemingly on the same order of magnitude as the distance being measured, may disqualify this measurement technique in certain $\sigma d/d$ (~>2-3) regimes.

The reason that the error bar disappears is that we evaluated the performance of Vector-P2D and Vector by calculating the average distance discrepancy. To do so, the expected distance is subtracted from the measured distance and normalized by the expected distance. Thus, values around -1.0 represent cases for which we measured 0 nm and for which we find very small error bars showing that this is very reproducible. Large error bars typically indicate bimodal cases for which we measured both distances that are similar to the expected distance and distances that are much smaller than the

expected distance (around 0 nm). Hence, the increasing size of error bars with increasing sigma shows that the fitting outcome is becoming more and more bimodal until it collapses to one side (measuring distances of around 0 nm). This means that the method is highly error prone for conditions that lead to -1.0 values with small error bars.

S14:
Panels e and f may be unnecessary- they do not add additional information beyond what is shown in the beginning of the figure. If the authors wish to keep the data in panel F, something should be done to distinguish the data for the different rulers (ex. different point shapes) as in figure S6.

We agree and removed panels e and f.

S17:
The figure states that Vector-P2D works better for frame numbers of 7 and up, but elsewhere in the paper a cutoff of 5 frames is quoted (e.g. figure S16). The authors should be consistent about the recommended cutoff- it seems like a cutoff of 7 is the best-motivated choice given the data presented here.

This is a valid point. As described in the comments to reviewer #1, we reran all Monte Carlo simulations and now used 100 instead of 10 datasets. Moreover, we compared Sigma-P2D and Vector (which is the same as Vector-P2D for single particles) for different conditions of distance uncertainty over distance (see Fig. S17). With this new data we actually see that Sigma-P2D always performs better than or at least equally well as Vector for single particles. We added a comment about this in the manuscript (see page 15) and in the figure caption. Moreover, we included this in our guideline for when to use which method (Fig. S16). See also responds to point 6 of reviewer #1.

S18:
The data in panel g contradicts the claim on page 24 ("...NLLSQ fitting outperforms MLE in all conditions where random background noise was added (Fig. S18)").
These data also display the same systematic errors seen in figure 4c and S12 (data trending to -1 discrepancy with no error).
Why, if NLLSQ outperforms MLE, was MLE fitting used for sigma-P2D?

We overstated the performance of NLLSQ. What we are trying to say is that the fitting with NLLSQ is better or at least as good as the MLE fitting (for Vector-P2D), as long as background noise was below 5%. At higher levels of background noise both methods failed to recover the true distance. We corrected this in the manuscript (see page 26).

As for the systematic error, see comments for Figure 4c.

We noticed that individual molecules rarely have any outliers (see individual molecules of nanorulers in Fig. 4e) and since Sigma-P2D is fitting each molecule separately (every molecule has different localization errors) we did not use NLLSQ for Sigma-P2D. We also compared (with Monte Carlo simulated data) fitting of Sigma-P2D with NLLSQ and MLE and did not observe any difference in fitting accuracy. However, the MLE fitting is much faster and thus we decided to use MLE for Sigma-P2D.

Reviewer #2's comments after revision:

The authors have addressed all my points, and I have no further concerns. I believe this manuscript is ready for (and worthy of) publication.
